# Evidence of what works to support and sustain care at home for people with dementia: a literature review with a systematic approach

**DOI:** 10.1186/s12877-015-0053-9

**Published:** 2015-05-13

**Authors:** Alison Dawson, Alison Bowes, Fiona Kelly, Kari Velzke, Richard Ward

**Affiliations:** 1 School of Applied Social Science, University of Stirling, Stirling, FK9 4LA UK; 2 Bournemouth University Dementia Institute (BUDI), Bournemouth University, Bournemouth, BH12 UK

**Keywords:** Dementia, Care at home, Services, Support

## Abstract

**Background:**

This paper synthesises research evidence about the effectiveness of services intended to support and sustain people with dementia to live at home, including supporting carers. The review was commissioned to support an inspection regime and identifies the current state of scientific knowledge regarding appropriate and effective services in relation to a set of key outcomes derived from Scottish policy, inspection practice and standards. However, emphases on care at home and reduction in the use of institutional long term care are common to many international policy contexts and welfare regimes.

**Methods:**

Systematic searches of relevant electronic bibliographic databases crossing medical, psychological and social scientific literatures (CINAHL, IngentaConnect, Medline, ProQuest, PsychINFO and Web of Science) in November 2012 were followed by structured review and full-text evaluation processes, the latter using methodology-appropriate quality assessment criteria drawing on established protocols.

**Results:**

Of 131 publications evaluated, 56 were assessed to be of ‘high’ quality, 62 of ‘medium’ quality and 13 of ‘low’ quality. Evaluations identified weaknesses in many published accounts of research, including lack of methodological detail and failure to evidence conclusions. Thematic analysis revealed multiple gaps in the evidence base, including in relation to take-up and use of self-directed support by people with dementia, use of rapid response teams and other multidisciplinary approaches, use of technology to support community-dwelling people with dementia, and support for people without access to unpaid or informal support.

**Conclusions:**

In many areas, policy and practice developments are proceeding on a limited evidence base. Key issues affecting substantial numbers of existing studies include: poorly designed and overly narrowly focused studies; variability and uncertainty in outcome measurement; lack of focus on the perspectives of people with dementia and supporters; and failure to understanding the complexities of living with dementia, and of the kinds of multifactorial interventions needed to provide holistic and effective support. Weaknesses in the evidence base present challenges both to practitioners looking for guidance on how best to design and deliver evidence-based services to support people living with dementia in the community and their carers and to those charged with the inspection of services.

**Electronic supplementary material:**

The online version of this article (doi:10.1186/s12877-015-0053-9) contains supplementary material, which is available to authorized users.

## Background

How best to support increasing numbers of people with dementia is a challenge for societies around the world. It is estimated that the total prevalence rate of dementia in people aged 65+ in the UK is 7.1% and that by 2015 there will be 850,000 people living with dementia [1]. A prevalence rate of 11% has been reported for the 65+ age group in the USA, equating to 5 million people living with dementia [2]; a recent pooled analysis of seven high quality European studies suggested a total prevalence rate of dementia across EU27 countries of 7.23% [3]; and in 2013 an estimated 27.8 million people (62% of all people with dementia) were living in low or middle income countries [4]. In most instances, a large proportion of people will be living in standard housing stock: for example, in the UK around two thirds of people with dementia will be living in their own homes [1] and in Australia, 70% of an estimated 298,000 people with dementia in 2011 lived in the community [5].

The literature describing and analysing services which support and sustain people with dementia living in their own homes is burgeoning. Expansion in the number and type of such services is partly driven by policy and practice which are increasingly emphasising the need to support people to live in their own homes in the face of growing numbers of people living with dementia. For example, the UK ‘Prime Minister’s challenge on dementia: Delivering major improvements in dementia care and research by 2015’ states unequivocally that ‘Failure to act will mean our health and social care services will struggle under the pressure of increasing numbers of people with dementia’ [6]. Also, and more importantly, service development is being driven by commitments to ageing in place, in accordance with the preferences of people themselves. In 2012, Australian ministers agreed to make dementia a ‘National Health Priority Area’ in recognition of ‘the increased burden of disease and the opportunities to make significant gains in the health status and well-being of people with dementia and their carers and families’ [7].

This paper synthesises research evidence about the effectiveness of services intended to support and sustain community-dwelling people with dementia and their carers. A systematically conducted review of research-based publications identified the current state of scientific knowledge regarding appropriate and effective services for people with dementia and their family caregivers in relation to a set of key outcomes derived from documents detailing recent Scottish policy [8,9] and guiding Scottish inspection practice (including relevant standards [10,11] and Statutory Performance Indicators (SPIs) [12] (see Table 1, below). The emphasis on care at home and the reduction in the use of institutional long term care is however common to many welfare regimes and particularly pertinent for the rest of the UK, where the policy emphasis is similar [13].

**Table 1:** **Key outcomes for people with dementia and their carers informing the literature review**

Key outcomes		
●	Prevention of unnecessary hospital admission	●Management of medication at home
●	Prevention of delayed discharge from hospital	●Delivery of community nursing
●	Reducing lengths of hospital stay	●Carer support
●	Effective discharge from hospital	●Self directed support
●	Consistency and quality of home care delivery (including staff training, staff support)	

Services which sustain and support people with dementia to live in their own homes do not operate in isolation: people with dementia and their caregivers are likely to have a range of needs, e.g. in relation to health care, transport, and support with cognitive or emotional tasks [5] and so are likely to be simultaneously engaged with and using health services and other community-based care services [14]. Since this research was specifically commissioned to inform a quality inspection regime, it was necessary for the review to focus not only on services specifically providing delivery of care at home, but also on the interaction of those with other health and social care services and on contextual matters relating to the achievement of key outcomes identified by the inspection agency and detailed in Table 1 above. Our approach considers both the systems and structures that are in place to support care, and the experiences and actions of people living with dementia, providing a broad narrative overview and systematically derived quantification of the evidence base.

In the section which follows we set out our methods for this study. Following this, we summarise our findings by topic area. We then present a discussion of emerging issues, cross-cutting themes and implications for practice before drawing our final conclusions.

## Methods

We aimed for a transparent and systematic process, whilst also ensuring a pragmatic approach in the light of available resources of time and funding. A PRISMA 2009 Checklist is provided as a supplementary document accompanying this paper.

### Search and selection strategy

**Figure 1:**
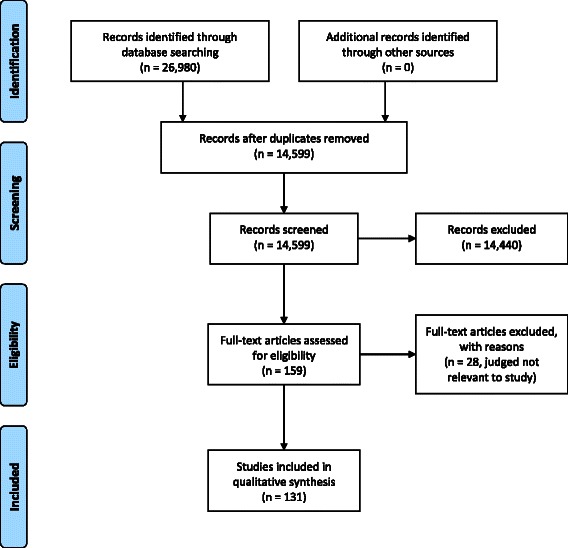
PRISMA diagram of the review process. This has been uploaded as a separate file.

The first step was a systematic search of relevant bibliographic databases to ensure the necessary broad coverage of areas of interest, crossing medical, psychological and social scientific literatures. We searched CINAHL, IngentaConnect, Medline, ProQuest, PsychINFO and Web of Science electronic databases. The searches took place in November 2012 and were restricted to English language sources, including international literature, published in 2002 or later. The intention of the searches was to identify, quantify and summarise the evidence base around those areas of interest to the commissioning body outlined above. Many of the topics of interest referred to recent innovations in service, and we expected that literature covering longer standing aspects of service provision would refer back to earlier work if relevant. The search terms were developed in collaboration with the commissioning body and informed by a set of key outcomes for people with dementia and their carers provided by them as described above and detailed in Table 1. The search terms used for the review are listed below. Figure 1 provides a PRISMA diagram of the review process.

### Literature review search terms (‘*’ denotes truncation symbol)

- (dementia OR Alzheimer*) and (care OR support) and (hospital admission*)
- (dementia OR Alzheimer*) and (care OR support) and (discharge*) [note: delayed, effective, supported discharge all covered by this string]
- (dementia OR Alzheimer*) and (care OR support) and (hospital stay*)
- (dementia OR Alzheimer*) and ((home OR domiciliary) AND (care OR support)) and (staff)
- (dementia OR Alzheimer*) and ((home OR domiciliary) AND care) and (hours OR evening* OR overnight OR weekend*) [from Care of Older People Information Set (COPIS)†]
- (dementia OR Alzheimer*) and (home) and (medicine* OR medicat*) and (management OR compliance OR adherence OR capacitance)
- (dementia OR Alzheimer*) and (community nurs*)
- (dementia OR Alzheimer*) and (carer*) and (support*)
- (dementia OR Alzheimer*) and ((direct payment*) OR (self directed OR self-directed) OR (indiv* budget*))
- (dementia OR Alzheimer*) and (personalisation OR personalization)
- (dementia OR Alzheimer*) and ((reablement OR re-ablement) OR (rehabilitation) OR (enablement))
- (dementia OR Alzheimer*) and ((intermediate care) OR (rapid response) OR (step up step down) OR (convalesc*) OR (progressive care) OR (hospital-at-home))
- (dementia OR Alzheimer*) and ((community OR local OR cottage) AND (hospital*))
- (dementia OR Alzheimer*) and ((post diagnos*) OR (post-diagnos*))
- (dementia OR Alzheimer*) and (day service*)
- (dementia OR Alzheimer*) and (community support)

†The Care of Older People Information Set (COPIS) is an ‘information set’ jointly developed by NHS QIS and SWIA containing seventeen key indicators that can be used to assess outcomes for older people and partnership working between health and social work services.

Following initial screening by title and then title plus abstract, 1763 references remained for consideration. Project resources precluded full text examination of this volume of literature. After consulting the commissioning body, we applied further inclusion criteria focusing on material published 2007 onwards, prioritising reviews of research, and emphasising the priority areas identified in Table 1. In total 310 items met these criteria to a greater or lesser extent. In conducting full text readings, we prioritised higher scoring items, but also items which covered the relevant areas of interest, ensuring the necessary breadth required by the commissioning body. On the basis of examination of the full texts, 131 publications covering UK and international research were included and underwent quality assessment, with 28 adjudged to be irrelevant to the study.

### Review and evaluation strategy

A team of readers reviewed the full texts and completed a structured report on each item read using the ‘Stirling literature review proforma’. This is an online proforma developed at the University of Stirling which allows the capture of bibliographic and content-related data and facilitates a research-design specific quality assessment of reviewed texts.

After recording basic information about the text, reviewers are asked to identify the research design used in the publication being reviewed, after which routing within the proforma takes the reviewer to a quality assessment section specific to that type of research, e.g. Randomised Controlled Trial (RCT), Qualitative study, Literature review, etc. The sections reproduce assessment criteria developed and in use elsewhere, for example Centre for Research and Development (CRD) Report No 4 [15] for RCTs and Controlled Clinical Trials (CCTs), Cochrane Effective Practice and Organisation of Care (EPOC) checklists [16] for Controlled Before and After studies (CBAs) and Interrupted Time Series studies (ITSs) and Critical Appraisal Skills Programme (CASP) assessment criteria [17] for Economic Evaluations and Literature Reviews, plus a section for ‘Other types of study’. Reviewers are guided through the criteria contained in the selected assessment section, and then asked to rate (and record their reasons for rating) the publication as of Low, Medium or High quality based on whether the full text has revealed major, important, or minor limitations in the study methodology as reported in the publication. The assessment produces a quality rating compared to the archetype for studies using the same research design and thus provides an indication of the degree of caution that should be attached to a study’s findings and conclusions: it does not allow for quality comparison across research designs.

Unlike other approaches to systematically reviewing literature which include specific research designs as an inclusion/exclusion criterion, this approach allows for the consideration of evidence gathered using the full range of different methodological approaches. Phenomena of interest in the present study such as the development of novel services or service users’ experience of existing services may not have been explored using the types of research designs privileged in other approaches to review, but evidence from other types of research may well exist, knowledge of which would be of value to the commissioning body and others. The approach taken in the Stirling literature review proforma is thus ideal for those occasions where the objective is to identify and understand the full range and scope of the available evidence base.

A sample of texts was double read to check inter-reviewer reliability. The proforma provided summaries of the content of all reviewed materials, data on a number of variables to facilitate later analysis of the reviewed literature, and a clearly defined quality assessment of each item. Data collected in the proforma were then used to group the literature thematically.

A full table of the included references and their quality assessments is provided as a supplementary document (see Additional file 1) and a PRISMA 2009 Checklist is also provided (see Additional file 2).

## Results

Of the 131 publications evaluated, 56 were assessed to be of ‘high’ quality, 62 of ‘medium’ quality and 13 of ‘low’ quality. Table 2 summarises the quantity and quality of literature identified and study types included, grouped under five main topic headings and, where used in the review, additional sub-topic headings. Column totals are higher than the total number of evaluated publications because the majority of references were relevant to multiple topics and sub-topics – especially in the case of research on informal (also known as unpaid) care. Work assessed as of low quality is not included in the table.

**Table 2:** **Quantity, quality and type of identified literature by topic heading**

Topic	Subtopic	Items classified as high quality (study types)	Items classified as medium quality (study types
Early intervention and post diagnostic services	Early intervention (anticipatory/on diagnosis)	19 (11 Literature reviews; 1 Cohort study; 7 Other (2 Program descriptions; Practice guidelines; test of MCI screening tools; Practice-based reflection; Policy analysis; Program evaluation))	18 (12 Literature reviews; 1 Economic evaluation; 3 Qualitative studies; 2 Other (Descriptive service overview, report of survey))
	Post diagnostic support	18 (9 Literature reviews; 1 Controlled clinical trial (CCT); 3 Qualitative studies; 5 Other (2 Program descriptions; practice-based reflection; Retrospective case review; Program evaluation))	23 (7 Literature reviews; 1 Randomised controlled trial (RCT); 2 Controlled clinical trials (CCT); 3 Qualitative studies; 1 Non-controlled Before and After study; 5 Other (2 Cross-sectional studies; 2 Service overviews; 2 Reports of surveys; Program evaluation; 2 Retrospective case reviews; Study protocol))
Community-based services supporting people with dementia living in their own homes	Self directed support	3 (1 Literature review; 1 Qualitative study; 1 Other (practice-based reflection))	3 (1 Literature review; 2 Other (Reports of survey; Program evaluation))
	Community-based support	16 (5 Literature reviews; 1 Controlled clinical trial (CCT); 4 Qualitative studies; 6 Other (Multi-method study; 2 Program evaluations; Practice-based reflection; Report of survey; Policy analysis))	14 (5 Literature reviews; 1 Controlled clinical trial (CCT); 1 Economic evaluation; 7 Other (Cross-sectional study; Service evaluation; Program evaluation; 2 Reports of surveys; Service description; Study protocol))
	Domiciliary support	11 (3 Literature reviews; 1 Controlled clinical trial (CCT); 4 Qualitative studies; 3 Other (Report of survey; Program evaluation; Policy analysis))	16 (5 Literature reviews; 3 Qualitative studies; 8 Other (2 Cross-sectional studies; 2 Reports of surveys; Program evaluation; Service evaluation; Service overview; Study protocol))
	Rapid response	4 (1 Literature review; 3 Other (Report of survey; Program evaluation; Retrospective case review))	4 (1 Qualitative study; 3 Other (Report of survey; Program evaluation; Service evaluation))
	Enablement, re-ablement and rehabilitation	16 (11 Literature reviews; 2 Qualitative studies; 3 Other (Report of survey; Program description; Program evaluation))	18 (13 Literature reviews; 1 Qualitative study; 5 Other (Retrospective case review; Program evaluation; 2 Reports of surveys))
	Managing medication	4 (1 Literature review; 1 Controlled clinical trial (CCT); 2 Qualitative studies)	7 (2 Literature reviews; 5 Other (Service evaluation; Service description; Study protocol; Report of survey; Retrospective drug use study))
	Day services	6 (4 Literature reviews; 2 Qualitative studies)	2(2 Other (Cross-sectional study; Report of survey)
Hospital-related areas of interest	Intermediate care	8 (4 Literature reviews; 1 Controlled clinical trial (CCT); 1 Qualitative study; 2 Other (Multi-method study; Policy analysis))	4 (1 Literature review; 1 Qualitative study; 2 Other (Program evaluation; Report of survey))
	Preventing unnecessary admission	13 (8 Literature reviews; 2 Qualitative studies; 3 Other (Case study; 2 Program evaluations))	10 (2 Literature reviews; 1 Qualitative study; 7 Other (Cross-sectional study; Service evaluation; Program evaluation; Retrospective case review; Report of survey; Service description; Study protocol))
	Community hospitals	4 (2 Literature reviews; 2 Other (Retrospective case review; Program evaluation))	2 (2 Other (Report of a survey; Program evaluation)
	Reductions in length of stay	6 (3 Literature reviews; 1 Cohort study; 2 Other (2 Program evaluations))	5 (1 Cohort study; 4 Other (2 Retrospective case reviews; Report of survey; Program evaluation))
	Discharge	5 (1 Literature review; 1 Cohort study; 3 Other (Multi-method study; 2 Program evaluations))	6 ((1 Literature review; 1 Cohort study; 1 Qualitative study; 3 Other (Cross-sectional study; Report of survey; Program evaluation))
Informal/unpaid care		27 (13 Literature reviews; 1 Controlled clinical trial (CCT); 6 Qualitative studies; 7 Other (Case study; Literature-based concept analysis; Meta-analysis; Multi-method study; Retrospective case review; Policy analysis))	25 (11 Literature reviews; 1 Randomised controlled trial (RCT); 1 Controlled clinical trial (CCT); 3 Qualitative studies; 9 Other (2 Cross-sectional studies; Study protocol; 3 Service descriptions; 2 Reports of surveys; Program evaluation))
Workforce and service delivery*			
	Joint working/ partnership working	16 (7 Literature reviews; 1 Controlled clinical trial (CCT); 4 Qualitative studies; 4 Other (Report of survey; 2 Program evaluations; Policy analysis))	11 (3 Literature reviews; 3 Qualitative studies; 5 Other (Cross-sectional study; Study protocol; Service description; Report of survey; Program evaluation))
	Integrated care/ teams	16 (7 Literature reviews; 1 Controlled clinical trial (CCT); 4 Qualitative studies; 4 Other (Report of survey; 2 Program evaluations; Policy analysis))	11 (3 Literature reviews; 1 Non-controlled Before and After study; 2 Qualitative studies; 5 Other (Cross-sectional study; Study protocol; Service description; Report of survey; Program evaluation))
	Consistency and quality of home care (staff training and support)	10 (2 Literature reviews; 4 Qualitative studies; 4 Other)	
(Literature-based concept analysis; Report of survey; Program evaluation; Policy analysis))	15 (7 Literature reviews; 1 Economic evaluation; 2 Qualitative studies; 5 Other		
(Study protocol; Service description; 2 Reports of surveys; Program evaluation)			
	Delivery of community nursing	5 (2 Literature reviews; 2 Qualitative studies; 1 Other)	
(Policy analysis)	6 (2 Literature reviews; 4 Other		
(Service evaluation; Study protocol; 2 Reports of surveys)			

*Literature on Community-based support and Day services was also examined for discussions of workforce issues as part of the consideration of ‘Workforce and service delivery’.

The findings below are presented by topic as set out in Table 2. We found considerable thematic overlap in the literature, indicating interconnectedness between areas of support for people living with dementia. For this reason, the same source may be referred to in connection with more than one topic or sub-topic. Our analysis has also been shaped by feedback from three full-day project workshops held between January and March 2013 and attended by a total of 38 managers and inspectors from the agency commissioning the research at which results from the literature review were presented and discussed.

In this review we have predominantly referred to materials assessed as of high quality: where we have referred to texts assessed as being of medium quality this is clearly indicated in the text by the insertion of ‘(M)’ in superscript following the reference number, e.g. ‘Doe et al.’s [X^(M)^] study’. The use of medium quality evidence in a transparent way is essential in this instance, where newer forms of service provision are being considered, and funding has frequently permitted only smaller scale studies to be conducted. Furthermore, where evidence to date is not yet of the highest quality, indications of efficacy are nonetheless worth noting as suggestive of potential for success.

### Early intervention and post-diagnostic support

Recent research has highlighted the ‘gap’ between predicted numbers of people with dementia based on prevalence rates and actual numbers with diagnoses of dementia across the UK [18]. Acknowledging this disparity, NHS Scotland and NHS England have both committed to targets to maintain or improve dementia diagnosis rates and improve the provision of immediate post-diagnostic support [19,20]. In Scotland post-diagnostic support will be informed by the Alzheimer Scotland ‘5 Pillars of Post-Diagnostic Support’ model [21] which highlights the need to ensure that people with dementia and their families get the information and support that they need to: understand the condition and manage symptoms; plan for future decision-making; make timely decisions about preferences for future care; maintain community connections; and access peer support.

However, the challenge is to both improve rates of diagnosis *and* bring forward the timing of the diagnosis. Chrisp et al.’s [22]^(M)^ study found mean time of 3 years from first thinking that something is amiss to receiving formal diagnosis and concluded that encouraging earlier contact with healthcare services offered the greatest potential for earlier diagnosis. Cultural factors and concerns over the availability of appropriate services may play a part in the timing of help-seeking: Moriarty et al. [23] found that people from Black and Minority Ethnic (BME) communities seek help with memory difficulties significantly later than White British people. Developing services to fit the local context, e.g. the development of remote memory clinics for rural areas [24]^(M)^, may help to encourage earlier help-seeking.

### Post-diagnostic support for people with dementia

We found limited high quality studies of post-diagnostic support interventions, possibly because the drive to provide post-diagnostic support is too recent to have allowed for completion and publication of anything other than small scale qualitative studies. However, those reviewed indicate a variety of experiences of post-diagnostic support, both in accessibility and focus. For example, research has suggested that people with frontotemporal dementia have difficulty finding and engaging appropriate home and community based services due to lack of understanding and knowledge of frontotemporal dementia [25].

Non-pharmacological interventions are increasingly used as alternatives to medications and our review indicates the variety of interventions being tested, albeit with mixed results. For example Lauriks et al. [26] proposed that people with mild to moderate dementia can benefit from information and communications technology (ICT) solutions aimed at compensating for disability, while Kurz et al. [27]^(M)^ found that cognition-focused interventions confer small and inconsistent effects on general cognitive ability.

### Post-diagnostic support for family caregivers

It is important to provide support for both people with dementia and also for those who care for them. UK-based research which found a relationship between carer anxiety and depression and family carers' abusive behaviour to people with dementia [28]^(M)^ highlights the need to develop evidence-based interventions directed at reducing carer burden and supporting carers to develop appropriate coping strategies.

Supporting caregivers is at the heart of government policy in Scotland [8,9,21] and the UK [20] and is reflected in attempts to develop relevant, community-based interventions. Our review highlights a similar focus on supporting caregivers at an international level, from a homecare programme using locally available resources to support caregivers of people with dementia in Goa, India [29] to a German intervention providing assisted vacations for men with dementia and their caregiving spouses [30]^(M)^. International research also indicates that multiple component interventions may assist in supporting caregivers of people with dementia living in the community [31] and that multimodal interventions are associated with decreases in caregiver burden [32]^(M)^.

### Post-diagnostic support of paid carers

Our review indicates there is limited research exploring the needs or experiences of paid carers who support people with dementia and their families to live at home. What literature there is focuses on bio-medical support or support needs. For example Forsetlund et al. [33]^(M)^ concluded that Norwegian doctors, pharmacists and nurses do not receive much training in drug treatments for older people, recommending educational outreach to reduce inappropriate drug use. In the UK, Cross et al. [34]^(M)^ found that Community Psychiatric Nurses (CPNs) consider delivery of memory rehabilitation strategies part of their role, but report limited knowledge of potential memory strategies and aids.

### Community-based services supporting people with dementia living in their own homes

This section provides insight into the context in which community-based dementia care takes place and examines the evidence regarding how best to provide a nurturing and supportive environment for care at home. Seven areas of services were considered under this heading, as set out below.

### Self-directed care and support

‘Self-directed support’ (known elsewhere as individual budgets or direct payments) has been promoted by UK devolved Governments [35-37] as increasing choice and control for all service users, on the basis that people receive funds and are facilitated to spend them on their own priorities for support rather than standard services identified by a care manager. However, no recent evidence or information was identified concerning the uptake of self-directed support by people with dementia or their carers, suggesting this may be a research gap.

People with dementia can also influence the support that they receive and the way it is delivered through ‘advance directives’ or ‘advance decisions’ which set out preferences for future care in the event that they lack capacity to make decisions when treatment is required. Sampson et al. [38]^(M)^ found carers reluctant to write advance care plans, highlighting the importance of ensuring that people with dementia are aware of and supported to make advance decisions as early as possible in their journey with dementia.

### Rapid response

This refers to the use of multidisciplinary teams who can attend people in crisis at home, providing support that enables them to remain there. Such services have only recently been introduced in the field of dementia care and the evidence-base is currently sparse. While mention is made of rapid response interventions, for example where discussed in the context of an evaluation redevelopment program in a mental health service for older people [39]^(M)^ and elsewhere as part of a nurse led pilot of an integrated care programme for older people with dementia [40], we were unable to identify research that took this as a focus.

### Day services

Day care is a more established area of provision for people with dementia and we were consequently surprised to find only limited recent research. The identified studies explored reasons for refusing to attend or for leaving day services and questioned the value of such services.

Durand et al. [41]^(M)^ found evidence of substantial numbers of people with dementia who lived alone refusing day care opportunities, with the most common reasons given involving individuals’ perceptions of need for and/or enjoyment of day services, coupled with concerns about meeting new people, losing independence and being institutionalised. The authors suggested that more than half of those declining day care might be suffering from undiagnosed depression.

A Swedish study [42] found that where day services were taken up, one third of people with dementia dropped out within four months with a further third dropping out within 12 months. Study data suggested that behavioural disturbance and high levels of physical care needs were predictors for short-term uptake of day services, leading the authors to suggest that offering day care to people with behaviour disturbances may be of questionable value.

Mason et al.’s [43] systematic review also questioned the value of day care, concluding that ‘No reliable evidence was found that respite either benefits or adversely affects care recipients, or that it delays entry to residential care’ (p.77), but that according to included comparative economic analyses day care can cost more than other care arrangements. However, both Mavall and Malmberg [42] and Mason et al. [43] found that caregivers perceived benefits of day care for their relatives and for themselves, the latter concluding that respite ‘may have a small positive effect’ on carers’ burden and mental or physical health (p.xii).

### Managing medication

The review identified limited research in this area. Arlt et al. [44]^(M)^ identify as a key issue how best to support people with dementia to adhere to a medication regime and examine the challenges presented by the need for on-going monitoring and adaptation of arrangements over time, highlighting questions of when to transfer responsibility away from the person with dementia and how this is done.

Jedenius et al. [45]^(M)^ recommend that that management of medication should be integral to dementia care services. Their retrospective drug use study found that the introduction of a multi-disciplinary dementia management programme including the optimisation of pharmacological treatment had led to reductions in prescription of psychotropic medications and increases in anti-depressant and anti-dementia medications.

### Community-based services supporting people with dementia living in their own homes

This broader heading encompasses support available at community level such as information provision, support for carers, and residential respite care facilities. A number of papers covered contextualising considerations for the provision of community-based support: drawing attention to the risk-averse culture behind much service provision and its implications for the person with dementia [46]; questioning the criteria used to evaluate provision and their failure to attribute greater importance to supporting identity [47] and maintaining personhood [48] of the person with dementia; and arguing for the application of a recovery model as a framework for the provision of care and support [49,50]^(M),(M)^.

Mason et al.’s [43] 2007 systematic review noted very limited comparative evidence on the relative benefits of different models of community-based support and called for rigorously evaluated pilot models using standardised criteria for assessment and comparison. As previously noted, they also found little or no significant evidence that respite care benefits people with dementia, delays their transition to residential care, or is more cost-effective than other forms of support. However, Wilz and Fink-Heitz’s [30]^(M)^ evaluation of an assisted vacation intervention for people with dementia published a year later found that it had both immediate and lasting benefits for the person with dementia and their carer.

Accessible information can support people to better understand local provision and make timely decisions about accessing services. However, Ploeg et al. [51] suggest that people often do not know where to turn for information on services, relying mainly upon their General Practitioner (GP) or primary care physician.

Several studies and reviews identify a lack of current evidence in relation to more recent innovations in community-based support [26,52,53]^(M),(M)^. One key area where further research is required is the use of technology in the context of living with dementia. Preschl et al. [52]^(M)^ argue that more research is needed into the benefits of e-health technology in the context of supporting people with dementia, while Lauriks et al. [26] suggest that further research might help us to better understand the role of information and communication technology in addressing unmet need as perceived by people with dementia and their carers. Buettner et al. [53]^(M)^ assert that despite the current limited evidence base, technology-based interventions hold promise for improving safety at home, reducing carer burden and reducing the overall costs of home-based dementia care. Carswell et al. [54]^(M)^ suggest that many forms of assistive technology could be adapted for use in the context of night-time care, an oft-neglected subject in the research literature, and thereby contribute to supporting people to remain at home.

### Domiciliary support

In contrast to findings that home care is often task-oriented and time-limited with care workers under pressure to complete their work against the clock, Rothera et al. [55] found that the most effective forms of home care with the best outcomes are flexible in their design and responsive in their delivery.

Domiciliary support has an important role to play in the transition from hospital to home and increasingly in end of life care. Sampson et al. [56] show that transitions to hospital at this stage are detrimental for both the person and the carer, particularly for people with more severe dementia. They recommend that people with dementia should be supported to remain in a familiar environment. However, for this to be feasible there is a need for advance planning, a care pathway for people who wish to remain at home, and better training on end of life issues for community-based support services.

At present, evidence suggests that palliative care is not optimal, often because of a failure to recognise dementia as a terminal condition [38,57]^(M),(M)^. Differences in level and type of support have also been found according to whether or not there is a formal diagnosis of dementia. Above all, research points to the importance of fully involving carers in end of life care for the most positive outcomes [58,59]^(M)^. Routes to improving current provision include the development of more appropriate forms of assessment, the need for more tailored support and the effective co-ordination of services [38,57,60]^(M),(M),(M)^.

### Enablement, re-ablement and rehabilitation

Recent studies have raised questions over the aims and outcomes prioritised by much of this work: past studies have often focused on biomedical aspects such as cognition, functioning, mood, behaviour etc. – with far less attention on outcomes defined by and/or important to people with dementia. A key consideration here is continuity, which has been rated highly alongside support that enables people to maintain their normal lives.

There is a growing body of evidence concerning non-pharmacological interventions (e.g. [61]), reflecting the need to respond to evidence of widespread over-prescription of psychotropic medicines for people with dementia. Cognitive interventions have been shown to be beneficial to people with mild cognitive impairment, impacting positively on language skills, communication and other activities of daily living (e.g. [62,63]). Recent years have witnessed increasing interest in the potential benefits of different forms of cognitive rehabilitation but many reviews conclude that there is a need for further evidence (e.g. [27,64]^(M),(M)^). Ballard et al. [65]^(M)^ found modest but significant evidence of benefits for different types of cognitive intervention, with evidence strongest for cognitive stimulation therapy, but also call for further research. Faucounau et al. [66]^(M)^ highlight the benefits of computer-based cognitive interventions, noting the potential for tailoring to individual needs. Cotelli et al. [67,68]^(M),(M)^ also draw attention to cognitive rehabilitation, including the use of reminiscence therapy, noting positive implications for rate of decline in cognitive functioning, albeit based upon a small evidence base.

There is emerging evidence of the benefits of outdoor and physical activity interventions [69]^(M)^, including horticulture therapy [70], as part of a broader public health remit for the support of people with dementia, although at present there is need for more rigorous evaluation of interventions. Supporting nutrition through joint working by services for monitoring and support also has value, especially in the care of people with advanced dementia [71]. The potential role of appropriate indoor and outdoor design in enabling people with dementia to remain independent has also been highlighted, with calls for further research into the meaning and benefits of ‘smart homes’ [72]^(M)^ and into design innovation as a means of supporting people living at home [73]^(M)^.

### Hospital-related areas of interest

The review focused on transitions to or from hospitals and in particular on services which either prevented admissions or facilitated faster healthier returns home after necessary stays. Themes addressed within this area included: intermediate care; preventing unnecessary admissions; use of community hospitals; and reduction in length of stay and discharge.

### Intermediate care

This sub-area focuses on short-term services to support people to return home following hospital stays. Such services are known variously as intermediate care [74] or transitional care [75] programmes. None of the included texts had this type of service as a primary focus: publications identified by this review tended to refer to intermediate care in passing, e.g. in the context of reviewing service provision more generally [75], in examining carers’ roles in hospital discharge [74], in the course of discussion of decision-making capacity [76], or as a component of integrated care programmes [77] ^(M)^. We found no direct research evidence relating to intermediate care services.

### Preventing unnecessary hospital admission

Strategies suggested for the prevention of unnecessary hospital admissions include: adapting living environments to reflect emerging needs in physical, sensory and behavioural impairments [78]; increasing participation in activities that prevent/delay dementia onset [79]; and offering combined interventions for both caregivers and those they care for [80]. Non-pharmacological interventions have been shown to be more cost-effective than technological interventions or medication in allowing people with dementia to be cared for at home for longer [61] and to have additional beneficial consequences for carers [81]. However, services need to be coordinated, particularly those designed to improve end-of-life care [82] and to develop advanced care planning for later palliative care [56]. Jones [40] identifies the need to monitor the emerging needs of people diagnosed with dementia who may not need immediate care and suggests the use of an ‘adaptive rehabilitation’ model of care to provide high quality care in the community.

### Community hospitals

People with dementia treated in general community facilities may receive poorer care. One study [59] reported that people with a formal diagnosis of dementia received different end-of-life care for their final hospital stay compared with those without diagnoses of dementia when admitted. Those diagnosed with dementia had restricted access to palliative care and their caregivers were consulted less often about treatment decisions. However, the provision of dedicated community facilities can lead to improved services. Awata [83] noted that a Japanese model for a Special Medical Consultation Room (SMCR) improved local medical care for people with dementia, as reflected in higher rates of differential diagnosis on the first visit, increased admittance to psychiatric wards and decreased waiting times for clinical consultations with doctors.

### Reductions in length of stay

This sub-topic focuses on the identification of practices and strategies to minimise the time that people with dementia spend in hospital as a result of factors not directly related to their reason for admission, whilst acknowledging that shorter hospital stays will not be a desirable goal in all circumstances. Direct evidence was limited. Amella et al. [71] concluded that a team approach and inclusion of all people involved in the care process for persons with moderate and late-stage dementia resulted in better communication, shared knowledge and understanding of how best to treat (nutrition) issues, without which length of stay might increase. An examination of the discharge planning process by the Association of Directors of Adult Social Services of England (ADASS) [74] revealed gaps in post-discharge preparation and drew attention to the sparseness of opportunities in acute settings to review practice and improve outcomes.

### Informal carers

Consideration of support for informal carers is essential, given their critical role in delivering support for people with dementia, especially at home. Both policy and practice are predicated on assumptions that the informal carer workforce is large, and both benefit from its effectiveness: thus services supporting its effectiveness need to find the best ways of working well.

Carers’ issues are addressed in a range of research, and we have already noted some of these under other headings. Here, our focus is on identifying ‘big issues’ that cross other domains, and on highlighting conclusions that emerge from research specifically focused on carers. We should note that much of the literature assumes the presence of informal support with little discussion of people without access to informal carers: this is a significant gap in the research.

Support for carers is a strong theme in the literature. Chien et al.’s [84] meta-analysis suggests that evidence for the benefits of support groups is strong, especially for psychological well-being and depression (but less so for ‘burden’). Coping strategy based support is especially helpful, as ‘dysfunctional coping’ predicts depression and anxiety in carers [85-87]. Cooper et al. [87] argue that improved coping strategies can improve quality of life for those receiving care. Dysfunctional coping is said to include behavioural disengagement, denial, self-distraction, self-blame, substance use and venting, and it is suggested that it can lead to abusive care [88]^(M)^.

A further promising area of support is training or education for carers [89-91]^(M),(M)^. Galik et al. [89] suggest that this can support carers to maintain engagement and activity of people with dementia, i.e. ‘restorative care’, providing ideas about possible means of doing so. Harland et al. [90]^(M)^ recommend a user-centred approach on the basis that information can increase as well as reduce problems for carers, findings confirmed by Corbett et al.’s [91]^(M)^ systematic review.

The literature identifies some limitations in carer support interventions. Moniz-Cook et al. [92] suggest that interventions designed to support carers could usefully include functional analysis (exploring reasons for ‘challenging’ behaviours) but find little evidence of efficacy for its sole use. Mason et al. [43] in another systematic review find that respite care has ‘modest effects’ in improvements in carers’ physical and mental health, but not that of people with dementia, adding that there is no evidence that it delays admission to institutional care. Very little research has been conducted on the needs of black and minority ethnic carers, and service providers have made little attempt to engage with minority communities [23]. In relation to end of life care, carers of people with dementia are consulted less than carers of people who do not have dementia [59] despite evidence that admission to hospital for end of life care is particularly detrimental for people with dementia and their carers [56].

### Strategies for carers at home

A range of possible strategies that carers might use supporting a person with dementia at home is reviewed. These include adopting a holistic perspective on ‘nutrition difficulties’, focusing on social, cultural and environmental factors and providing tips on managing mealtimes at home [93]. ICT use can enhance positive affect and feelings of safety [26] and can be helpful at night as company, prompting or presence [54]^(M)^. ICT can also be used to ascertain people’s views about services [94]^(M)^, and it has been argued that telehealthcare can support good practice and achieve value for money [95]^(M)^. Others identify potential beneficial effects of medication, with one review finding evidence that cholinesterase inhibitors can decrease ‘carer burden’ [96]^(M)^. According to Wilz et al. [2,30]^(M)^, assisted vacations may have long lasting positive effects.

Smits et al. [80] and Parker et al. [31] emphasise the importance of ‘combined intervention programmes’ or ‘multi-component interventions’ that can delay entry to institutional care, not least because these have stronger impact on mental health. In their view, single strand interventions (such as carer support groups) are less effective than multi-stranded interventions [31,80]. Rothera et al.’s [55] work complements this by emphasising that flexible and individualised care at home is better than task-focused care. Ways to achieve this might include ways of managing risk that do not excessively constrain, and that involve carers [46].

There are some cautions in the literature concerning strategies to use at home. For example, Damianakis et al. [97]^(M)^ found that participatory development of multimedia biographies stimulated memories and enhanced social stimulation, but involved huge time investment of participants, including researchers. They concluded that it was probably not cost effective, and warn against over-complex and intensive interventions. A second caution relates to neglected areas which can nevertheless be fundamental: for example, incontinence can often be the trigger for admission to institutional care, but one systematic review [98]^(M)^ questions whether carers get sufficient support with this sensitive and difficult issue.

### Relationships at home

Services need to understand the relationships in which people with dementia are embedded and consider these in service provision [99]. A meta-analysis demonstrated that involvement and choice in services for people with dementia and their carers differentiate effective interventions from ineffective ones [81]. Relevant examples include the need for carers to be involved in hospital discharge processes for better outcomes [58,74] and the widely reported fact that family members may be the first to notice changes caused by dementia. Villars et al. [100] ^(M)^ question whether professionals are listening to them.

### Workforce and service delivery

Workforce and service delivery issues cross-cut all the other domains and are similarly fundamental to service delivery: an effective, supported workforce, which can operate in partnership with informal carers is clearly essential to care at home, and is not necessarily readily sustained. Furthermore, services are increasingly aspiring to engage in multi-professional working, delivering integrated care through integrated teams.

### Joint/partnership working and integrated teams

The literature supports the use of an integrated multidisciplinary approach when dealing with complex multifactorial dementia-related issues, such as co-morbidities [101], eating and nutrition [71], and palliative care [102]. Joint working can promote service improvement and raised standards when using multidisciplinary, integrated approaches in areas such as palliative care [102], multi-component interventions for carers [81], outreach services [103], and specialist adaptive rehabilitation services [48]. Joint working can promote more holistic service provision, not least because it can help to highlight contradictions in care and practice intentions [46]. Joint working and multidisciplinary, integrated approaches can also benefit professionals by facilitating access to knowledge and collaborative learning [40,46]. Brief interdisciplinary educational interventions may lead to more positive attitudes and greater effectiveness when working in interdisciplinary health care teams [104] ^(M)^.

However, a number of issues emerge from the review. Some commentators identify problems with the quality of evidence, in terms of research design, study size, research setting, intervention specification and outcome measurement, all of which are frequently limited [101,102].

In some cases, such approaches are sub-optimal due to inadequate/ineffective communication, organisational/disciplinary boundaries which inhibit effective working, and issues of co-ordination [71,105]. Furthermore, establishing and sustaining joint working and/or multidisciplinary approaches can be challenging, and there is a need for more specific and appropriate commissioning [48,103]. A further challenge is that integrated care tends to increase service use but does not necessarily improve clinical outcomes [77] ^(M)^.

### Consistency and quality of home care (staff training and support)

Our review revealed no high quality findings relating to home care staff training and support. It emerged that home care staff training and support may be under-researched areas: although relevant topics have been researched in care home settings, e.g. in Aselage and Amella’s [93] study of mealtimes, there are questions around generalisability of findings to home care contexts.

### Community nursing

Similarly, our review did not identify any high quality work directly relating to the role of community nursing in dementia services and care: references tended to relate to CPNs as part of specialist teams [48]. One study did suggest that a nurse-led psychiatric consultation service model functioned well in comparison with traditional medically-led consultation models and could lead to cost savings [106] ^(M)^.

### Community based support (workforce issues)

Carer experiences are affected by the presence or absence of dedicated workers for carers or community support services for carers in different contexts, e.g. around hospital discharge [74]. However, evidence suggests that many professionals and paraprofessionals do not receive adequate training in key aspects of dementia care [107,108], including for example how to give culturally acceptable care and support to BME people with dementia [23] and end of life issues [59].

Evidence also supports the view that in addition to adequacy of training, professional carers’ approaches to risk management may impact on the well-being of individuals. The extent to which physical risk is privileged (to the detriment sometimes of psychological and emotional well-being) and the ways in which information is communicated within and between services have implications for people with dementia being supported in the community [46].

### Day services (workforce issues)

Although workforce issues were mentioned as noted above, our review identified no studies which discussed workforce issues in the context of day services

## Discussion

### Emerging issues

The findings of this review suggest variable experiences of diagnosis for different groups, highlighting the importance of recognising and working to address diversity of experience and need. In relation to post diagnostic support, the literature suggests that locally-based, multi-component interventions including education, cognitive stimulation, cognitive training and cognitive rehabilitation may be useful to support family carers to support people with dementia to live at home. The literature also highlights knowledge gaps of key practitioners and under-used potential of Community Psychiatric Nurses (CPNs). This review considers literature published up to late 2012. It would be interesting to know to what extent these latter points remain true, given the elevation of dementia in policy agendas in the UK and elsewhere [6,7].

Overall, the evidence on community-based services supporting people with dementia living in their own homes is limited and there is a clear need for more UK-specific research. Hence we need to remain cautious about recommendations. A striking feature of this part of the review was the number of headings for which we found limited supporting research and in some cases a total lack of UK-based research evidence. However, newly emerging concerns for the field of dementia care and support have been identified by the review, e.g. take-up and use of self-directed support, use of multidisciplinary rapid response teams, use of technology to support community-dwelling people with dementia. These topics offering something of a roadmap for future research on community-based support to people with dementia.

Literature examined as part of this review suggests that the best outcomes for people with dementia are associated with services that are timely, responsive, flexible and tailored to individual need. However, community-based support to people with dementia is a rapidly changing landscape, with implications for areas of knowledge required. For instance, the shift from hospital-based and institutionalised forms of care to support embedded in the community is well underway but in areas such as preventative services, which are seen as a potentially cost effective model of support in the community context, research has failed to keep pace with these changes. As criteria for access to services tighten and more specialist services target complex needs, the role of the not-for-profit sector in the support of people with dementia is likely to develop, accompanied by greater emphasis on practitioners working collaboratively with informal support networks in which people with dementia are embedded. As people are supported to remain in the community for longer there will be growing pressure on services to incorporate changing needs over time into service design, to understand changes from the perspectives of people with dementia and carers, and to promote more co-productive ways of working.

Many authors highlighted the need for additional research in hospital-related areas of interest, for example to explore and identify what is most beneficial in preventing and/or delaying the onset of dementia [85], to develop and validate tools measuring subjective quality of life for those with restricted abilities to communicate [76], and to develop and test more effective approaches to end-of-life care [59]. Some commentators [71] have called for increased use of approaches such as case management to improve outcomes in dementia care. However, Koch and Iliffe’s [109] review identified no UK-based empirical studies of this approach, and US-based studies have found that whilst case management approaches to support have led to increased levels of service user satisfaction, they show little improvement to clinical outcomes [77]^(M)^, and the cost benefits of such approaches are unclear [110]^(M)^.

In relation to issues around unpaid or informal care, included items reaffirm the fundamental importance of unpaid carers and vital need for them to be supported to continue their work, highlighting the need for their involvement with services in joint delivery of support.

Neglected issues in this area include end of life and continence care: both highly sensitive and difficult for carers to address at home. In addition, there is the important issue of identifying and supporting people who do not have informal care: there is a relative paucity of research in this area and policy cannot and should not assume that carers are present, or that people have support from their own networks [111]. One study emphasised importance of ascertaining their views in connection with refusal of day care [41]^(M)^: this had not been seen as important.

The literature suggests that many one-off interventions can show local and limited positive effects, but the evidence that multi-component approaches are more likely to be successful is compelling. A key unanswered question is whether these ‘one-offs’ are successful because they provide vehicles for engagement, rather than because of their actual content.

### Cross-cutting themes

In the course of this review we have identified themes and issues that related to most or all of the areas considered. For instance, despite an emerging contribution to dementia studies from the humanities and social sciences we found that much of the literature adheres to a bio-medical model of dementia, characterised by a focus upon symptoms and their management. There were however signs of change. In particular, we found growing recognition in both study design and recommendations of the importance of involving people with dementia and carers in research, policy and service delivery.

A more recently emerging theme concerns recognition of the diversity of people with dementia. For instance, there is growing research evidence around the specific needs and challenges faced by people with dementia in remote and rural communities [24]^(M)^, and of the different experiences of people with different forms of dementia (e.g. [25]) or people from black, Asian and minority ethnic communities [23] of accessing services and support.

### Implications for practice

Demographic change, improved understandings of the prevalence of dementia and changing economic and policy landscapes are all acting as drivers for service innovation in countries around the world, strongly influencing both the pace and extent of change. Weaknesses in the evidence base present challenges both to practitioners looking for guidance on how best to design and deliver evidence-based services to support people living with dementia in the community and their carers and to those charged with inspecting such services.

### Limitations

As with all systematically conducted literature reviews, the formulation of research questions, selection of search terms and sources to be searched and inclusion criteria employed can all be considered as limitations to the study. In the present study, the broad range of areas under consideration contributed to the identification of a large volume of potentially relevant publications and necessitated the development and application of additional criteria to manage the process of item selection. As a result, the texts included in the review represent the breadth but not necessarily the depth of the evidence base in all areas. Prioritizing the inclusion of literature reviews was intended to counteract this issue, but may have resulted in unhelpful generalisation and abstractness.

We would nonetheless suggest that the review reveals that the quality and extent of the evidence base for what works in care at home for people with dementia remains limited. High quality evidence is sparse, irrespective of the research design or methodological approach taken. We have included and evaluated studies from a wide range of research approaches, finding the literature for the most part suggestive as to what works, and must conclude that policy and practice developments are proceeding on a limited evidence base.

## Conclusions

Key issues with the existing evidence base include: both variability and uncertainty in outcome measurement, in particular a noticeable dearth of focus on the perspectives of people with dementia themselves and their informal carers and supporters; frequent failure to demonstrate effective understandings of the complexities of living with dementia, and of the kinds of multifactorial interventions that are needed to provide holistic and effective support; and poor research design coupled with tendencies to focus on only one element of support provision.

This review was commissioned to support an inspection regime, but it is equally important that service commissioners, service providers and those researching this area: are able to appreciate the limitations of existing evidence; seek to review local evidence that approaches really work; understand and act on what evidence is available; and respond to service users, engage with them, and involve informal carers.

## Additional files

Additional file 1: **Supplementary document Care Inspectorate Paper BMC Geriatrics 8May2014.docx.** This file comprises a table providing information on the publications included in the literature review. The table lists publications in alphabetical order by first author and provides: author(s); year of publication; title of publication; abstract (or summary where no abstract was provided); study type; country in which research took place; and evaluated quality of publication (high, medium or low). The file is in Word docx format.
Additional file 2: **Dawson et al. Evidence of what works PRISMA 2009 Checklist 9Mar2015.** This file is a PRISMA 2009 Checklist for the current study. PRISMA stands for Preferred Reporting Items for Systematic Reviews and Meta-Analyses. It is an evidence-based minimum set of items for reporting in systematic reviews and meta-analyses. The checklist provides an indication of where items expected to be in a systematic literature review can be found in the text of the main paper.
