# Supplementary material for: Evidence of what works to support and sustain care at home for people with dementia: a literature review with a systematic approach
Source: BMC Geriatr. 2015 May 13;15:59. doi: 10.1186/s12877-015-0053-9 (PMC4465454; doi:10.1186/s12877-015-0053-9)
Supplement: Additional file 1: — Supplementary document Care Inspectorate Paper BMC Geriatrics 8May2014.docx. This file comprises a table providing information on the publications included in the literature review. The table lists publications in alphabetical order by first author and provides: author(s); year of publication; title of publication; abstract (or summary where no abstract was provided); study type; country in which research took place; and evaluated quality of publication (high, medium or low). The file is in Word docx format. [file 12877_2015_53_MOESM1_ESM.docx]

# Supplementary document for ‘Evidence of what works to support and sustain care at home for people with dementia: a literature review’, Alison Bowes, Alison Dawson, Fiona Kelly, Kari Velzke and Richard Ward

Table of included references and their study types, country publication relates to, and quality assessments (in alphabetical order by first author)

| **Author(s)** | **Year** | **Title** | **Abstract or brief summary of publication where no published abstract available** | **Study type as noted in main paper; Country publication relates to; Evaluated Quality** |
| --- | --- | --- | --- | --- |
| Ablitt A, Jones GV and Muers J | 2009 | Living with dementia: A systematic review of the influence of relationship factors | Introduction: Many people with dementia are enabled to live at home by the support of a close family member, who takes on the role of a carer. Considerable research has investigated the impact of caring for a person who has dementia. In early research, there was a tendency to overlook the experiences of the person with dementia and, in particular, the relationship between the two persons. This has now been corrected by a growing body of research on the relationships between people with dementia and the family members who care for them. Method: Peer-reviewed publications on the influence of relationship factors in dementia care giving were reviewed. Results: The impact of dementia on the quality of relationships is examined, together with the impact of relationship quality on the experience of living with dementia. The different forms that relationships can take in the context of dementia are considered, and an integrative theoretical framework is proposed. Discussion: A neglect of direct evidence from the person with dementia is identified, and possible ways of combating this are considered. Clinical implications are drawn with regard to supporting the carer, the person with dementia, and their relationship. | Literature review; International; HIGH |
| Adams T | 2010 | The applicability of a recovery approach to nursing people with dementia | OBJECTIVES: Recent developments in nursing to people with mental health conditions of working age have been underpinned by the recovery approach. This paper critically reviews the idea of recovery in relationship to people with dementia and examines its applicability to dementia care nursing. DESIGN: The paper critically reviews literature relating to the use recovery approach and the people with dementia, particularly their nursing care. The paper identifies common ideas within two approaches and suggests how the recovery approach may underpin nursing care to people with dementia. DATA SOURCES: A search of CINAHL, Medline and PsycINFO was undertaken from 1987 onwards using keywords 'recovery', 'nursing' and 'dementia'. RESULTS: The paper found that the recovery approach shares many ideas with person-centred approaches to dementia care and illustrates this in relationship to well-being, social inclusion, self-management, and hope. CONCLUSION: The paper concludes by suggesting that dementia care nursing should draw on ideas taken from the recovery approach and identifies each approach drawing on ideas that have come together in postpsychiatry. | Literature review; International; MEDIUM |
| Afzal N, Buhagiar K, Flood M and Cosgrave M | 2010 | Quality of end-of-life care for dementia patients during acute hospital admission: A retrospective study in Ireland | Objectives: To examine the quality of end-of-life care received by patients with and without dementia on acute medical wards during their final hospitalization. Methods: A retrospective clinical case note review of patients aged over 65 who had died on acute medical wards within a 6-month period in a general hospital in Dublin was conducted. Seventy-five multidisciplinary clinical notes were available for scrutiny in order to identify cognitive status, measure the frequency of invasive procedures undertaken and examine the quality of palliative care as benchmarked with the Liverpool Care Pathway for the Dying Patient (LCP) program. Comparison between patients with and without dementia was made. Results: Eighteen (24.0%) subjects had dementia, 32 (42.7%) subjects were described as "cognitively intact" and 25 subjects did not have reference to cognitive status. Of the 50 patients with known cognitive status, 27 (54.0%) had had a Mini Mental State Examination (MMSE) conducted (10 dementia vs. 17 nondementia). Patients were equally subjected to invasive interventions regardless of their cognitive status. However, dementia patients were significantly less likely to be referred to palliative care interventions (P=.007), to be prescribed palliative drugs (P=.017) and to have carers involved in decision making (P=.006). Conclusion: Individuals with dementia may be receiving different end-of-life care from those without. The effective delivery of robust multidisciplinary frameworks for the palliation of symptoms of hospitalized dementia patients remains an important clinical goal. | Other (Retrospective case review); Ireland; HIGH |
| Ahmad S | 2009 | Improving services and support for people with dementia: Using a web forum to capture the views of people with dementia and their carers to inform a national audit office report | *(No abstract on original paper – summary provided by current authors)*. This short paper describes the use of a web forum to capture the views of people with dementia and their carers to inform a 2007 national audit office (NAO) report entitled ‘Improving Services and Support for People with Dementia’. The forum comprised a web-based survey accessed from the NAO website which included questions on people with dementia and/or their carers’ needs for, access to, and experience of dementia services. The survey was made available in alternative formats and through other channels. 174 responses were received. Findings and quotes from the survey were used in the report. | Other (Report of survey); UK; MEDIUM |
| Amella JA, Grant AP and Mulloy C | 2008 | Eating behavior in persons with moderate to late-stage dementia: Assessment and interventions | Persons with various forms of dementia suffer from a progressive disease in which memory and the ability to function independently are lost. During moderate to late-stage dementia, individuals experience increased difficulty with eating and require more feeding assistance. Clinicians working with this population must acknowledge the multifactorial aspects of eating behavioral issues, use a team approach, and make careful assessments using appropriate instruments. Interventions should include attention to cognitive impairment, nutritional intake, training of caregivers, modification of the environment, and the quality of the interaction. Planning for care should include promoting the highest quality of life for these individuals and their caregivers. | Literature review; USA; HIGH |
| Anderson D, Cattell H and Bentley E | 2008 | Nurse-led liaison psychiatry service for older adults: service evaluation | Aims and Method: To comprehensively describe a nurse-led consultation liaison service for older adults by retrospectively reviewing all referrals received in 2006 and comparing them against other services and benchmark reports. Results: Of the 298 individuals referred to psychiatric services from other hospital wards, 120 were aged 85-94 years old (40%), 193 were male (65%) and 152 were referred from geriatrics (51%). A majority of 204 have not had previous contact with psychiatric services (69%).The most common diagnosis was dementia (33%, n = 88), with 27% individuals (n = 65) being referred onwards to secondary care. Clinical Implications: This nurse-led service, using a novel approach of a support worker providing further community support, functions well compared with traditional consultation models. It helps identify many individuals with dementia and engages them into community psychiatric services. | Other (Service evaluation); UK; MEDIUM |
| Arlt S, Lindner R, Rosler A and von Renteln-Kruse W | 2008 | Adherence to Medication in Patients with Dementia- Predictors and Strategies for Improvement | Measures to facilitate patient medication adherence should be considered an integral part of the comprehensive care of older patients with multiple diseases. However, impairment of cognitive functions and dementia, in particular, may substantially compromise adherence behaviour. Therefore. a literature review was performed to identify factors associated with adherence to medication in patients with cognitive impairment or dementia, and to discuss strategies for improvement of non-adherence. Evidence-based information on how to deal with adherence to medication in patients with dementia is scarce because of a lack of specific studies. However, there is increasing knowledge about factors influencing medication adherence behaviour in older age, and emerging insight into the relationships between adherence behaviour and cognitive capacity, memory and executive function, in particular. Nevertheless, understanding elderly persons' strategies for maintaining regular use of even complex drug regimens is still limited. Progress of research in this field is needed. It is notable that measures to improve adherence consist of combinations of educational interventions and cognitive support but assessment of study participants' cognitive function is rare. In clinical practice, awareness of non-adherence as a result of cognitive impairment is relatively low. The most important step is early detection of cognitive impairment when this is impacting negatively on medication management. A practical geriatric screening test is recommended to identify memory problems and further functional impairments associated with cognitive impairment. Performance-based assessments might be useful for screening medication management capacity, in addition to a careful drug history, inspection of all medicines used (including over-the-counter drugs) and proxy information. However, no feasible screening methods have as yet found their way into clinical practice. Patients with impaired executive function, lack of awareness of illness and personality traits such as independency and high self-confidence may be at particular risk of non-adherence. The question is when to switch patient medication self-management to another person's responsibility if cognitive decline progresses. Further research is needed on measures to differentiate cognitive function and the relationships between memory concerns, memory strategy use and medication management. Also, studies evaluating the influence of personal support, health status and depression on the memory strategies used are needed. It is important to assess patients' attitudes toward medication and their relationship with proxies. Strategies for facilitating medication adherence in patients with dementia include prescribing as few medicines as possible, tailoring dose regimens to personal habits, and coordinating all drug dosing schedules as much as possible. When providing medication organizers. it is important to observe the patient's ability to use devices appropriately. In addition, automated computer-based reminding aids, online medication monitoring and telemonitoring may be helpful for patients with mild dementia. The decision as to when assistance with medication self-management is needed has to be made taking into account patient independency and safety aspects. This holds true for medicines with a narrow therapeutic range, in particular. Interactions among the individual patient's cognitive status, mood, level of self-efficacy and particular living situation must also be taken into consideration when searching for the optimal medication adherence strategy. No evidence-based recommendations can be given as yet. However, comprehensive assessment of the individual patient and careful consideration of all potential drug-related problems will probably help facilitate adherence and prevent compromised health Outcomes in patients with dementia. | Literature review; International; MEDIUM |
| Aselage MB and Amella EJ | 2010 | An evolutionary analysis of mealtime difficulties in older adults with dementia | AIMS AND OBJECTIVES: To use the evolutionary method of concept analysis to identify attributes, antecedents and consequences of mealtime difficulties providing direction for assessment and management in older adults with dementia.; BACKGROUND: Mealtimes encompass more than the physical act of feeding a person with dementia. Social and contextual considerations are vital considerations to improving nutritional intake. While feeding difficulties in dementia have been analysed in the literature, this paper proposes a broader scope of mealtime considerations to alleviate nutritional deficiencies often associated with dementia.; DESIGN: Evolutionary method of concept analysis. METHODS: In 2008, literature searches using keywords (meal, history, sociology, mealtime, culture, habit, dementia, dementia) were done in CINAHL, Academic Search Premiere, MasterFILE, Americal Life and History, Communication and Mass Media Complete, EJS, Health Source Plus-Academic, PsychARTICLES and PsychINFO, ScienceDirect, Sociological Abstracts and Google. Year limits were from 1988-2008. A total of 659 abstracts were reviewed, Google, books and textbooks with relevant content. RESULTS: Forty-eight sources were used in the final analysis of mealtime difficulties in older adults with dementia. A model of mealtime difficulties delineates attributes, antecedents and consequences. CONCLUSIONS: Mealtime difficulties in dementia emerged as a concept with supporting evidence-based practice guidelines in 2003. Most research has been conducted in institutional settings, but community research is growing as the shifting demographics of ageing demand attention for this setting. Interventions vary in effectiveness for alleviating sequelae of mealtime difficulties in older adults with dementia. RELEVANCE TO CLINICAL PRACTICE: The conceptual model of mealtime difficulties provides a broader scope of mealtime difficulties in dementia that considers environmental, social, cultural and contextual implications with nutritional intake. The model can be used to guide future research to alleviate mealtime difficulties in older adults with dementia. | Other (Literature-based concept analysis); USA; HIGH |
| Ashton S | 2011 | Using compliments to measure quality | A project in which nurses on a dementia assessment ward designed, implemented and evaluated a way of collecting compliments to measure performance is described. The project helped the team to understand their performance in terms of providing care, treatment and support to patients and carers. | Evaluation; UK; LOW |
| Association of Directors of Adult Social Services (ADASS) | 2010 | Carers as Partners in Hospital Discharge | *(No abstract on original paper – summary provided by current authors).* This is a short review undertaken by ADASS as part of their programme of work around government strategy for carers announced in 2008. The aim of the review was to explore joint working and the potential to improve outcomes for patients and carers around hospital discharge. The findings were informed by: a literature review; discussions with stakeholders, including health, social care and carers organisations, a carers’ reference group, and relevant social care leads; and linking in to other relevant projects, research programmes and reports. The report concluded that ‘that whilst there is good practice and progress in some places, there is still a continuing need to change attitudes and cultures and to better understand their impact on carers’ and provided a list of twelve key points for health and social care professionals and carers to work towards. These included ensuring that: statutory services have a lead on carers; specialist support is available for people with dementia; information for carers is available and accessible; operational cultures support carers as partners in caring; and every hospital discharge is as good as the best. | Other (Multi-method study); UK; HIGH |
| Awata S | 2010 | New national health program against dementia in Japan: the medical center for dementia | Herein, the Medical Center for Dementia, which was introduced in 2008 as a new national health program in Japan, is reviewed from the perspective of the recent history of the national provision against dementia and the findings of a series of studies on the current status of medical care for dementia. The Medical Center for Dementia was developed to provide special medical services for dementia and connect with other community resources in order to contribute to building a comprehensive support network for demented patients. Specifically, the Medical Center for Dementia provides the following: (i) special medical consultation; (ii) differential diagnosis and early intervention; (iii) medical treatment for the acute stage of behavioral and psychological symptoms of dementia and concurrent medical conditions; (iv) education for general practitioners and other community professionals; (v) network meetings for the establishment of medical-medical and medical-care connection; and (vi) provision of information regarding dementia to the public. Special Medical Consultation Rooms would play an important role in the efficient functioning of the Medical Center for Dementia. In cooperation with municipal governments, the Medical Center for Dementia is also expected to play an important role in policy making and to improve the local status of medical care for people with dementia. | Literature review; Japan; HIGH |
| Ballard C, Khan Z, Clack H and Corbett A | 2011 | Nonpharmacological Treatment of Alzheimer Disease | Objective: To review the key nonpharmacological treatment approaches to the cognitive and functional symptoms of Alzheimer disease (AD). Methods: We searched and critically analyzed the most recent relevant literature pertaining to the nonpharmacological treatment of AD. Results: There is evidence from a modest number of well-conducted randomized controlled trials (RCTs) that various nonpharmacological approaches, including cognitive training, cognitive rehabilitation, and cognitive stimulation therapy (CST), confer modest but significant benefits in the treatment of cognitive symptoms in people with AD, and that there may be additive benefits in combination with cholinesterase inhibitor therapy. Cognitive rehabilitation also appears to result in functional benefits in AD. The modest number of RCTs focusing on cognitive training in AD is consistent with the results of larger cognitive training trials in healthy older people. However, there is no convincing evidence of any benefits associated with brain training games. Conclusion: An emerging evidence base indicates that different approaches to cognitive training and cognitive stimulation in people with AD confer modest but significant benefits. The best evidence base is for CST, although this approach is labour-intensive, and requires further evaluation of cost-effectiveness. There is currently no evidence that brain training games provide any significant benefit to people with AD. | Literature review; International; MEDIUM |
| Belleville S | 2007 | Cognitive training for persons with mild cognitive impairment | Recent randomized control trials and meta-analyses of experimental studies indicate positive effects of non-pharmacological cognitive training on the cognitive function of healthy older adults. Furthermore, a large-scale randomized control trial with older adults, independent at entry, indicated that training delayed their cognitive and functional decline over a five-year follow-up. This supports cognitive training as a potentially efficient method to postpone cognitive decline in persons with mild cognitive impairment (MCI). Most of the research on the effect of cognitive training in MCI has reported increased performance following training on objective measures of memory whereas a minority reported no effect of training on objective cognitive measures. Interestingly, some of the studies that reported a positive effect of cognitive training in persons with MCI have observed large to moderate effect size. However, all of these studies have limited power and few have used long-term follow-ups or functional impact measures. Overall, this review highlights a need for a well-controlled randomized trial to assess the efficacy of cognitive training in MCI. It also raises a number of unresolved issues including proper outcome measures, issues of generalization and choice of intervention format. | Literature review; International; HIGH |
| Birch D and Draper J | 2008 | A critical literature review exploring the challenges of delivering effective palliative care to older people with dementia. | Aim. This paper considers the challenges of delivering effective palliative care to older people with dementia and the possible strategies to overcome barriers to end-of-life care in these patients. Background. In UK alone, approximately 100 000 people with dementia die each year and as the number of older people increases, dementia is set to become even more prevalent. Dementia is a progressive terminal illness for which there is currently no cure. Patients dying with dementia have significant health-care needs and in recent years it has been recognised that palliative care should be made available to everyone regardless of diagnosis, as this improves comfort and quality of life. Despite this, patients dying with dementia are often still not given access to palliative care services. Method. A review of English language literature published after 1996 to the present day relating to older people with dementia during the terminal phase of their illness. Results. Twenty-nine articles met inclusion criteria for the review. Most originated from North America and UK and were mostly quantitative in nature. Four key themes were identified: difficulties associated with diagnosing the terminal phase of the illness (prognostication); issues relating to communication; medical interventions; and the appropriateness of palliative care intervention. Conclusions. This review reinforces the importance of providing appropriate palliative care to individuals suffering from end-stage dementia and identifies some of the barriers to extending such specialist palliative care provision. Relevance to practice. There is an urgent need to improve palliative care provision for older people with end-stage dementia and, in addition, more research is required on the needs of patients entering the terminal phase of dementia to assist the allocation of appropriate resources and training to ensure quality and equality in the provision of end-of-life care. | Literature review; International; HIGH |
| Borbasi S, Emmanuel E, Farrelly B and Ashcroft J | 2010 | A Nurse Practitioner initiated model of service delivery in caring for people with dementia | The increasing number of people with dementia in aged care facilities is reported to have a high burden of care among staff The Nurse Practitioner role can be beneficial in the provision of dementia care particularly when difficult and aggressive behaviour is being displayed. The model of service described in this paper is designed in such a way to make the outreach team contribute to staff sustainability. Such a service is different to other outreach services and focuses on a number of key deliverables. In practice, the service ensures that recommendations made by the team at the initial assessment are implemented. In addition, they work with the staff in managing the resident's behaviour, train and model suggestions for practice in interventions. Evaluation of the service is a work in progress and will highlight important aspects about the workforce for the improvement of quality of life for residents with dementia. | Program evaluation; Australia; HIGH |
| Bossen AL, Specht JP and McKenzie SE | 2009 | Needs of people with early-stage Alzheimer's Disease: reviewing the evidence | The focus of this literature search was on the needs of older adults with Alzheimer's disease (AD). Very little research has been published directly related to the concept of needs, so concepts were identified by implicitly pulling them from the foci of articles. Identified articles were written outside the framework of the person with AD-from the perspective of the providers and caregivers. To get the person's perspective, the search was expanded to find "self-identified" needs of the person with AD and by widening the medical subject heading terms. The collection of work found indicated retained awareness of the person with AD well into the disease process and that this awareness is not recognized by professionals or caregivers. The result is that older adults with AD are not consulted in determining their needs or having a voice in their plan of care. Also identified were the needs for early diagnosis, to be heard, for information | Literature review; USA; MEDIUM |
| Bradway C and Hirschman KB | 2008 | Working with families of hospitalized older adults with dementia: caregivers are useful resources and should be part of the care team | Families provide a considerable amount of informal care and support for older adults living with dementia. And when an older adult with dementia is hospitalized, family caregivers should be seen as important sources of information and included as valuable members of the health care team. This article describes a best-practice approach to working with families and includes recommendations for using the Information for the Hospital Team About a Patient with Memory Problems form. For a free online video demonstrating the use of this form, go to http://links.lww.com/A301. | Other (Report of survey); USA; MEDIUM |
| Brodaty H and Cumming A | 2010 | Dementia Services in Australia | Aim: To describe dementia services in Australia. Method: Limited review of current government policies and relevant papers. Results: Australians with dementia, currently estimated at 220 000, are expected to number 1.13 million by 2050. In response, Commonwealth and State Australian governments have developed comprehensive plans and systems including expansion of community care services and packages, improvement in quality of residential care and initiatives to address behavioural and psychological symptoms of dementia. Alzheimer's Australia, which continues to be a powerful advocate for improvement in services, has pioneered a prevention programme to delay dementia onset. Conclusion: Further developments should aim to increase awareness, stigma, enhance carer support improve timely diagnosis and support for special population groups, notably those from Indigenous and non-English speaking communities and those with younger onset dementia and correct relative underfunding for dementia research. Dementia care in Australia is well developed but gaps remain. | Other (Policy analysis); Australia; HIGH |
| Buettner LL, Yu F and Burgener SC | 2010 | Evidence Supporting Technology-Based Interventions for People with Early-Stage Alzheimer's Disease. | Innovative uses of technology offer many exciting possibilities for better care and improved quality of life for people with Alzheimer's disease and their families. This article provides empirical evidence through a systematic review of 10 studies involving technological advances. Although the studies reported have a small number of participants, the early results are positive. We believe the use of technology has the potential to save health care costs, ease caregiver stress, and help people with dementia live better, safer, and more fulfilling lives. | Literature review; International; MEDIUM |
| Caddell LS and Clare L | 2011 | Interventions supporting self and identity in people with dementia: A systematic review | Objectives: Recently, researchers have started to focus on ways to support self and identity in people with dementia. The purpose of this review is to discuss the main features of existing interventions aiming to support self and identity in people with dementia, to draw conclusions regarding the effectiveness of these interventions and to highlight the implications for future research. Method: Systematic review of intervention studies aiming to support self and identity in people with dementia. Results: Ten studies met the inclusion criteria. All reported some benefits to participants. There were significant methodological limitations and study quality was generally low. The interventions varied in terms of participant characteristics, content and outcome measures, making it difficult to draw overall conclusions about effectiveness. Conclusions: Interventions aiming to support self and identity in people with dementia are currently in the early stages of development. Firm recommendations cannot be made at this time. Further well-designed studies are required to provide more robust evidence. | Literature review; International; HIGH |
| Cameron MJ, Horst M, Lawhorne LW and Lichtenberg PA | 2010 | Evaluation of academic detailing for primary care physician dementia education | objective of this evaluation study was to assess the effect of academic detailing (AcD) as a strategy to increase early detection of dementia in primary care practice and to improve support and management of Alzheimer's disease and other dementia disorders by increasing communication and referrals to local community agencies. As designed for dementia education, AcD consisted of 15-minute educational sessions delivered in primary care practice offices. Twenty-nine visits were conducted by trained teams comprised of a physician and representatives of the Alzheimer's Association (AA) and Area Agency on Aging (AAA). A key outcome of the visits was increased knowledge of the specific programs and services available. In all, 77.4% rated the visit very effective, and follow-up evaluation suggests visits led to an increase in referral to these agencies (55%) and potentially enhanced early detection of dementia by physicians as measured by 35% making changes in the way they identify at-risk patients. | Other (Program evaluation); USA; HIGH |
| Carswell W, McCullagh PJ, Augusto JC, Martin S, Mulvenna MD, Zheng H, Wang HY, Wallace JG, McSorley K, Taylor B and Jeffers WP | 2009 | A review of the role of assistive technology for people with dementia in the hours of darkness | Assistive Technology (AT) has been utilized to support people with dementia (PwD) and their carers in the home. Such support can extend the time that PwD can remain safely at home and reduce the burden on the tertiary healthcare sector. Technology can assist people in the hours of darkness as well as during the day. The objective of this literature review is to evaluate reported healthcare technologies appropriate to night time care. This paper summarises and categorises the current evidence base. In all, 131 abstracts were returned from a database search, yielding fifty four relevant papers which were considered in detail. While night-time specific studies identified very few papers (4 papers, 7%), most of the more general AT findings could be adopted to benefit night-time assistance. Studies have used technology for prompting and reminding as loss of time and forgetfulness are major problems; for monitoring daily activities in a sensor enriched environment and utilised location aware technologies to provide information to enhance safety. Technology also supports a range of therapies to alleviate symptoms. Therapies include the delivery of music and familial pictures for reminiscing, the use of light therapy to enhance wellbeing and the provision of mental tasks to stimulate the brain and maintain activity levels. | Literature review; International; MEDIUM |
| Chaplin A | 2011 | Holistic care and environmental design: the future for dementia care | Purpose - This paper aims to outline the development of a new approach, using environmental design and non-drug-based interventions to support individuals with dementia to live independently and safely in their own homes. Although in its infancy, this approach is beginning to show how it can help to improve the mood, socialisation, and short-term memory of people with dementia and reduce the need for residential care or hospital admission. Design/methodology/approach - As a case study, this paper is based upon the development teams' observations, complemented by those of other key stakeholders. It first reviews the policy context and evidence for the scale of the problem and some psychological approaches such as reminiscence work, which can alleviate the symptoms. It then outlines the potential in home improvement work in "dementia-proofing" and "retro-fitting", to enhance reminiscence-based "life experience" work. Finally, the approach is illustrated via an individual example. Findings - The results so far - though not formally evaluated - suggest that design-based approaches may add significantly to the effectiveness of psychological management of dementia via reminiscence work; early results suggest a reduction in the "chemical cosh" of medication. Practical implications - This paper describes early developments in a new approach with great potential. In the long-term, it is hoped that this dementia care model can be rolled out for replication in any home improvement agency or social care setting. Originality/value - The impact of dementia is of increasing concern both for individuals and for public budgets. The potential in dementia-friendly environmental design to complement other psychological approaches is an example of the search for more holistic approaches that respect and work with the strengths of the individual, in contrast to purely medical approaches relying on medication and/or institutional care. | Qualitative study; UK; LOW |
| Chaur-Jong H, Chien-Chang L, Chuen-Chau C, Chih-Hsiung W and  Ta-Liang C | 2012 | Postoperative adverse outcomes in surgical patients with dementia: A retrospective cohort study | BACKGROUND: Dementia patients often present with coexisting medical conditions and potentially face higher risk of complications during hospitalization. Because the general features of postoperative adverse outcomes among surgical patients with dementia are unknown, we conducted a nationwide, retrospective cohort study to characterize surgical complications among dementia patients compared with sex- and age-matched nondementia controls. METHODS: Reimbursement claims from the Taiwan National Health Insurance Research Database were studied. A total of 18,923 surgical patients were enrolled with preoperative diagnosis of dementia for 207,693 persons aged 60 years or older who received inpatient major surgeries between 2004 and 2007. Their preoperative co morbidities were adjusted and risks for major surgical complications were analyzed. RESULTS: Dementia patients who underwent surgery had a significantly higher overall postoperative complication rate, adjusted odds ratio (OR) 1.79 (95 % confidence interval [CI] 1.72-1.86), with higher medical resources use, and in-hospital expenditures. Compared with controls, dementia patients had a higher incidence of certain postoperative complications that are less likely to be identified in their initial stage, such as: acute renal failure, OR = 1.32 (1.19-1.47); pneumonia, OR = 2.18 (2.06-2.31); septicemia, OR = 1.8 (1.69-1.92); stroke, OR = 1.51 (1.43-1.6); and urinary tract infection, OR = 1.62 (1.5-1.74). CONCLUSIONS: These findings have specific implications for postoperative care of dementia patients regarding complications that are difficult to diagnose in their initial stages. Acute renal failure, pneumonia, septicemia, stroke, and urinary tract infection are the top priorities for prevention, early recognition, and intervention of postoperative complications among surgical patients with dementia. Further efforts are needed to determine specific protocols for health care teams serving this population. | Cohort study; Taiwan; MEDIUM |
| Chenoweth L, Jeon Y, Merlyn T and Brodaty H | 2010 | A systematic review of what factors attract and retain nurses in aged and dementia care | Aim. To present evidence-based factors for the recruitment and retention of licensed nurses caring for older people and persons with dementia. Background. The international nurse shortage crisis is intensified in the aged and dementia care sector. Strategies to address this crisis rely on qualitative, quasi-experimental, anecdotal and unsubstantiated literature. Design. Systematic literature review. Method. Search terms 'nurse''nurses''nursing''clinical supervision''staff''staffing''staff mix''staff levels''recruitment''retention''aged care''gerontology''gerontological''dementia care''residential''nursing home,' were used in all possible combinations and applied in a wide range of relevant academic databases, with secondary hand searches of selected bibliographies. Results. Two hundred and twenty-six papers were retrieved and scanned, with 105 chosen for closer examination that were relevant to recruitment and retention strategies for dementia and aged care nursing. Twenty-five of the papers chosen for review were rated at level 2++ to 3, according to the guidelines of the National Institute for Health and Clinical Excellence (The NICE Guidelines Manual, National Institute for Health and Clinical Excellence, London). The 25 critically reviewed papers are organised as promising strategies for (1) nurse recruitment and (2) nurse retention. Conclusions. The intrinsic rewards of the caring role attract nurses to dementia and aged care. Essential strategies linking recruitment with retention are: careful selection of student nurse clinical placements and their ongoing supervision and education, training for skills, leadership and teamwork for new and existing nurses, increased staffing levels, pay parity across different health settings and family friendly policies. Relevance to clinical practice. A family-friendly, learning environment that values and nurtures its nursing staff, in the same way as nurses are expected to value and care for their patients and residents, is critical in ensuring their retention in dementia and aged care. | Literature review; International; HIGH |
| Chien L, Chi H, Guo J, Liao Y, Chang L, Chen C and Chou K | 2011 | Caregiver support groups in patients with dementia: a meta-analysis | Objectives: Meta-analysis studies of specific types of support groups are limited. We conducted a review and assessment of the effectiveness of support groups for caregivers of demented patients, and examined the impact of support group characteristics. Methods: A search of multiple, electronic databases including the Cochrane Library, Medline, PUBMED, and others was conducted; studies published between 1998 and 2009 were collected. Thirty quantitative journal articles that were true and quasi-experimental controlled trials on support groups for nonprofessional caregivers, including mutual support, psychoeducational, and educational groups were analyzed. Outcome indicators were psychological well-being, depression, burden, and social outcomes. Results: Support groups showed a significant positive effect on caregivers' psychological well-being (Hedge's g -0.44, 95% CI = -0.73, -0.15), depression (Hedge's g = -0.40, 95% CI = -0.72, -0.08), burden (Hedge's g = -0.23, 95% CI = -0.33, -0.13), and social outcomes (Hedge's g = -0.40, 95% CI = 0.09, 0.71). The use of theoretical models, and length and intensity of group sessions had a significant impact on the effect sizes for psychological well-being and depression. Ratio of female participation (for psychological well-being and depression) and average age (social outcomes) were significant predictor variables. Conclusions: Support groups benefit caregivers and findings of this meta-analysis serve as immediate guidance for group facilitators. Future research should include additional outcome variables with our defined factors on effectiveness collected as demographic characteristic data for comparison. A more comprehensive understanding of the effectiveness of support groups is indicated to enhance outcomes for caregivers and patients. | Other (Meta-analysis); International; HIGH |
| Chrisp T, Thomas B, Goddard W and Owens A | 2011 | Dementia timeline: journeys, delays and decisions on the pathway to an early diagnosis | Here we present the timeline for those who reach a memory assessment service based on research conducted with 31 people living with Dementia and 49 carers in one area of the UK. The study develops the findings of earlier timeline work by illuminating other stages on the journey as events and decision points. Two key stages of delay are exposed. Firstly, the period from first thinking something may be amiss to the point of first talking to someone about it. Secondly, the period from first talking to someone, to first contact with a healthcare professional (HCP). A third period emerged where delays may occur once contact with the healthcare system is made. The mean journey time from thinking that something may be amiss to beginning the formal process of diagnosis was around three years. On average there is a gap of about a year between thinking something may be amiss and first talking to a friend or family member about the problem. Further, it typically takes just under two and a half years for a person to move from thinking something may be amiss to first contact with a healthcare professional. The mean time from first contact with a healthcare professional to arrival at a memory assessment service was around 35 weeks; however, for 90% of people it was eight weeks or less. Implications for policies that aim to bring forward diagnosis are that the largest potential for achieving earlier diagnosis will be from encouraging people to contact healthcare professionals earlier. | Qualitative study; UK; MEDIUM |
| Clarke C L, Wilcockson J, Gibb C E, Keady J, Wilkinson H and Luce A | 2011 | Reframing risk management in dementia care through collaborative learning | Risk management is a complex aspect of practice which can lead to an emphasis on maintaining physical safety, which impacts on the well-being of people with dementia. Education for practitioners in risk management is particularly challenging because of its conceptual nature and diverse perceptions of risk between and within professional groups. The practice development research reported here formed one part of a multisite study and contributed to developing a risk assessment and management framework for use by practitioners in partnership with people with dementia and their families. Practice development research uses learning theories in the process of the research, and in so doing its intent is to not only create new knowledge but to view the research process as also a process of learning for those involved. Twenty practitioners from varying professions participated in five Collaborative Learning Groups, each of at least 2 hours duration, which were held over a 7-month period. Data analysis highlighted contradictions in the care system and in the professional s intention to practice in a person-centred way. These were expressed through the themes of: Seeking Certainty; Making Judgments; Team Working; Managing Complexity; Gathering and Using Information. | Qualitative study; UK; HIGH |
| Cohen-Mansfield J and Parpura-Gill A | 2008 | Practice style in the nursing home: dimensions for assessment and quality improvement | Objective Based on research staff observations during several studies in nursing homes and the findings of other studies, we propose a nomenclature of components of care for the elderly in nursing homes. The paper seeks thereby to operationalize those aspects of the nursing home practice style that can be improved. Methods This operationalization examines two main components (staff and institutional components) of practice style of care in nursing homes. Four domains characterize staff conduct (knowledge, practice style proficiency, flexibility and individualization of care and communication) and three domains define institutional conduct (staff support, resources and flexibility/rigidity of policies). Results The paper addresses critical aspects of staff conduct, and by extension, key features that require training, monitoring, and systemic change. Examples for each domain of practice style are provided. Conclusions After systematically reviewing the observations and findings it was concluded that enhancing practice styles in the nursing home requires knowledge, communication, flexibility, understanding, and genuine concern on the part of nursing home staff and administrators at all levels. We acknowledge and understand, of course, that changing practice styles in nursing homes is a difficult and time-consuming process. | Literature review; International; HIGH |
| Cohen-Mansfield J, Jensen B, Resnick B and Norris M | 2012 | Assessment and treatment of behaviour problems in dementia in nursing home residents: a comparison of the approaches of physicians, psychologists, and nurse practitioners | Objective: To compare physicians (MDs), psychologists (PhDs), and nurse practitioners (NPs) regarding their approach to dementia-associated behaviour problems in nursing home residents. Methods: A web-based questionnaire solicited information about symptoms, assessment methods, suspected etiologies, and interventions with respect to the last resident treated for dementia-associated behaviour problems. Results: Responses were obtained from 108 MDs, 38 PhDs, and 100 NPs. All groups relied similarly on information from the nursing staff, speaking with nursing assistants, and care team meetings in assessment; NPs were more likely to consult with family members. A standard assessment instrument was used most frequently by PhDs (50%), but this generally assessed cognitive status rather than problem behaviours. PhDs most frequently noted depression in residents. Groups were similar in attributing the behavioural symptoms to dementia and to underlying medical conditions, but PhDs were more likely to indicate depression, loneliness/boredom, staff-resident communication, and insufficient activities as etiologies. Use of pharmacological and nonpharmacological interventions of MDs and NPs were similar. PhDs reported least satisfaction with treatment. Conclusion: Awareness of similarities and differences in the approaches of different health professionals can facilitate interdisciplinary interaction in providing care for dementia-associated behaviour problems in nursing home residents. | Other (Report of survey); USA; HIGH |
| Collet J, E de Vugt M, Verhey FRJ and Schols JMGA | 2010 | Efficacy of integrated interventions combining psychiatric care and nursing home care for nursing home residents: a review of the literature | Background: Nursing home residents needing both psychiatric care and nursing home care for either somatic illness or dementia combined with psychiatric disorders or severe behavioural problems are referred to as Double Care Demanding patients, or DCD patients. Integrated models of care seem to be necessary in order to improve the well-being of these residents. Objectives: Two research questions were addressed. First, which integrated interventions combining both psychiatric care and nursing home care in DCD nursing home residents are described in the research literature? And second, which outcomes of integrated interventions combining both psychiatric care and nursing home care in DCD nursing home residents are reported in the literature? Method: A critical review of studies was done that involved integrated interventions combining both psychiatric care and nursing home care on psychiatric disorders and severe behavioural problems in nursing home patients. A systematic literature search was performed in a number of international databases. Results: Eight intervention trials, including four RCTs (2b level of evidence), were identified as relevant studies for the purpose of this review. Seven studies, three of which were RCTs, showed beneficial effects of a comprehensive, integrated multidisciplinary approach combining medical, psychiatric and nursing interventions on severe behavioural problems in DCD nursing home patients. Conclusions: Important elements of a successful treatment strategy for DCD nursing home patients include a thorough assessment of psychiatric, medical and environmental causes as well as programmes for teaching behavioural management skills to nurses. DCD nursing home patients were found to benefit from short-term mental hospital admission. This review underlines the need for more rigorously designed Studies to assess the effects of a comprehensive, integrated multidisciplinary approach towards DCD nursing home residents. | Literature review; International; HIGH |
| Connolly A, Sampson E and Purandare N | 2012 | End-of-life care for people with dementia from ethnic minority groups: a systematic review | A systematic review of the literature was conducted to examine the relationship between ethnic minority status and provision of end-of-life care for people with dementia. It included all empirical research on people with dementia or severe cognitive impairment or their caregivers and with ethnic minority people as a subgroup in examining an outcome involving end-of-life care processes or attitudes toward end-of-life care. Two authors independently rated quality of included studies; 20 studies met eligibility criteria and were included in the review: 19 quantitative and one qualitative. All articles were based in the United States, with African American, Hispanic, and Asian groups being the ethnic minorities. Artificial nutrition and other life-sustaining treatments were more frequent and decisions to withhold treatment less common in African American and Asian groups. The qualitative evidence, albeit limited, found that attitudes toward end-of-life care were more similar than different between different ethnic groups. Differences in hospice usage patterns were less consistent and potentially influenced by factors such as study setting and dementia severity. Caregivers' experiences differed between ethnic groups, whereas levels of strain experienced were similar. Disparities in end-of-life care for people with dementia from ethnic minority groups appear to exist and may be due to the double disadvantage of dementia and ethnic minority status. Further research is needed in other western multicultural countries, with a focus on prospective qualitative studies to understand the underlying reasons for these differences, not just their occurrence. | Literature review; International; MEDIUM |
| Cooper C, Balamurali T, Selwood A and Livingston G | 2007 | A systematic review of intervention studies about anxiety in caregivers of people with dementia | Background There is considerable literature on managing depression, burden and psychological morbidity in caregivers of people with dementia (CG). Anxiety has been a relatively neglected outcome measure but may require specific interventions. Objective To synthesise evidence regarding interventions that reduce anxiety in CGs. Methods Twenty-four studies met our inclusion criteria. We rated the methodology of studies, and awarded grades of recommendation (GR) for each type of intervention according to Centre for Evidence Based Medicine guidelines, from A (highest level of evidence) to D. Results Anxiety level was the primary outcome measure in only one study and no studies were predicated on a power calculation for anxiety level. There was little evidence of efficacy for any intervention. The only RCT to report significantly reduced anxiety involved a CBT and relaxation-based intervention specifically devised to treat anxiety, and there was preliminary evidence (no randomised controlled trials) that caregiver groups involving yoga and relaxation without CBT were effective. There Was grade B evidence that behavioural management, exercise therapies and respite were ineffective. Limitations Many interventions were heterogeneous, so there is some overlap between groups. Lack of evidence of efficacy is not evidence of lack of efficacy. Conclusions CBT and other therapies developed primarily to target depression did not effectively treat anxiety. Good RCTs are needed to specifically target anxiety which might include relaxation techniques. Some of the interventions focussed on reducing contact with the care recipients but caregivers may want to cope with caring and preliminary evidence suggests strategies to help CGS manage caring demands may be more effective. | Literature review; International; MEDIUM |
| Cooper C, Mukadam N, Katona C, Lyketos C, Ames D, Rabins P, Engedal K, De Mendonca Lima C, Blazer D, Teri L, Brodaty H and Livingston G | 2012 | Systematic review of the effectiveness of non-pharmacological interventions to improve quality of life of people with dementia | Background: People with dementia report lower quality of life, but we know little about what interventions might improve it. Methods: We systematically reviewed 20 randomized controlled trials reporting the effectiveness of non-pharmacological interventions in improving quality of life or well-being of people with dementia meeting predetermined criteria. We rated study validity with a checklist. We contacted authors for additional data. We calculated standardized mean differences (SMD) and, for studies reporting similar interventions, pooled standardized effect sizes (SES). Results: Pooled analyses found that family carer coping strategy-based interventions (four studies, which did not individually achieve significance; n=420; SES 0.24 (range 0.03-0.45)) and combined patient activity and family carer coping interventions (two studies, not individually significant; n=191; SES 0.84 (range 0.54-1.14)) might improve quality of life. In one high-quality study, a care management system improved quality of life of people with dementia living at home. Group Cognitive Stimulation Therapy (GCST) improved quality of life of people with dementia in care homes. Conclusion: Preliminary evidence indicated that coping strategy-based family carer therapy with or without a patient activity intervention improved quality of life of people with dementia living at home. GCST was the only effective intervention in a higher quality trial for those in care homes, but we did not find such evidence in the community. Few studies explored whether effects continued after the intervention stopped. Future research should explore the longer-term impact of interventions on, and devise strategies to increase, life quality of people with dementia living in care homes or at home without a family carer. | Literature review; International; HIGH |
| Cooper C, Selwood A, Blanchard M, Walker Z, Blizard R and Livingston G | 2009 | The determinants of family carers' abusive behaviour to people with dementia: Results of the CARD study | Background: Although dementia and elder abuse prevention are political priorities, there are no evidence-based interventions to reduce abuse by family carers. We have limited understanding of why some family caters, but not others in similar circumstances, behave abusively. We aimed to test our hypothesis, that more anxious dementia carers report more abusive behaviours, and dysfunctional coping strategies and caret burden mediate this relationship. Method: We interviewed 220 family/friend dementia carers from Essex and London Community Mental Health Teams. We used the revised Modified Conflict Tactics Scale to measure abuse. Results: More anxious and depressed carets reported more abuse; this relationship was mediated by using dysfunctional coping strategies and higher burden. Abuse was predicted by: spending more hours caring, experiencing more abusive behaviour from care recipients and higher burden. Limitations: This was a cross-sectional study so we cannot confirm directions of causality. While many carets were willing to report abusive actions, some may not have been and our numbers may be an underestimate. Conclusion: Anxious and depressed carers are particularly likely to report abusive behaviour when asked. Testing interventions directed at reducing carer anxiety, depression or changing unhelpful coping strategies, and/or reducing care recipient aggression where possible, is a logical and urgent next step. | Other (Cross-sectional study); UK; MEDIUM |
| Corbett A, Stevens J, Aarsland D, Day S, Moniz-Cook E, Woods R, Brooker D and Ballard C | 2012 | Systematic review of services providing information and/or advice to people with dementia and/or their caregivers | Background: Information is a key part of service provision to people with dementia and their carers, but there is no systematic review of the evidence. This study aimed to determine whether information services confer significant benefit for quality of life, neuropsychiatric symptoms and carer burden. Method: A systematic review of intervention studies in people with dementia was carried out, focussing predominantly on the provision of information and/ or advice. Results: Thirteen randomised controlled trials were identified. Two of the three studies measuring quality of life indicated benefit. Significant benefits were also evident for neuropsychiatric symptoms (points difference, -1.48; confidence interval, -2.11 to 0.86), but not carer burden. Most interventions included other key elements such as skills training, telephone support and direct help to navigate the medical and care system. Conclusion: There is some support for the value of information services, but studies are needed to determine the specific elements that are effective. | Literature review; International; MEDIUM |
| Cotelli M, Manenti R and Zanetti O | 2012 | Reminiscence therapy in dementia: a review | Dementia is a progressive disorder that impacts several cognitive functions. However, some aspects of cognitive function are preserved until late in the disease and can therefore be the targets of specific interventions. The rehabilitation of cognitive function disorders represents an expanding area of neurological rehabilitation, and it has recently attracted growing political, social and ethical attention. Here, we review the efficacy of reminiscence therapy to improve cognitive functions and/or mood. Available studies suggest that reminiscence therapy can improve mood and some cognitive abilities. Further studies, based on larger patient samples including placebo and control conditions, should be conducted to identify the optimal conditions for such treatment protocols. | Literature review; International; MEDIUM |
| Cotelli M, Manenti R, Zanetti O and Miniussi C | 2012 | Non-pharmacological intervention for memory decline | Non-pharmacological intervention of memory difficulties in healthy older adults, as well as those with brain damage and neurodegenerative disorders, has gained much attention in recent years. The two main reasons that explain this growing interest in memory rehabilitation are the limited efficacy of current drug therapies and the plasticity of the human central nervous and the discovery that during aging, the connections in the brain are not fixed but retain the capacity to change with learning. Moreover, several studies have reported enhanced cognitive performance in patients with neurological disease, following non-invasive brain stimulation i.e., repetitive transcranial magnetic stimulation and transcranial direct current stimulation to specific cortical areas]. The present review provides an overview of memory rehabilitation in individuals with mild cognitive impairment and inpatients with Alzheimer's disease with particular regard to cognitive rehabilitation interventions focused on memory and non-invasive brain stimulation. Reviewed data suggest that in patients with memory deficits, memory intervention therapy could lead to performance improvements in memory, nevertheless further studies need to be conducted in order to establish the real value of this approach. | Literature review; International; MEDIUM |
| Cross S M B, Broomfield N M, Davies R and Evans J J | 2008 | Awareness, knowledge and application of memory rehabilitation among community psychiatric nurses working with dementia | This paper aims to establish the extent to which memory rehabilitation is carried out by Community Psychiatric Nurses (CPNs) working with dementia; to investigate awareness and knowledge of memory rehabilitation and memory processes amongst CPNs; and to explore the factors that influence deployment of memory rehabilitation advice. Participants, consisting of sixty five Community Psychiatric Nursing staff working in Community Mental Health Teams for the Elderly (CMHTEs) in Greater Glasgow NHS, completed specially developed questionnaires exploring knowledge of memory processes and knowledge of memory strategies. Data were collected in a structured group format. It was found that CPNs consider delivery of memory rehabilitation strategies as part of their role, but report limited knowledge of the wide range of potential memory strategies and aids. Specialist training should address this gap. | Other (Report of survey); UK; MEDIUM |
| Damianakis T, Crete-Nishihata M, Smith KL, Baecker RM and Marziali E | 2010 | The psychosocial impacts of multimedia biographies on persons with cognitive impairments | PURPOSE: The purpose of this feasibility pilot project was to observe Alzheimer's disease (AD) and mild cognitive impairment (MCI) patients' responses to personalized multimedia biographies (MBs). We developed a procedure for using digital video technology to construct DVD-based MBs of persons with AD or MCI, documented their responses to observing their MBs, and evaluated the psychosocial benefits. Methods: An interdisciplinary team consisting of multimedia biographers and social workers interviewed 12 family members of persons with AD and MCI and collected archival materials to best capture the families' and patients' life histories. We filmed patients' responses to watching the MBs and conducted follow-up interviews with the families and patients at 3 and 6 months following the initial viewing. Qualitative analytic strategies were used for extracting themes and key issues identified in both the filmed and the interview response data.; RESULTS: Analysis of the interview and video data showed how evoked long-term memories stimulated reminiscing, brought mostly joy but occasionally moments of sadness to the persons with cognitive impairments, aided family members in remembering and better understanding their loved ones, and stimulated social interactions with family members and with formal caregivers.; IMPLICATION: This study demonstrates the feasibility of using readily available digital video technology to produce MBs that hold special meaning for individuals experiencing AD or MCI and their families. | Qualitative study; Canada; MEDIUM |
| Detweiler M, Sharma T, Detweiler J, Murphy P, Lane S, Carman J, Chudhary A, Halling M and Kim K | 2012 | What is the evidence to support the use of therapeutic gardens for the elderly? | Horticulture therapy employs plants and gardening activities in therapeutic and rehabilitation activities and could be utilized to improve the quality of life of the worldwide aging population, possibly reducing costs for long-term, assisted living and dementia unit residents. Preliminary studies have reported the benefits of horticultural therapy and garden settings in reduction of pain, improvement in attention, lessening of stress, modulation of agitation, lowering of as needed medications, antipsychotics and reduction of falls. This is especially relevant for both the United States and the Republic of Korea since aging is occurring at an unprecedented rate, with Korea experiencing some of the world's greatest increases in elderly populations. In support of the role of nature as a therapeutic modality in geriatrics, most of the existing studies of garden settings have utilized views of nature or indoor plants with sparse studies employing therapeutic gardens and rehabilitation greenhouses. With few controlled clinical trials demonstrating the positive or negative effects of the use of garden settings for the rehabilitation of the aging populations, a more vigorous quantitative analysis of the benefits is long overdue. This literature review presents the data supporting future studies of the effects of natural settings for the long term care and rehabilitation of the elderly having the medical and mental health problems frequently occurring with aging. | Literature review; International; HIGH |
| Dias A, Dewey ME, D'Souza J, Dhume R, Motghare DD, Shaji K S, Menon R, Prince M and Patel V | 2008 | The effectiveness of a home care program for supporting caregivers of persons with dementia in developing countries: A randomised controlled trial from Goa, India | Objectives: To develop and evaluate the effectiveness of a home based intervention in reducing caregiver burden, promoting caregiver mental health and reducing behavioural problems in elderly persons with dementia. Methodology and Principal Findings: This was a randomised controlled trial in which the person with dementia-caregiver dyad was randomly allocated either to receive the intervention immediately or to a waiting list group which received the intervention after 6 months. It was carried out in communities based in two talukas (administrative blocks) in Goa, India. Mild to moderate cases with dementia (diagnosed using the DSM IV criteria and graded using the Clinical Dementia Rating scale) and their caregivers were included in the trial. Community based intervention provided by a team consisting of Home Care Advisors who were supervised by a counselor and a psychiatrist, focusing on supporting the caregiver through information on dementia, guidance on behaviour management, a single psychiatric assessment and psychotropic medication if needed. We measured caregiver mental health (General Health Questionnaire), caregiver burden (Zarit Burden Score), distress due to behavioural disturbances (NPI-D), behavioural problems in the subject (NPI-S) and activities of daily living in the elder with dementia (EASI). Outcome evaluations were masked to the allocation status. We analysed each outcome with a mixed effects model. 81 families enrolled in the trial; 41 were randomly allocated to the intervention. 59 completed the trial and 18 died during the trial. The intervention led to a significant reduction of GHQ (-1.12, 95% CI -2.07 to -0.17) and NPI-D scores (-1.96, 95% CI -3.51 to -0.41) and non-significant reductions in the ZBS, EASI and NPI-S scores. We also observed a non-significant reduction in the total number of deaths in people with dementia in the intervention arm (OR 0.34, 95% Cl 0.01 to 1.03). Conclusion: Home based support for caregivers of persons with dementia, which emphasizes the use of locally available, low-cost human resources, is feasible, acceptable and leads to significant improvements in caregiver mental health and burden of caring. | Controlled Clinical Trial (CCT); India; HIGH |
| Doncaster E, McGeorge M and Orrell M | 2011 | Developing and implementing quality standards for memory services: the Memory Services National Accreditation Programme (MSNAP) | Background: The lack of a consistent model means that the quality and characteristics of memory services can vary greatly. Quality standards have been successfully applied in a range of healthcare settings which allow services to implement improvements where necessary. A nationally agreed set of quality standards would help fulfil this need for UK memory services. Objectives: To develop a set of standards for memory services to form the basis of a quality improvement initiative (Memory Services National Accreditation Programme MSNAP]). Method: The standards development process involved five main elements: Literature review/content analysis; key stakeholder workshop; email and postal consultation; consensus meeting; and final consultation/obtaining endorsements. Thirteen memory services in the northwest of England participated in the pilot programme, during which the draft set of quality standards were applied through the processes of self review and peer review. Results: The finalised version consisted of 148 quality standards categorised along the following domains: management; resources available to support assessment and diagnosis; assessment and diagnosis; and ongoing care management and follow-up. The pilot stage highlighted standards representing common areas where improvements had been made, such as ascertaining whether the patient wished to know their diagnosis, and areas where more attention was still required, for example surveying referrers, patients and carers about their experiences of the service. Conclusion: It was possible to develop and field test nationally agreed quality standards for memory services. We believe that by implementing MSNAP it will be possible to improve the quality of UK memory services. | Other (Service description); UK; HIGH |
| Douglas-Dunbar M and Gardiner P | 2007 | Support for carers of people with dementia during hospital admission | The aim of this study was to help develop support services for carers of people with dementia on admission to a district general hospital. Qualitative methodology was used in the form of individual semi structured interviews. These interviews suggest that service developments need to take into account the individual need of each carer. Identified themes included communication, vulnerability of the carers and the need to develop a therapeutic relationship with the carer as well as the person with dementia. Recommendations for change include a letter introducing the dementia specialist nurse to be given to carers, a poster in wards across the trust to support the letter; and workshops on dementia care for staff with emphasis on the need to work in partnership with informal carers. | Qualitative study; UK; LOW |
| Durand M, James A, Ravishankar A, Bamrah JS and Purandare NB | 2009 | Domiciliary and day care services: Why do people with dementia refuse? | Objective: To explore the reasons given for refusal of day services, and to examine the relationship between willingness to accept day services and clinical variables. Method: Fifty people with dementia who lived alone and had refused day services were interviewed. Results: The most common reasons for reluctance to attend day services were the belief that they did not need day services, that they liked being on their own, and the belief that they would not enjoy it. People who persistently refused day services tended to have additional worries about meeting new people, losing their independence and being institutionalised. Fifty-four per cent of people with dementia who lived alone and had refused day services scored six or more on the Cornell Scale for Depression in Dementia, suggesting possible presence of major depression. Conclusion: In patients with dementia who live alone and refuse day services, their misconceptions about day services and possibility of undiagnosed depression need further exploration. | Other (Cross-sectional study); UK; MEDIUM |
| Dutton R | 2009 | Specialist community-based end of life dementia care nurse | In the UK, Housing 21 Dementia Voice has been awarded funding from the King s Fund for a two-year pilot project to employ a dementia specialist community-based End of Life Care Nurse to work within its dementia services team in London. The new “Dementia Voice Nurse” project is designed to fill a critical need identified by Tresham Dementia Services together with Westminster City Council and the local PCT: to be able to provide for people with dementia and their families appropriate assessments and end of life support services that others with different illnesses have access to. The project aims to address current key gaps by providing: specialist assessments for the person with dementia and their carer(s); advice and support for service users in their own homes; specialist training and support for Housing 21 care and support staff; and co-ordination of services. By considering the physical, psychological, social and spiritual needs of people with dementia and their carers, providing appropriate assessment, support and effective coordination of services, much-needed improvements in choice and care at the end of life can be achieved. | Qualitative study; UK; LOW |
| Egdell V, Bond J, Brittain K and Jarvis H | 2010 | Disparate routes through support: negotiating the sites, stages and support informal dementia care | Worldwide people with dementia are usually cared for at home by informal carers who may themselves have poor health and/or live in social situations which intensify their needs. The scale of these needs continues to be underappreciated and they are exacerbated by the limited social, cultural and emotional resources that carers can draw upon. This paper looks at the disparities in support, and the complex negotiations made by carers, as they reconcile the everyday realities of informal care in the home. Appreciation of these issues is essential in understanding carers' coping strategies in an ageing population. | Qualitative study; UK; HIGH |
| Elliott AF, Horgas AL and Marsiske M | 2008 | Nurses' role in identifying mild cognitive impairment in older adults | Mild cognitive impairment (MCI), a relatively new descriptive category, is believed to represent a stage between normal aging and early dementia. Nurse practitioners, who provide care for older adults across a variety of settings, are in a key position to detect early cognitive changes. The purpose of this study is to describe an approach to identifying MCI using a variety of measures and a consensus conference with neuropsychologists. The study was conducted in a sample of 130 elderly participants (aged 82.5 years; 81% female) residing in nursing homes, assisted living facilities, and senior housing. A team of clinicians (neuropsychologists and nurses) reviewed cognitive, mental health, and demographic data in consensus conference and classified study participants into 1 of 3 groups: cognitively intact (50.8%), amnestic MCI (19.2%), or probable dementia (30%). Discriminant function analysis (DFA) was used to independently classify individuals into cognitive status groups based on test scores alone and to compare quantitatively determined groups with consensus conference evaluations. The results indicate that the DFA correctly classified 95% of the participants. Further, results revealed a pattern in which persons with amnestic MCI have subtle memory impairments (similar to persons with dementia) but that more general cognitive functioning remains high (similar to intact persons). Nurse practitioners' heightened awareness of subtle distinctions in the dimensions of cognitive status associated with MCI can enhance their practice and assist them in making more informed referrals for dementia evaluations. | Other (Test of MCI screening tools); USA; HIGH |
| Evans G | 2009 | Improving end of life care for the person with dementia: A practical approach from general practice | Drawing on the experience of looking after 50 patients with dementia, this paper describes a practical approach by one GP to making a difference for people with dementia in care homes especially at the end of life. Included is the use of advance care plans and enhanced annual reviews drawing on GSF prognostic indicator guidance. Using case studies, the following topics are discussed: pain assessment, end of life care, emergency situations, hospital admission avoidance, swallowing difficulties, acute agitation and cardiopulmonary arrest. The forms and protocols which have been developed to support decision making in these situations, along with details of the website on which they can be found, are discussed. The potential benefits of significant event meetings and a GP practice to care home alignment model are also outlined. | Other (Case Studies); UK; HIGH |
| Faucounau V, Wu Y, Boulay M, De Rotrou J and Rigaud A | 2010 | Cognitive intervention programmes on patients affected by mild cognitive impairment: a promising intervention tool for MCI? | Purpose: This paper examines and reviews studies on the efficacy of computer-based cognitive intervention programmes in the elderly affected by Mild Cognitive Impairment (MCI). MCI patients are at higher risk to progress to dementia. Recent effort has been made to slow the cognitive decline and delay the onset of dementia in this population. Method: MEDLINE sources were searched with the following subject headings: computer-based cognitive intervention, cognitive stimulation, cognitive training, aging, elderly, cognitive impairment. Selected studies were quality assessed and data extracted by two reviewers. Results: Several studies reported encouraging results on cognitive interventions programmes as a means to improve cognitive abilities and emotional states and to decrease subjective memory complaints in MCI patients. Conclusion: Though both traditional and computer-based cognitive intervention programmes seem to be effective, the computer-based ones present more advantages: 1) they could individualize the programme tailored to the patient's neuropsychological pattern and needs. 2) they permit the user to make an immediate objective comparison with data collected earlier and thus help in setting up a systematic training plan by providing instant value-free feedback. 3) they offer a possibility of a wide scale dissemination. | Literature review; International; MEDIUM |
| Forbes D, Forbes S, Morgan D, Markle-Reid M, Wood J and Culum I | 2008 | Physical activity programs for persons with dementia | BACKGROUND: There is some evidence that physical activity delays the onset of dementia in healthy older adults and slows down cognitive decline to prevent the onset of cognitive disability. Studies using animal models suggest that physical activity has the potential to attenuate the pathophysiology of dementia. 'Physical activity' refers to 'usual care plus physical activity'.; OBJECTIVES: Primary: do physical activity programs maintain or improve cognition, function, behaviour, depression, and mortality compared to usual care in older persons with dementia? Secondary: do physical activity programs have an indirect positive impact on family caregivers' health, quality of life, and mortality compared to family caregivers of older persons with dementia who received usual care alone? Do physical activity programs reduce the use of health care services (e.g., visits to the emergency department) compared to usual care in older persons with dementia and their family caregiver?; SEARCH STRATEGY: The trials were identified from searches of the Specialized Register of the Cochrane Dementia and Cognitive Improvement Group, The Cochrane Library, MEDLINE, EMBASE, PsycINFO, CINAHL and LILACS on 9 September 2007 using the search terms: exercise OR "physical activity" OR cycling OR swim* OR gym* OR walk* OR danc* OR yoga OR "tai chi".; SELECTION CRITERIA: All relevant, randomized controlled trials in which physical activity programs were compared with usual care for the effect on managing or improving cognition, function, behaviour, depression, and mortality in people with dementia of any type and degree of severity. Secondary outcomes related to the family caregiver(s) included quality of life, mortality, and use of health care services were intended to be examined.; DATA COLLECTION AND ANALYSIS: Two reviewers independently assessed the retrieved articles for relevance and methodological quality, and extracted data from the selected trials. These were pooled were appropriate.; MAIN RESULTS: Four trials met the inclusion criteria. However, only two trials were included in the analyses because the required data from the other two trials were not made available. Only one meta-analysis was conducted. The results from this review suggest that there is insufficient evidence of the effectiveness of physical activity programs in managing or improving cognition, function, behaviour, depression, and mortality in people with dementia. Few trials have examined these important outcomes. In addition, family caregiver outcomes and use of health care services were not reported in any of the included trials.; AUTHORS' CONCLUSIONS: There is insufficient evidence to be able to say whether or not physical activity programs are beneficial for people with dementia. | Literature review; International; MEDIUM |
| Forsetlund L, Eike MC, Gjerberg E and Vist G | 2011 | Effect of interventions to reduce potentially inappropriate use of drugs in nursing homes: a systematic review of randomised controlled trials | BACKGROUND: Studies have shown that residents in nursing homes often are exposed to inappropriate medication. Particular concern has been raised about the consumption of psychoactive drugs, which are commonly prescribed for nursing home residents suffering from dementia. This review is an update of a Norwegian systematic review commissioned by the Norwegian Directorate of Health. The purpose of the review was to identify and summarise the effect of interventions aimed at reducing potentially inappropriate use or prescribing of drugs in nursing homes.; METHODS: We searched for systematic reviews and randomised controlled trials in the Cochrane Library, MEDLINE, EMBASE, ISI Web of Knowledge, DARE and HTA, with the last update in April 2010. Two of the authors independently screened titles and abstracts for inclusion or exclusion. Data on interventions, participants, comparison intervention, and outcomes were extracted from the included studies. Risk of bias and quality of evidence were assessed using the Cochrane Risk of Bias Table and GRADE, respectively. Outcomes assessed were use of or prescribing of drugs (primary) and the health-related outcomes falls, physical limitation, hospitalisation and mortality (secondary).; RESULTS: Due to heterogeneity in interventions and outcomes, we employed a narrative approach. Twenty randomised controlled trials were included from 1631 evaluated references. Ten studies tested different kinds of educational interventions while seven studies tested medication reviews by pharmacists. Only one study was found for each of the interventions geriatric care teams, early psychiatric intervening or activities for the residents combined with education of health care personnel. Several reviews were identified, but these either concerned elderly in general or did not satisfy all the requirements for systematic reviews.; CONCLUSIONS: Interventions using educational outreach, on-site education given alone or as part of an intervention package and pharmacist medication review may under certain circumstances reduce inappropriate drug use, but the evidence is of low quality. Due to poor quality of the evidence, no conclusions may be drawn about the effect of the other three interventions on drug use, or of either intervention on health-related outcomes. | Literature review; International; MEDIUM |
| Fortinsky R, Kulldorff M, Kleppinger A and Kenyon-Pesce L | 2009 | Dementia care consultation for family caregivers: collaborative model linking an Alzheimer's association chapter with primary care physicians | Objective: The primary objective is to report on the efficacy of an individualized dementia care consultation intervention for family caregivers of patients with diagnosed dementia living in the community. The secondary objective is to present evidence on the intervention process to inform the feasibility and sustainability of the model featuring collaboration between primary care physicians and a voluntary sector organization. Method: Randomization was based on primary care physician practice site. In the intervention group, dementia care consultants located at an Alzheimer's association chapter provided individualized counseling and support over a 12-month period, and sent copies of care plans developed with family caregivers to referring primary care physicians. In the control group, family caregivers received educational and community resource information but no care consultation. Nursing home admission of patients during the 12-month study period was the primary outcome; secondary outcomes included measures of caregiver self-efficacy for managing dementia, caregiver depressive symptoms, and caregiver burden. Results: A total of 84 family caregivers participated. After adjusting for baseline characteristics, patients whose family caregivers were in the intervention group were less likely than their control group counterparts to be admitted to a nursing home (Adjusted odds ratio = 0.40; 95% C.I. = 0.14-1.18; p = 0.10). No other outcomes were significantly different between treatment groups; however, intervention group caregivers reporting greater satisfaction with the intervention showed improved self-efficacy for managing dementia compared to their less satisfied counterparts. Medical record reviews found that care plans were found in most patient records, but that only 27% of intervention group caregivers reported discussing these care plans with physicians. Three different individuals occupied the dementia care consultant position during the study period, and this turnover led to family caregiver dissatisfaction. Conclusion: The dementia care consultation intervention showed favourable effects on nursing home admission and on caregiver outcomes among intervention group caregivers more satisfied with the intervention, but there are important barriers to sustaining this collaboration between primary care physicians and a voluntary sector organization such as an Alzheimer's association chapter. | Randomised Controlled Trial (RCT); USA: MEDIUM |
| Galik EM, Resnick B and Pretzer-Aboff I | 2009 | Knowing what makes them tick': Motivating cognitively impaired older adults to participate in restorative care | Nursing home residents with dementia represent a majority of the most functionally impaired individuals residing in nursing homes. Although many perceive this population as having little restorative potential, maintaining resident functional abilities for as long as possible helps to optimize quality of life and decrease caregiver burden. This study used a qualitative design with a focus group methodology to explore facilitators and barriers to engaging cognitively impaired residents in functional activities and exercise. A purposive sample of seven geriatric nursing assistants who were experts in dementia care participated in the study. Twenty-seven codes were reduced to three themes: (i) knowing what makes them tick and move; (ii) teamwork and utilizing resources; and (iii) barriers to restorative care. The study findings were used to revise the Restorative Care for the Cognitively Impaired Intervention and could direct future implementation of programmes in nursing home settings. | Qualitative study; USA; HIGH |
| Galvin J and Sadowsky C | 2012 | Practical guidelines for the recognition and diagnosis of dementia | To date, user-friendly, practical guidelines for dementia have not been available for busy family physicians. However, the growing number of patients with dementia means that primary care physicians will have an increasingly important role in the diagnosis and subsequent management of dementia. This article provides practical guidance for the recognition and diagnosis of dementia and is aimed at family physicians, who are usually the first clinicians to whom patients present with dementia symptoms. Because Alzheimer disease (AD) is the most common form of dementia, this condition is the main focus of this article. We review the pathophysiology of AD and discuss recommended diagnostic protocols and the importance of early diagnosis. An AD diagnostic algorithm is provided, with clearly defined steps for screening and diagnosing AD and assessing daily functioning, behavioural symptoms, and caregiver status. | Other (Practice guidelines); USA; HIGH |
| Ganzer C A | 2007 | Assessing Alzheimer's Disease and Dementia: Best Practices in Nursing Care | Alzheimer's disease (AD) is a chronic progressive neurodegenerative disorder that leads to irreversible dementia. As the number of older adults increases, so will the incidence of AD. The purpose of this article is to offer information to nurse clinicians regarding the differential diagnosis of dementia of the Alzheimer's type, describe available assessment instruments, and review practice recommendations for treatment. | Other (Description of screening tools); USA; LOW |
| Gavan J | 2011 | Exploring the usefulness of a recovery-based approach to dementia care nursing | Exploring new approaches to dementia care nursing is vital to enable services to cope with the expected rise in demand for healthcare due to an ageing population. A comparison between the current person-centred care approach in aged care and recovery-based approach that underpins mental health nursing was reviewed in the literature to determine which is more useful to dementia care nursing. The recovery model is the conceptual framework that underlies the recovery-based approach. It broadens the current person-centred care approach through the fostering of hope, facilitative rather than directive care, and enhances autonomy. This promotes positive outcomes for older people with dementia through empowerment to make choices in the way they wish to live within the community. This essay proposes that the recovery-based approach is more useful to dementia care nursing than person-centred models. | Literature review; International; MEDIUM |
| Geldmacher D S | 2007 | Treatment guidelines for Alzheimer's disease: redefining perceptions in primary care | The objective of this paper is to review current evidence and treatment patterns for pharmacotherapy in Alzheimer's disease (AD), with an emphasis on outcomes considered important to patients and families. The sources for the information are the peer-reviewed literature, Food and Drug Administration-approved package labelling for acetyl cholinesterase inhibitors (AChEIs), expert opinions expressed at the First Annual Dementia Congress, and clinical experience. Three AChEI agents are in routine use in the United States. They are considered part of the standard of care for patients with mild-to-moderate AD. There are differences in metabolism, pharmacokinetics, side effects, and ease of use that may influence the prescriber's choice of agent and dosage. The three approved agents have Similar outcomes in cognition and global clinician ratings of effectiveness in double-blind placebo controlled trials. Persistent therapy with effective doses of AChEIs is associated with reduced risk for, or delayed, nursing home placement, which is a stated priority of AD caregivers. Agents from this class of drugs have also been shown to be associated with statistically significant preservation of daily function and benefits in treatment of adverse behaviours in AD. Numerous additional choices are available to the clinician for pharmacotherapy of adverse behaviours. Community-based psycho educational support is also of value to caregivers. | Literature review; International; MEDIUM |
| Ghiotti C | 2009 | The Dementia End of Life Care Project (DeLCaP). Supporting families caring for people with late stage dementia at home | This article describes the development of a locally based project funded by the Big Lottery for 5 years from May 2008 to support five families at any one time caring for a relative with late stage dementia at home. The outcomes centre on improving the well-being of both the person with dementia and their caregivers. The assumption is that the principles of palliative care that have so sensitized the end of life care for people with cancer can be applied to improve the lot of people with dementia and their caregivers. Development of the project therefore entailed bringing together staff across dementia care, palliative care practitioners and crucial generalist community staff, such as GP's and District Nurses. A team with representation from these various professionals and care staff is now up and running to pilot a cross cutting service to meet the particular needs of people with late stage dementia and their families. The funding from the lottery includes additional respite care, training and consultancy. The project is small enough to enable experimentation and the design of a pathway to effectively utilize the knowledge and skills of the different practitioners involved. The scale of the project makes it possible to show how we can transcend the current underdevelopment and fragmentation of community services and help families who wish to do so, to care for their relatives at home. | Other (Study protocol); UK; MEDIUM |
| Gladman JRF, Jones RG, Radford K, Walker E and Rothera I | 2007 | Person-centred dementia services are feasible, but can they be sustained? | Background: we evaluated a specialist community-based dementia service to establish whether high quality care was being delivered and the conditions for doing so. The service was in an urban part of Rushcliffe Primary Care Trust, Nottinghamshire, United Kingdom. The service comprised an assessment team of an occupational therapist, a community psychiatric nurse and a community care officer, supported by 235 h per week of care delivered by a team of specially trained community care workers. Methods: a qualitative study was performed using non-participant observation, semi-structured interviews and focus groups, and analysed using a thematic framework approach. There were 2 focus groups involving staff, 11 interviews of staff and stakeholders, and interviews of 15 carers of people with dementia. Results: the care provided was appreciated by carers, and the service was approved by staff and stakeholders. Care was delivered using a rehabilitative style that aimed to maintain personhood, rather than to promote independence. Clients were usually referred with the object of preventing unwanted admission to institutional care but, over time, moving into an institution ceased to be a uniformly undesirable outcome. The service's resources were reduced during the evaluation period, in part to meet mental health needs in intermediate care services. Conclusions: an appropriately resourced and constructed specialist service using an adaptive rehabilitation approach aimed at maintaining personhood can deliver good individualised care to people with dementia, but specific and appropriate commissioning for these services is needed to nurture them. | Qualitative study; UK; HIGH |
| Gould E and Reed P | 2009 | Alzheimer's Association Quality Care Campaign and professional training initiatives: improving hands on care for people with dementia in the U.S.A. | In the U.S.A., direct care workers and licensed practical nurses are the professionals who provide the most hands-on care to people with dementia in nursing homes and residential care facilities - yet they do not receive adequate training in dementia care. Dementia care training needs to be universal with all disciplines at all levels of care. Even though there is variability on recommended hours and content, most studies emphasize the importance of dementia care training as a distinct component of required training for any professional or paraprofessional working in long-term care. In 2005, the Alzheimer's Association launched its Quality Care Campaign to improve dementia care through state and federal advocacy; consumer education and empowerment, and staff training. This paper describes the effectiveness of Alzheimer's Association training as measured by knowledge gained and providers' intention to change their behaviour immediately after attending the training. Overall, findings indicated that the participants responded positively to evidence-based training in dementia care that emphasized the importance of (i) leadership, (ii) team communication and collaboration, (iii) support and empowerment of direct care staff, (iv) awareness and practice of specific dementia care issues, (v) resident and family involvement in care, and (vi) professional self-care. | Qualitative study; USA; HIGH |
| Grand J, Casper S and MacDonald S | 2011 | Clinical features and multidisciplinary approaches to dementia care | Dementia is a clinical syndrome of widespread progressive deterioration of cognitive abilities and normal daily functioning. These cognitive and behavioral impairments pose considerable challenges to individuals with dementia, along with their family members and caregivers. Four primary dementia classifications have been defined according to clinical and research criteria: 1) Alzheimer's disease; 2) vascular dementias; 3) frontotemporal dementias; and 4) dementia with Lewy bodies/Parkinson's disease dementia. The cumulative efforts of multidisciplinary healthcare teams have advanced our understanding of dementia beyond basic descriptions, towards a more complete elucidation of risk factors, clinical symptoms, and neuropathological correlates. The characterization of disease subtypes has facilitated targeted management strategies, advanced treatments, and symptomatic care for individuals affected by dementia. This review briefly summarizes the current state of knowledge and directions of dementia research and clinical practice. We provide a description of the risk factors, clinical presentation, and differential diagnosis of dementia. A summary of multidisciplinary team approaches to dementia care is outlined, including management strategies for the treatment of cognitive impairments, functional deficits, and behavioral and psychological symptoms of dementia. The needs of individuals with dementia are extensive, often requiring care beyond traditional bounds of medical practice, including pharmacologic and non-pharmacologic management interventions. Finally, advanced research on the early prodromal phase of dementia is reviewed, with a focus on change-point models, trajectories of cognitive change, and threshold models of pathological burden. Future research goals are outlined, with a call to action for social policy initiatives that promote preventive lifestyle behaviors, and healthcare programs that will support the growing number of individuals affected by dementia. | Literature review; International; HIGH |
| Hägglund D | 2010 | A systematic literature review of incontinence care for persons with dementia: the research evidence | Background. Urinary/faecal incontinence in persons with dementia is a potentially treatable condition. However, which type of incontinence care is most appropriate for persons with dementia remains undecided. Aim. The aim of this study was to perform a systematic review of literature on incontinence care in persons with dementia focusing on assessment/management and prevention. Design. A systematic search of the literature. Method. The search was performed in the CINAHL, PubMed and Cochrane Library databases. Results. Of the 48 papers analysed, two were systematic literature reviews of management of urinary incontinence including persons with dementia. These reviews showed that the best-documented effect of toilet assistance for urinary incontinence in elderly persons with/without dementia had prompted voiding. However, prompted voiding in persons with dementia raises ethical concerns related to the person's integrity and autonomy. Timed voiding in combination with additional interventions like incontinence aids, staff training on the technique of transferring participants from bed to commode and pharmacological treatment decreased the number of urinary incontinence episodes in older persons with/without dementia. There is good scientific evidence that prevention of urinary incontinence in elders with/without dementia decreases incontinence or maintains continence. However, the evidence is insufficient to describe the state of knowledge of faecal incontinence. Conclusions. Toilet assistance, including timed voiding in combination with additional interventions and prompted voiding, are the available evidence-based interventions; however, nursing incontinence care is an experience-based endeavour for persons with dementia. Relevance to clinical practice. There is a lack of evidence-based nursing interventions related to incontinence care for persons with dementia. More research is needed to show whether experience-based incontinence care is effective and which activities are most appropriate for persons with dementia. However, the practice of effective nursing will only be realised by using several sources of evidence, namely research, clinical experience and patient experience. | Literature review; International; MEDIUM |
| Hailey D, Roine R and Ohinmaa A | 2008 | The effectiveness of telemental health applications: a review | OBJECTIVE: To review the evidence of benefit from use of telemental health (TMH) in studies that reported clinical or administrative outcomes.; METHOD: Relevant publications were identified through computerized literature searches using several electronic databases. Included for review were scientifically valid articles that described controlled studies, comparing TMH with a non-TMH alternative, and uncontrolled studies that had no fewer than 20 participants. Quality of the evidence was assessed with an approach that considers both study performance and study design. Judgments were made on whether further data were needed to establish each TMH application as suitable for routine clinical use.; RESULTS: Included in the review were 72 papers that described 65 clinical studies; 32 (49%) studies were of high or good quality. Quality of evidence was higher for Internet- and telephone-based interventions than for video conferencing approaches. There was evidence of success with TMH in the areas of child psychiatry, depression, dementia, schizophrenia, suicide prevention, posttraumatic stress, panic disorders, substance abuse, eating disorders, and smoking prevention. Evidence of success for general TMH programs and in the management of obsessive-compulsive disorder were less convincing. Further study was judged to be necessary or desirable in 53 (82%) of the studies.; CONCLUSION: Evidence of benefit from TMH applications is encouraging, though still limited. There is a need for more good-quality studies on the use of TMH in routine care. The emerging use of Internet-based applications is an important development that deserves further evaluation. | Literature review; International; MEDIUM |
| Hall W J | 2012 | Telemonitoring did not reduce hospitalizations or ED visits in high-risk elderly patients | QUESTION In elderly patients at high risk for hospitalization, does home telemonitoring reduce hospitalizations and emergency department (ED) visits compared with usual care? METHODS DESIGN Randomized controlled trial. ClinicalTrials.gov NCT01056640. ALLOCATION {Concealed}*. BLINDING Blinded ({outcome assessors}* and data analysts). FOLLOW-UP PERIOD 1 year. SETTING 4 primary care clinics in Minnesota, USA. PATIENTS 205 patients >60 years of age (mean age 80 y, 54% women) who were enrolled in the clinic's Employee and Community Health program and had an Elder Risk Assessment Index score >15 (based on age, sex, previous hospitalizations, and comorbid conditions stroke, dementia, heart disease, diabetes mellitus, and chronic obstructive pulmonary disease]). Exclusion criteria were residence in a nursing home, dementia, Kokmen mental status score 29, and inability to use the telemonitoring device. INTERVENTION Home telemonitoring using the Intel Health Guide (Intel-GE) device (n =102) or usual care (n =103). Telemonitoring comprised daily patient entry of symptoms and biometrics using peripheral scales, blood pressure cuff, glucometer, pulse oximeter, and peak flow meter. Data were reviewed daily by registered nurses who triaged patients using decision support from the medical record, consulted with primary physicians, and communicated with patients by telephone or videoconference as needed. OUTCOMES Primary outcome was a composite of hospitalizations and ED visits. Secondary outcomes included hospitalizations, ED visits, and mortality. With 100 patients per group, the study had 80% power to detect a 36% relative reduction (from 38% to 24%) in the composite endpoint at 1 year (alpha =0.05). PATIENT FOLLOW-UP 81% (intention-to-treat analysis). MAIN RESULTS Telemonitoring did not reduce hospitalizations or ED visits, combined or alone, but increased mortality (Table). CONCLUSIONS In high-risk elderly patients, telemonitoring did not reduce hospitalizations or emergency department visits compared with usual care but increased mortality. Telemonitoring vs usual care in high-risk elderly patients Outcomes Event rates At 1 yTelemonitoring Usual care RRI (95% CI)NNH (CI)Hospitalizations and ED visits64%57%11% (-11 to 40)NSHospitalizations52%44%19% (-11 to 59)NSED visits35%28%25% (-16 to 88)NSMortality15%3.9%279% (38 to 961)10 (6 to 33)ED = emergency department; NS = not significant; other abbreviations defined in Glossary. RRI, NNH, and CI calculated from event rates in article. | Randomised Controlled Trial (RCT); USA; LOW |
| Han JH, Bryce SN, Ely W, Kripalani S, Morandi A, Shintani A, Jackson J C, Storrow AB, Dittus RS and Schnelle J | 2011 | The effect of cognitive impairment on the accuracy of the presenting complaint and discharge instruction comprehension in older emergency department patients | Study objective: We seek to determine how delirium and dementia affect the accuracy of the presenting illness and discharge instruction comprehension in older emergency department (ED) patients. Methods: This cross-sectional study was conducted at an academic ED from May 2008 to July 2008 and included non-nursing home patients aged 65 years and older. Two open-ended interviews were performed to assess patients' ability to accurately provide their presenting illness and comprehension of their ED discharge instructions. The surrogates' version of the presenting illness and printed discharge instructions were the reference standards. Concordance between the patient and the reference standards was determined by 2 reviewers using a 5-point scale ranging from 1 (no concordance) to 5 (complete concordance). Proportional odds logistic regression was performed to determine whether cognitive impairment was associated with presenting complaint accuracy and discharge instruction comprehension. All models were adjusted for age, health literacy, education, non-white race, and hearing impairment. Results: For the presenting illness analysis, 202 patients participated. Compared with patients without cognitive impairment, those with delirium superimposed on dementia (DSD) had lower odds of agreeing with their surrogates with regard to why they were in the ED (adjusted proportional odds ratio=0.20; 95% confidence interval CI] 0.09 to 0.43). For the discharge instruction comprehension analysis, 115 patients participated. Patients with DSD had significantly lower odds of comprehending their discharge diagnosis (adjusted proportional odds ratio=0.13; 95% Cl 0.04 to 0.47), return to the ED instructions (adjusted proportional odds ratio=0.18; 95% Cl 0.04 to 0.82), and follow-up instructions (adjusted proportional odds ratio=0.09; 95% Cl 0.02 to 0.35) compared with patients without cognitive impairment. Conclusion: DSD is associated with decreased accuracy of the older patient's presenting illness and decreased comprehension of ED discharge instructions. | Other (Cross-sectional study); USA; MEDIUM |
| Harland JA Bath PA | 2008 | Understanding the information behaviours of carers of people with dementia: A critical review of models from information science | Objectives: The aim of this review is to discuss how existing models of information behaviour may help to improve provision of information to carers of people with dementia. The article analyses existing models of information behaviour derived from information science, describes studies that have examined these models in relation to health and discusses ways in which they help to understand the information behaviours of carers of people with dementia. Methods: A comprehensive review of the literature on studies of information needs and a critical examination of models of information behaviours in relation to health were undertaken. Results: Two dominant paradigms in information science research were identified, involving system-centred and user-centred approaches. System-centred approaches and studies are limited in that they have made assumptions on the types of information that people may require, the way in which information should be provided, the timing of information provision and have homogenous treated groups, failing to take account of individual preferences. In contrast, user-centred approaches recognise the unique needs of individuals and that information needs are subjective and affected by a variety of factors. User-centred models of information behaviour, particularly Dervin's sense-making theory and Wilson's Information Seeking Behaviour Models, could be useful in developing a better understanding of the information behaviours of carers of people with dementia. Conclusion: Adopting a user-centred approach to study the information behaviours of carers of people with dementia will take account of individual needs. Testing existing models of information behaviour within this group may help to develop interventions to meet the needs of individual carers and people with dementia. | Literature review; International; MEDIUM |
| Hertfordshire County Council Adult Care Services | 2009 | Flexicare housing Hertfordshire: Accommodation services for older people 2009 | Hertfordshire's Accommodation Services for Older People programme was launched in 2008 following a detailed analysis of the market for older people's accommodation. The programme is increasing the number of units and the range of accommodation available. It is an innovative partnership between, the county council, the district and borough councils, two Primary Care Trusts and local housing and care providers. The main focus is on providing more flexicare housing. Flexicare housing is Hertfordshire's unique version of extra care housing. It allows residents to rent or even own their own flat with on-site, flexible care available 24 hours a day. This strategy guide is published by Hertfordshire County Council and its partners. Its use of Flexicare represents a model of extra care housing that comprises a balanced community of older people ranging from those with little or no care needs to those with needs equivalent to residential care, including dementia. | Other (Service description); UK; MEDIUM |
| Iliffe S, Wilcock J, Griffin M, Jain P, Thune-Boyle I, Koch T and Lefford F | 2010 | Evidence-based interventions in dementia: A pragmatic cluster-randomised trial of an educational intervention to promote earlier recognition and response to dementia in primary care | Background: The National Dementia Strategy seeks to enhance general practitioners' diagnostic and management skills in dementia. Early diagnosis in dementia within primary care is important as this allows those with dementia and their family care networks to engage with support services and plan for the future. There is, however, evidence that dementia remains under-detected and sub-optimally managed in general practice. An earlier unblinded, cluster randomised controlled study tested the effectiveness of educational interventions in improving detection rates and management of dementia in primary care. In this original trial, a computer decision support system and practice-based educational workshops were effective in improving rates of detecting dementia although not in changing clinical management. The challenge therefore is to find methods of changing clinical management. Our aim in this new trial is to test a customised educational intervention developed for general practice, promoting both earlier diagnosis and concordance with management guidelines. Design/Method: The customised educational intervention combines practice-based workshops and electronic support material. Its effectiveness will be tested in an unblinded cluster randomised controlled trial with a pre-post intervention design, with two arms; normal care versus the educational intervention. Twenty primary care practices have been recruited with the aim of gaining 200 patient participants. We will examine whether the intervention is effective, pragmatic and feasible within the primary care setting. Our primary outcome measure is an increase in the proportion of patients with dementia who receive at least two dementia-specific management reviews per year. We will also examine important secondary outcomes such as practice concordance with management guidelines and benefits to patients and carers in terms of quality of life and carer strain. Discussion: The EVIDEM-ED trial builds on the earlier study but the intervention is different in that it is specifically customised to the educational needs of each practice. If this trial is successful it could have implications for the implementation of the National Dementia Strategy. | Randomised Controlled Trial (RCT); UK; LOW |
| JedeniusE, Johnell K, Fasbom J, Stromoqvist J, Winblad B and Andreasen N | 2011 | Dementia management programme in a community setting and the use of psychotropic drugs in the elderly population | Background and objective. The number of elderly persons in society is increasing, placing additional demands on the public health system. Extensive use of drugs is common in the elderly, and in patients with dementia this further increases their vulnerability. Since 1998 the municipality of Kalmar, Sweden, has worked with a dementia management programme that focuses on early intervention in order to identify the patient's help needs at an early stage. An important part of the programme aims at optimizing pharmacological treatment. The objective of the present study is to evaluate whether the dementia programme had a secondary effect on the use of psychotropic medication in the elderly population in general. Design and setting. A retrospective, drug utilization study analysing the use of selected drug categories by the elderly (75 years and older) in the Kalmar municipality compared with the whole of Sweden. Results and conclusions. The results suggest that the dementia programme contributed to an improvement in psychotropic drug use in the elderly as a secondary effect. Furthermore, the implementation of this programme did not require allocation of extra funding. | Other (Retrospective utilisation study); Sweden; MEDIUM |
| Joint Improvement Team | 2010 | An assessment of the development of telecare in Scotland 2006-2010 | Between August 2006 and March 2010, the Scottish Government made £16.35 million available under the Telecare Development Programme (TDP) to drive the adoption of telecare by local social and health care service providers. This report presents data on the growth of telecare through TDP funding and assesses progress against the original business case. Key findings include: over 29,000 people began a telecare service through TDP funding over the period 2006-2010; and more than 2,000 people that received a TDP funded service are known to have been diagnosed with dementia, although the true figure is thought to be a lot higher. By 31st March 2010, approximately £10.4 million of TDP funding £2.6 million of match funding had been spent. Around 1,500 hospital discharges were expedited as a result of TDP funding (the business plan anticipated 1,800) and 6,600 unplanned hospital admissions avoided (against 3,800 anticipated). The gross value of TDP funded efficiencies was approximately £48.4 million; fairly close to expectations. Assessed against the wider telecare strategy some key elements were achieved but important aspects of the vision for 2010 are still outstanding. There also remain questions about the extent to which mainstreaming of telecare has been achieved by some partnerships. | Other (Program evaluation); UK; MEDIUM |
| Jolley D and Moniz-Cook E | 2009 | Memory clinics in context | The growing number of older people in all parts of the world raises the question of how best to respond to their health needs, including those associated with memory impairment. Specialist Memory Clinics have a role to play, complementing community services which reach out to older people with mental health problems and encompassing younger people who become forgetful. Dementia is the most common syndrome seen, but there are other important treatable conditions which present with subjective or objective dysmnesia. Memory Clinics provide a high quality, devoted focus for early intervention, treatment, support and research. | Literature review; International; LOW |
| Jones K | 2010 | Integrated care pilot programme: Ensuring people with dementia receive joined up care | The Department of Health's integrated care pilots, announced in April 2009, aim to transform the way people experience health and social care. A multidisciplinary team in Bournemouth and Poole has set up a nurse led project focusing on memory loss and dementia in older people. This article outlines the aims, elements, challenges and benefits of working as part of a multidisciplinary team, from a nursing perspective. | Other (Program evaluation); UK; HIGH |
| Kada S, Nygaard HA, Geitung JT, Mukesh BN, Naik M, Wold G and Soevik DH | 2007 | International exchange. Quality and appropriateness of referrals for dementia patients | Objective To evaluate the quality and appropriateness of referrals from general practitioners (GPs) to geriatricians of patients with suspected dementia. Design A retrospective review of referrals from primary health care to a department of geriatric medicine. A data sheet was developed from a review of previous literature. Two GPs and two geriatricians assessed the quality and appropriateness of the referrals.; Setting Patient records in the geriatric department were collected, registered and scrutinised.; Subjects A total of 135 first-time referrals from January 2002 to December 2002 were evaluated. All patients and relatives were informed that participation was voluntary and anonymity was guaranteed.; Main outcomes Assessment of the appropriateness of referrals.; Results The mean age of all referred patients was 78.7 years (standard deviation (SD) 7.3; range 42-90 years) and 61.5% were female; 81 (60.0%) referrals were initiated by GPs, 33 (24.4%) by family members, three (2.2%) by community nurses, nine (6.7%) by the patients themselves and referral initiation was not specified for nine (6.7%). The agreement on appropriateness of referrals between the geriatricians was 83.7% (kappa 0.67; 95% confidence interval (CI) 0.55-0.79; P = 0.03) and the GPs was 71.1% (kappa 0.21; 95% CI 0.07-35.3; P< 0.001). After consensus, the agreement between the geriatricians and GPs was 57.8% (kappa 0.08; 95% CI 0-0.23). This difference was statistically significant (P< 0.001).; Conclusion There was disagreement between geriatricians and GPs regarding the appropriateness of referrals. It was found that time-consuming tests were infrequently performed or reported, and key medical information was absent from the referral letters. | Other (Retrospective case review); Norway; MEDIUM |
| Kaur H, Jutlla K, Moreland N and Read K | 2010 | How a link nurse ensured equal treatment for people of Asian origin with dementia | A review of services for older people with dementia from black and minority ethnic (BME) communities in Wolverhampton found that these groups were under represented in mental health services. Problems included a lack of information about and awareness of services, confusion about the mental health condition and a reluctance to ask for help. The review also highlighted that the proportion of older people with dementia from BME groups is set to increase significantly. It made several wide ranging recommendations to improve access to and uptake of services for these groups, which led to the creation of an Asian link nurse role specifically for Punjabi speaking people of Asian origin. This practitioner is a qualified community psychiatric nurse, who speaks Punjabi, related languages and English, and understands the relevant cultural issues. This model has been used elsewhere and we believe it should be adopted more widely to ensure equal access to culturally appropriate services for older people from BME groups. | Other (Service description); UK; LOW |
| Keiser L and Zasler N | 2009 | Residential design for real life rehabilitation | This article provides readers with a review of the major considerations for designing living environments for persons with neurodisability due to acquired brain injury (ABI). Components that need to be considered in order to assure that the environment is designed with a functional perspective in mind are explored. The issues to be considered herein include the influences of cognition and visual and visuoperceptual, motor, behavioural, and sensory impairment on residential design considerations. Resources for individuals involved in residential design for this special population are also provided to facilitate design decisions and implementation. | Literature review; International; HIGH |
| Kelley AS, Siegler EL and Reid C | 2008 | Pitfalls and recommendations regarding the management of acute pain among hospitalized patients with dementia | Objective. Older adults with dementia are frequently hospitalized, and a substantial minority present with (or develop) pain during hospitalization. Although general pain management guidelines are available, care can prove challenging in the setting of dementia. The purposes of this study were to review cases of older demented adults with pain admitted to an inpatient geriatric medicine service, and to identify difficulties in their management, which arise as a consequence of patients' dementia. Design. Case series. Setting. An urban tertiary care hospital located in New York City. Patients. Adults aged 70 years and older with dementia and pain. Results. Patients with dementia and pain may be 1) unable to describe the qualitative characteristics and associated features of their pain; 2) less able than cognitively intact older adults to alert their care providers to the presence of side effects from pain medicines; and 3) unable to discern variations in the level of pain or compare their current pain to their experience of the day or hours before. These deficits can lead to a delayed or incorrect diagnosis, suffering due to side effects, and overtreatment, which can lead to complications like delirium, bowel problems, and prolong length of stay. Conclusions. The cases presented herein highlight the need to conduct a thorough cognitive assessment of all older hospitalized patients with pain prior to implementing pain medicines. Research is needed to develop effective strategies for managing pain among demented elders in the acute-care setting. | Other (Case study); USA; MEDIUM |
| Khan F and Curtice M | 2011 | Non-pharmacological management of behavioural symptoms of dementia | This article describes a 6-month pilot project in which a community mental health team provided a dementia inreach service into 4 care homes in Birmingham, UK. The project included analysis of the impact of the service at the end of the project as well as a literature review of dementia care in care homes, and especially the issue of antipsychotic medication use and non-pharmacological approaches in managing behavioural and psychological symptoms of dementia (BPSD). The project included training care home staff in the management of BPSD; 2 questionnaires distributed at the beginning of the project found that 65% of care home staff felt a need for education and awareness, practical problem-solving and counselling in managing BPSD. Self-reported knowledge of common mental health problems and dementia increased in care home staff at the end of the project by a margin of 7% and 11% respectively. Reported confidence in managing behavioural problems increased by 9% among care home staff at the end of the project. The project achieved regular monitoring of psychotropic medications, and enabled the discharge of 14 out of 63 existing patients in the selected homes. The project also provided guidance for non-pharmacological techniques for management of BPSD, which included relaxation techniques, distraction techniques, reality orientation, reminiscence work, needs led therapy, music therapy, person-centred approach and behaviour therapy. | Qualitative study; USA; MEDIUM |
| Koch T and Iliffe S | 2010 | Rapid appraisal of barriers to the diagnosis and management of patients with dementia in primary care: a systematic review | Background: The diagnosis of dementia in primary care is perceived as a problem across countries and systems, resulting in delayed recognition and adverse outcomes for patients and their carers. Improving its early detection is an area identified for development in the English National Dementia Strategy 2009; there are thought to be multiple benefits to the patient, family, and resources by doing this. The aim of this review was to carry out a rapid appraisal in order to inform the implementation of this policy. Method: Publications in English up to August 2009 relating to barriers to the recognition of dementia, were identified by a broad search strategy, using electronic databases MEDLINE, EMBASE, and psycINFO. Exclusion criteria included non-English language, studies about pharmacological interventions or screening instruments, and settings without primary care. Results: Eleven empirical studies were found: 3 quantitative, 6 qualitative, and 2 with mixed methodologies. The main themes from the qualitative studies were found to be lack of support, time constraints, financial constraints, stigma, diagnostic uncertainty, and disclosing the diagnosis. Quantitative studies yielded diverse results about knowledge, service support, time constraints, and confidence. The factors identified in qualitative and quantitative studies were grouped into 3 categories: patient factors, GP factors and system characteristics. Conclusion: Much can still be done in the way of service development and provision, GP training and education, and the eradication of stigma attached to dementia, to improve the early detection and management of dementia. Implementation of dementia strategies should include attention to all three categories of barriers. Further research should focus on their interaction, using different methods from studies to date. | Literature review; International; HIGH |
| Koch T and Iliffe S | 2011 | Dementia diagnosis and management: a narrative review of changing practice | BACKGROUND: Early detection and management of dementia in primary care are difficult problems for practitioners. England's National Dementia Strategy 2009 seeks to improve these areas but there is limited evidence on how to achieve this most effectively.; AIM: This review aims to identify and appraise empirical studies of interventions designed to improve the performance of primary care practitioners in these areas.; DESIGN: A narrative review of primary-care based studies.; METHOD: Publications up to February 2010 were identified by searching the electronic databases MEDLINE, Embase, and PsycINFO, and bibliographies. The criterion for inclusion was that studies had to be of interventions aimed at improving detection or management of dementia in primary care. Exclusion criteria included studies in non-English publications, pharmacological interventions, and screening instrument studies. Quality was assessed using the PEDro (Physiotherapy Evidence Database) scale.; RESULTS: Fifteen studies were identified, of which 11 were randomised controlled trials. Eight reported educational interventions, and seven trialled service redesign, either by changing the service pathway or by introducing case management. Educationally, only facilitated sessions and decision-support software improved GPs' diagnosis of dementia, as did trials of service-pathway modification. Some of the case-management trials showed improved stakeholder satisfaction, decreased symptoms, and care that was more concordant with guidelines.; CONCLUSION: The quality of the studies varied considerably. Educational interventions are effective when learners are able to set their own educational agenda. Although modifying the service pathway and using case management can assist in several aspects of dementia care, these would require the provision of extra resources, and their value is yet to be tested in different health systems. | Literature review; International; HIGH |
| Kurz AF, Leucht S and Lautenschlager NT | 2011 | The clinical significance of cognition focused interventions for cognitively impaired older adults: a systematic review of randomized controlled trials | Background: Cognitive stimulation, training or rehabilitation can achieve modest, skill-specific gains in cognitively healthy older adults. With regard to the limited efficacy of currently available anti-dementia drugs it is crucial to investigate whether such treatments also provide clinically meaningful benefits to cognitively impaired older individuals. Methods: We conducted a systematic review of randomized controlled trials evaluating cognition-focused interventions in participants with mild cognitive impairment or dementia. Meta-analytic strategies were used to calculate effect sizes. Results: Cognition-focused interventions confer small and inconsistent effects on trained cognitive skills which, according to some studies, translate into gains on general cognitive ability. Instruments measuring such effects such as the Mini-Mental State Examination (MMSE) or the Alzheimer's Disease Assessment Scale, cognitive part (ADAS-Cog) show standardized mean differences of 0.20 and 0.30, respectively, which are comparable with those of current ant dementia drug treatments. However, convincing evidence of clinical significance was only obtained from single trials in terms of delay of cognitive decline, improvement in activities of daily living, or enhanced attainment of personally relevant goals. Conclusions: The potential of cognition-focused interventions has probably been obscured by the methodological inconsistencies and limitations of the clinical studies conducted thus far. Further randomized controlled trials on the efficacy of these treatment modalities are required using optimized and consistent methods. Emphasis should be placed on tailoring interventions to individual needs and resources while maintaining a high level of standardization, on implementing newly acquired skills and strategies in the everyday context, on appropriate treatment duration, and on including person-centred outcomes. | Literature review; International; MEDIUM |
| Lai J M and Karlawish J | 2008 | Assessing the capacity to make everyday decisions: A guide for clinicians and an agenda for future research | Assessing the capacity of patients to make decisions about their functional problems has substantial ethical, clinical, and financial implications. The growing population of older adults with cognitive impairment either in the community or in long-term care and medical facilities increase the importance of adequately assessing this capacity. This review examines the current approaches to making this assessment, demonstrates how they are incomplete, and considers potential approaches for improving these evaluations. Future research should develop and validate methods to identify patients with impaired capacity to make everyday decisions. These data will supplement functional, cognitive, and medical assessments. | Literature review; International; HIGH |
| Lauriks S, Reinersmann A, Van der Roest HG, Meiland FJM, Davies RJ, Moelaert F, Mulvenna MD, Nugent CD and Dröes RM | 2007 | Review of ICT-based services for identified unmet needs in people with dementia | Some of the needs that people with dementia and their informal carers currently perceive as insufficiently met by regular care and support services might be alleviated or even be met, using modern Information and Communication Technology (ICT). The study described in this paper was designed to provide an insight into the state of the art in ICT solutions that could contribute to meet the most frequently mentioned unmet needs by people with dementia and their informal carers. These needs can be summarized as (1) the need for general and personalized information; (2) the need for support with regard to symptoms of dementia; (3) the need for social contact and company; and (4) the need for health monitoring and perceived safety. Databases that were searched include: PubMed, Cinahl, Psychinfo, Google (Scholar), INSPEC and IEEE. In total 22 websites and 46 publications were included that satisfied the following criteria: the article reports on people with dementia and/or their informal carers and discusses an ICT-device that has been tested within the target group and has proven to be helpful. Within the first need area 18 relevant websites and three studies were included; within the second need area 4 websites and 20 publications were included. Within the third and fourth need area 11 and 12 publications were included respectively. Most articles reported on uncontrolled studies. It is concluded that the informational websites offer helpful information for carers but seem less attuned to the person with dementia and do not offer personalized information. ICT solutions aimed at compensating for disabilities, such as memory problems and daily activities demonstrate that people with mild to moderate dementia are capable of handling simple electronic equipment and can benefit from it in terms of more confidence and enhanced positive effect. Instrumental ICT-support for coping with behavioural and psychological changes in dementia is relatively disregarded as yet, while support for social contact can be effectively realized through, for example, simplified (mobile) phones or videophones or (entertainment) robots. GPS technology and monitoring systems are proven to result in enhanced feelings of safety and less fear and anxiety. Though these results are promising, more controlled studies in which the developed ICT solutions are tested in real life situations are needed before implementing them in the care for people with dementia. It is recommended that future studies also focus on the integration of the current techniques and solutions. | Literature review; International; HIGH |
| Lawrence V and Banerjee S | 2010 | Improving care in care homes: A qualitative evaluation of the Croydon care home support team | Objectives: The Croydon care home support team (CHST) was developed in response to reports of patient abuse within long-term care. It presents a novel strategy for improving standards of care within care homes. A qualitative methodology was used to assess the perceived impact of the CHST. Method: In-depth interviews were conducted with 14 care home managers and 24 members of care home staff across 14 care homes. Grounded theory principles guided the collection and analysis of the data. Results: Reports of improved communication between staff, improved staff development and confidence, and improved quality of care point towards the effectiveness of the CHST model. The collaborative approach of the CHST was considered pivotal to its success and presented as an effective method of engaging care home managers and staff. The CHST adopted a systemic approach that placed an equal emphasis on the social, mental health and nursing needs of residents and aimed to address the whole culture of care within the individual homes. Conclusions: The data demonstrate the potential for specialist multi-disciplinary teams to raise standards of care across long-term care settings. Increased awareness of safeguarding issues, improved staff morale and communication and ongoing opportunities for discussion and problem solving promised to sustain improvements. Such services could be instrumental in meeting the government priority of preventing abuse among vulnerable adults. | Qualitative study; UK; HIGH |
| Lawrence V and Murray J | 2010 | Balancing independence and safety: the challenge of supporting older people with dementia and sight loss | Objective: the study investigated attitudes towards working with older adults with concurrent sight loss and dementia, the challenges involved and suggestions for service development. Methods: a qualitative study was conducted comprising 18 in-depth interviews and two focus groups with care professionals within mental health and low vision services. Results: care professionals were alert to the high levels of risk among patients with joint sight loss and dementia. In-depth interviews revealed that insufficient time and expertise can lead to an overcautious approach that prioritises the reduction of risk rather than the promotion of independence. Focus groups highlighted the role that joint working can play in supporting older adults' valued roles and activities. Barriers to joint working were identified alongside strategies to assist the process. Conclusion: it is essential that care professionals and service providers acknowledge and respond to the complex needs of this population. Joint working was considered key to assessing risks and targeting interventions. The research workshop presented here provides a useful format for improving practice across inter-professional boundaries. | Qualitative study; UK; MEDIUM |
| Li H, Li J, Li N, Li B, Wang P and Zhou T | 2011 | Cognitive intervention for persons with mild cognitive impairment: a meta-analysis | Cognitive training for persons with mild cognitive impairment (MCI) has become a hot topic. However to date it remains controversial whether persons with MCI can really benefit from cognitive intervention. We aim to further investigate this by using meta-analysis of seventeen clinical studies of cognitive intervention for MCI. The results demonstrate that after training, patients with MCI improve significantly both in overall cognition and overall self-ratings. Specifically, persons with MCI obtain moderate benefits in language, self-rated anxiety and functional ability, and receive mild benefits in episodic memory, semantic memory, executive functioning/working memory, visuo-spatial ability, attention/processing speed, MMSE, self-rated memory problem, quality of life, activities of daily life and self-rated depression. The results also suggest that persons with MCI benefit from the cognitive intervention in the follow-up data. The present meta-analysis demonstrates that cognitive intervention can be a potential efficient method to enhance cognitive and functional abilities in persons with MCI, although the improvements may be domain-specific. | Literature review; International; HIGH |
| Li R, Cooper C, Bradley J, Shulman A and Livingston G | 2012 | Coping strategies and psychological morbidity in family carers of people with dementia: a systematic review and meta-analysis | Background: Carers for people with dementia experience high levels of anxiety and depression. Coping style has been associated with carer anxiety and depression. Method: We systematically reviewed studies examining the relationships between coping and anxiety or depression among carers of people with dementia. We rated study validity using standardised checklists. We calculated weighted mean correlations (WMC) for the relationships between coping and psychological morbidity, using random effects meta-analyses. Results: We included 35 studies. Dysfunctional coping correlated with higher levels of anxiety (WMC = 0.39, 95% CI 0.28-0.50; N = 688) and depression (0.46, 0.36-0.56; N = 1428) cross-sectionally, and with depression 6 and 12 months later (0.32, 0.10-0.54; N = 143). Emotional support and acceptance-based coping correlated with less anxiety (-0.22, 95% CI -0.26 to -0.18; N = 628) and depression (-0.20. -0.28 to -0.11; N = 848) cross-sectionally; and predicted anxiety and depression a year later in the only study to measure this. Solution-focused coping did not correlate significantly with psychological morbidity. Limitations: Just over a quarter of the identified studies provided extractable data for meta-analysis, including only two longitudinal studies. Conclusions: There is good evidence that using more dysfunctional, and less emotional support and acceptance-based coping styles are associated with more anxiety and depression cross-sectionally, and there is preliminary evidence from longitudinal studies that they predict this morbidity. Our findings would support the development of psychological interventions for carers that aim to modify coping style. | Literature review; International; HIGH |
| Lindsay S, Coyle M, Dunster B and Traynor V | 2010 | Collaborative model for end-stage dementia care | Healthcare practitioners working with people with dementia are often asked by the carer: 'When will my partner need to go to a nursing home?' This article presents an oveniew of how a local dementia care seNice identified and addressed a need to support carers and people with dementia in their own home towards the end of life. A case study is presented using a biographical narrative from a couple who were farmers in a rural community in New South Wales, Australia. It demonstrates how the choice of dying at home became a reality despite the challenges of living with dementia and being in a geographically isolated farming community. The authors describe a community collaborative model of care for end-stage dementia, using the client and carer experience to illustrate its practical application. To conclude, they explore how case studies can inform future practice initiatives. | Other (Case study); Australia; LOW |
| Low L, Yap M and Brodaty H | 2011 | A systematic review of different models of home and community care services for older persons | Background: Costs and consumer preference have led to a shift from the long-term institutional care of aged older people to home and community based care. The aim of this review is to evaluate the outcomes of case managed, integrated or consumer directed home and community care services for older persons, including those with dementia. Methods: A systematic review was conducted of non-medical home and community care services for frail older persons. MEDLINE, PsycINFO, CINAHL, AgeLine, Scopus and PubMed were searched from 1994 to May 2009. Two researchers independently reviewed search results. Results: Thirty five papers were included in this review. Evidence from randomized controlled trials showed that case management improves function and appropriate use of medications, increases use of community services and reduces nursing home admission. Evidence, mostly from non-randomized trials, showed that integrated care increases service use; randomized trials reported that integrated care does not improve clinical outcomes. The lowest quality evidence was for consumer directed care which appears to increase satisfaction with care and community service use but has little effect on clinical outcomes. Studies were heterogeneous in methodology and results were not consistent. Conclusions: The outcomes of each model of care differ and correspond to the model's focus. Combining key elements of all three models may maximize outcomes. | Literature review; International; MEDIUM |
| Maas M L, Specht J P, Buckwalter KC, Gittler J and Bechen K | 2008 | Nursing Home Staffing and Training Recommendations for Promoting Older Adults' Quality of Care and Life Part 1. Deficits in the Quality of Care Due to Understaffing and undertraining | Caught between the inability or unwillingness of nursing home corporations and owners to redistribute revenue and the reluctance of federal and state agencies to increase payments to nursing homes, the nation's most vulnerable older adults are not receiving the care they deserve. Widespread recognition of substandard care and quality of life of older adults in nursing homes has existed for decades. In addition, there is substantial evidence that poor quality of care is related to inadequate numbers and training of nursing staff. Still, policy makers and nursing home owners have failed to take needed action. In the first article of this two-part series, major deficits in the care of older adult nursing home residents are reviewed, and research documenting the relationship between nursing home staffing and the quality of care and life of residents is summarized. | Literature review; USA; HIGH |
| Malyuk R E, Wong C, Buree B, Kang A and Nirmal Kang N | 2011 | The interplay of infections, function and length of stay (LOS) in newly admitted geriatric psychiatry patients | The purpose of this study was to determine the impact of identifying and treating infections on functional outcomes and length of stay (LOS). Our retrospective naturalistic study reviewed all new admissions to a tertiary geriatric psychiatry teaching hospital from 2003 to 2007. Over this four-year period, 390 patients were admitted and discharged with 21% (85) of patients identified as having infections on admission. Those with infections were compared to the group without to determine and compare clinical characteristics. Factors included in analysis were: age, gender, diagnoses, medical co morbidity, neuropsychiatric symptoms, functional outcomes, medications and LOS. Both groups were similar in gender, psychiatric diagnoses and severity of dementia. Those requiring antibiotics for treatment of infections on admission, were older (p = 0.003), had poorer baseline function (p = 0.005) and higher medical co morbidity (p < 0.001). At discharge, the group with infections showed greater functional improvement (p < 0.001), particularly in mobility (p = 0.005) and cognition (p = 0.046), and had a shorter LOS (p = 0.02). We conclude that a significant number of patients in tertiary geriatric services continue to have infections on admission. Early identification and treatment of infections can result in improved function and decreased LOS. | Cohort study; Canada; HIGH |
| Martin S, Kelly G, Kernohan W, McCreight B and Nugent C | 2009 | Smart home technologies for health and social care support | Background The integration of smart home technology to support health and social care is acquiring an increasing global significance. Provision is framed within the context of a rapidly changing population profile, which is impacting on the number of people requiring health and social care, workforce availability and the funding of healthcare systems. Objectives To explore the effectiveness of smart home technologies as an intervention for people with physical disability, cognitive impairment or learning disability, who are living at home, and to consider the impact on the individual's health status and on the financial resources of health care. Search strategy We searched the following databases for primary studies: ( a) the Cochrane Effective Practice and Organisation of Care ( EPOC) Group Register, (b) the Cochrane Central Register of Controlled Trials (CENTRAL), (The Cochrane Library, issue 1, 2007), and (c) bibliographic databases, including MEDLINE (1966 to March 2007), EMBASE ( 1980 to March 2007) and CINAHL ( 1982 to March 2007). We also searched the Database of Abstracts of Reviews of Effectiveness ( DARE). We searched the electronic databases using a strategy developed by the EPOC Trials Search Co-ordinator. Selection criteria We included randomised controlled trials (RCTs), quasi-experimental studies, controlled before and after studies (CBAs) and interrupted time series analyses ( ITS). Participants included adults over the age of 18, living in their home in a community setting. Participants with a physical disability, dementia or a learning disability were included. The included interventions were social alarms, electronic assistive devices, telecare social alert platforms, environmental control systems, automated home environments and 'ubiquitous homes'. Outcome measures included any objective measure that records an impact on a participant's quality of life, healthcare professional workload, economic outcomes, costs to healthcare provider or costs to participant. We included measures of service satisfaction, device satisfaction and healthcare professional attitudes or satisfaction. Data collection and analysis One review author completed the search strategy with the support of a life and health sciences librarian. Two review authors independently screened titles and abstracts of results. Main results No studies were identified which met the inclusion criteria. Authors' conclusions This review highlights the current lack of empirical evidence to support or refute the use of smart home technologies within health and social care, which is significant for practitioners and healthcare consumers. | Literature review; International; MEDIUM |
| Mason A, Weatherly H, Spilsbury K, Arksey H, Golder S, Adamson J, Drummond M and Glendinning C | 2007 | A systematic review of the effectiveness and cost-effectiveness of different models of community-based respite care for frail older people and their carers | Objectives: To review the evidence for different models of community-based respite care for frail older people and their carers, where the participant group included older people with frailty, disability, cancer or dementia. Where data permitted, subgroups of carers and care recipients, for whom respite care is particularly effective or cost-effective, were to be identified. Data sources: Major databases were searched from 1980 to March 2005. Ongoing and recently completed research databases were searched in July 2005. Review methods: Data from relevant studies were extracted and quality assessed. The possible effects of study quality on the effectiveness data and review findings were discussed. Where sufficient clinically and statistically similar data were available, data were pooled using appropriate statistical techniques. Results: Twenty-two primary studies were included. Most of the evidence came from North America, with a minority of effectiveness and economic studies based in the UK. Types of service studied included day care, host family, in-home, institutional and video respite. Effectiveness evidence suggests that the consequences of respite upon carers and care recipients are generally small, with better controlled studies finding modest benefits only for certain subgroups. However, many studies report high levels of carer satisfaction. No reliable evidence was found that respite can delay entry to residential care or that respite adversely affects care recipients. Randomisation validity in the included randomised studies was sometimes unclear. Studies reported many different outcome measures, and all of the quasi-experimental and uncontrolled studies had methodological weaknesses. The descriptions of the studies did not provide sufficient detail of the methods of data collection or analysis, and the studies failed to describe adequately the groups of study participants. In some studies, only evidence to support respite care services was presented, rather than a balanced view of the services. Only five economic evaluations of respite care services were found, all of which compared day care with usual care and only one study was undertaken in the UK. Day care tended to be associated with higher costs and either similar or a slight increase in benefits, relative to usual care. The economic evaluations were based on two randomised and three quasi-experimental studies, all of which were included in the effectiveness analysis. The majority of studies assessed health and social service use and cost, but inadequate reporting limits the potential for exploring applicability to the UK setting. No study included generic health-related quality of life measures, making cost-effectiveness comparisons with other healthcare programmes difficult. One study used sensitivity analysis to explore the robustness of the findings. Conclusions: The literature review provides some evidence that respite for carers of frail elderly people may have a small positive effect upon carers in terms of burden and mental or physical health. Carers were generally very satisfied with respite. No reliable evidence was found that respite either benefits or adversely affects care recipients, or that it delays entry to residential care. Economic evidence suggests that day care is at least as costly as usual care. Pilot studies are needed to inform full-scale studies of respite in the UK. | Literature review; International; HIGH |
| Mavall L and Malmberg B | 2007 | Day care for persons with dementia: An alternative for whom? | Day care, as a type of care in between residential care and home help, has been available for several years, and is often referred to as an adequate alternative form of care for people with dementia. The goals of Swedish day care are to create a meaningful day for participants, offer family caregivers respite, and provide care for persons with dementia. The aim of this Swedish study was to describe day care clients with dementia problems over a 12-month period, and to discuss what distinguished those who discontinued day care from those who stayed with it. Of the clients, 76 percent were diagnosed by a doctor as having dementia between level one and four out of a total of six levels, according to the Berger rating scale of severity of senility. The results showed that one third of the people with dementia discontinued within four months. Another third dropped out within 12 months. People with behavioural problems and those who needed assistance with dressing and toileting discontinued earliest. All caregivers found some benefit of day care for their relatives and for themselves, with the exception of some caregivers of those who ended within four months. Most of the clients who lived alone at the start of day care, and ended within 12 months, went to a nursing home. Two differing conclusions may be drawn from the findings: (1) that offering day care services to persons with dementia who also show signs of behavioural problems is questionable; or (2) that the planning of day care in Sweden should be adjusted to also meet the needs of persons with behavioural problems, such as depression. In its current form, day care in Sweden seems only partially to fulfil its goals. | Qualitative study; Sweden; HIGH |
| McCrae N and Banerjee S | 2011 | Modernizing mental health services for older people: a case study | Background: This paper describes an evaluation of a redevelopment program in a mental health service for older people, stimulated by U. K. Department of Health policy. IQCOL (Improving Quality of Care for Older People in Lambeth) was a two-year program to modernize and expand an inner-city service, with objectives to improve access, embed new functions, and tune the service towards the needs of the local community. The program evaluation aimed to contribute to knowledge on service planning and methodology for evaluating complex interventions. Methods: The study evaluated the progress and outcomes of this multifaceted program. The realist model of evaluation was followed, with a dual emphasis on utility and generalizability. With an iterative approach, the pragmatic, longitudinal design comprised a combination of qualitative and quantitative methods to explain the process of change and to measure achievement of objectives. Results: A high level of participation in evaluation activities was achieved. The workforce generally responded well to the program. However, progress in one team was hindered by understaffing and resistance to change, emphasizing that while localized provision may be desirable, team viability requires adequate resources and professional support. Improved access was indicated by a 13% increase of referrals. Data suggested earlier referral of dementia cases. Carer support was implemented, but assertive outreach was impeded by professional boundary issues. Ethnicity data showed that the service was responding to demographic trends. Positive views towards the program were associated with team resources and recent professional training. Conclusions: This case study demonstrates how whole system change can be achieved if sufficient attention is given to the needs of staff implementing the program. The evaluation emphasizes the importance of context in producing generalizable evidence on service development, and contributes useful methodological insights. | Qualitative study; UK; MEDIUM |
| McGilton K, Boscart V, Fox M, Sidani S, Rochon E and Sorin-Peters R | 2009 | A systematic review of the effectiveness of communication interventions for health care providers caring for patients in residential care settings | Objectives: This systematic review will describe the theoretical grounding, components, duration, mode of delivery, and outcomes of communication interventions for health care providers delivering care in residential care settings and will evaluate the effectiveness of these interventions. Methods: We conducted a comprehensive literature search of multiple databases published from January 1985 to the first week of December 2007, supplemented by a hand search of the references in all relevant articles, to find studies that met the inclusion criteria. Intervention details were extracted, and the studies' validity was evaluated independently by two researchers using a standardized data collection form based on Cooper and Hedges' (1994) approach to quality assessment. Results: Of the six studies that met the inclusion criteria (three randomized controlled trials, three quasi-experimental designs), three used a theoretical framework to guide intervention design. Across the six studies, the most commonly used components were (1) cognitive (to teach staff about communication), (2) behavioural (including practice at the bedside), and (3) psychological (involving individualized feedback). Despite the studies' variability in methodological quality, their results indicated that communication interventions have a positive effect on staffs' knowledge and communication skills and on residents' agitation and challenging behaviours. However, none of the studies provided sufficient information on the duration of the intervention and on determining which interventions were most effective. This made it difficult to draw conclusions about the effectiveness of the interventions' different components. Conclusion: Although communication training has been shown to have positive effects on staffs' communication knowledge and skills as well as on resident outcomes, future controlled intervention research is needed to assess the effectiveness of individual intervention components. | Literature review; International; MEDIUM |
| Metha S, Siegler E, Henderson C and Carrington Reid M | 2010 | Acute pain management in hospitalized patients with cognitive impairment: a study of provider practices and treatment outcomes. | Background. Despite new guidelines and nationally mandated regular assessments, managing pain in cognitively impaired patients remains a complex and challenging task. Numerous studies have focused on assessing pain in this population; however, studies of treatment are limited. Purpose. The purpose of this article was to characterize assessment and pain management strategies used by providers caring for hospitalized cognitively impaired patients with acute pain, and to assess for associations between amount of opioid received and specific adverse outcomes in this patient population. Methods. Medical records of patients admitted to the Geriatrics Service or Orthopedic Service or evaluated by the Geriatrics Consult Service at an urban tertiary care hospital between September 01, 2006 and September 30, 2007 with cognitive impairment and an acute pain problem on admission were reviewed. Results. Participants (N = 100) had a mean age of 86 years (range = 68-99), and were mostly female (83%) with fracture-related pain (62%). A numeric pain score was recorded in 67% of nursing assessments vs < 5% of physician assessments. Opioids were prescribed for 100% of the surgical patients vs 43% of the medical patients. Only 15% of patients were placed on a standing analgesic regimen. Nonpharmacological management was employed for 75% of surgical patients vs 43% of medical patients. Delirium occurred in 27% of patients, and 33% experienced an interruption of physical therapy. Neither, however, was associated with level of opioid use. Conclusions. Current assessment and treatment practices in acute pain management for cognitively impaired patients vary widely (to include service and provider type). Implementation of evidence-based guidelines is needed to improve patient care. | Cohort study; USA; MEDIUM |
| Millard F | 2009 | Dementia - who cares?: A comparison of community needs and primary care services | Background Health professionals have varying levels of knowledge about, and interest in, providing dementia services. This article compares patient experiences in dealing with dementia with the perceived role of health care providers in providing dementia care. Method Qualitative data from interviews of patients with dementia and their carers was compared with quantitative data from health professional surveys, where health professionals described their knowledge of dementia and their attitudes toward, and roles in, management. Results Patients often notice dementia symptoms before their general practitioner and seek diagnosis and support, Not all GPs wish to provide dementia services and many are unaware of the benefits; of early diagnosis and dementia care guidelines. Dementia forums attract older health professionals, suggesting younger members are less engaged in dementia care. Older patients tend to consult with older GPs, but older GPs are less aware of dementia diagnosis and management guidelines. Discussion Patients turn to their GP for help with dementia but may find most benefit from the assistance and advice of people who have already negotiated the pathways to care. Health professionals who fail to investigate patients presenting with dementia symptoms can delay diagnosis, denying patients and carers early intervention that could improve quality of life for both patient and carer. | Qualitative study; Australia; LOW |
| Milne A | 2010 | Dementia screening and early diagnosis: The case for and against | Over 700,000 people have dementia in the UK. There is increasing policy and practice consensus that early intervention in identifying and treating dementia is beneficial and that much can be done therapeutically and practically to help users and their relatives at an early stage. Research evidences early diagnosis as allowing users the chance to come to terms with it when they can still understand its implications. It also provides an opportunity for key decisions to be made and is what the majority of people want. However, early diagnosis also carries risks: loss of status, acquisition of a stigmatising label, loss of employment and, for a minority, depression. Not all users want to know they have dementia; the diagnosis may also be incorrect. Evidence from the field of medical sociology offers a different perspective on the early intervention debate suggesting not only that targeting older people at possible risk of dementia may be the latest product of surveillance medicine but that efforts to resist being diagnosed may represent strategies to challenge medical intrusion, knowledge and power. Further, the imposition by an older person and their family of their individualised values onto the clinical encounter may be viewed as an attempt to import user generated forms of knowledge into a medically managed process and provide a nuanced approach to defining, and dealing with, risks. Accommodating both perspectives in practice development may hold considerable potential to enhance the nature of care and the quality of lives of people with dementia and their families. | Literature review; International; MEDIUM |
| Moniz Cook E, Swift K, James I, Malouf R, De Vugt M and Verhey F | 2012 | Functional analysis-based interventions for challenging behaviour in dementia | Background Functional analysis (FA) for the management of challenging behaviour is a promising behavioural intervention that involves exploring the meaning or purpose of an individual's behaviour. It extends the 'ABC' approach of behavioural analysis, to overcome the restriction of having to derive a single explanatory hypothesis for the person's behaviour. It is seen as a first line alternative to traditional pharmacological management for agitation and aggression. FA typically requires the therapist to develop and evaluate hypotheses-driven strategies that aid family and staff caregivers to reduce or resolve a person's distress and its associated behavioural manifestations. Objectives To assess the effects of functional analysis-based interventions for people with dementia (and their caregivers) living in their own home or in other settings. Search methods We searched ALOIS: the Cochrane Dementia and Cognitive Improvement Group's Specialized Register on 3 March 2011 using the terms: FA, behaviour (intervention, management, modification), BPSD, psychosocial and Dementia. Selection criteria Randomised controlled trials (RCTs) with reported behavioural outcomes that could be associated with functional analysis for the management of challenging behaviour in dementia. Data collection and analysis Four reviewers selected trials for inclusion. Two reviewers worked independently to extract data and assess trial quality, including bias. Meta-analyses for reported incidence, frequency, severity of care recipient challenging behaviour and mood (primary outcomes) and caregiver reaction, burden and mood were performed. Details of adverse effects were noted. Main results Eighteen trials are included in the review. The majority were in family care settings. For fourteen studies, FA was just one aspect of a broad multi-component programme of care. Assessing the effect of FA was compromised by ill-defined protocols for the duration of component parts of these programmes (i.e. frequency of the intervention or actual time spent). Therefore, establishing the real effect of the FA component was not possible. Overall, positive effects were noted at post-intervention for the frequency of reported challenging behaviour (but not for incidence or severity) and for caregiver reaction (but not burden or depression). These effects were not seen at follow-up. Authors' conclusions The delivery of FA has been incorporated within wide ranging multi-component programmes and study designs have varied according to setting - i.e. family care, care homes and hospital, with surprisingly few studies located in care homes. Our findings suggest potential beneficial effects of multi-component interventions, which utilise FA. Whilst functional analysis for challenging behaviour in dementia care shows promise, it is too early to draw conclusions about its efficacy. | Literature review; International; HIGH |
| Moniz-Cook E | 2010 | Early psychosocial interventions in dementia: translating evidence into practice | *(No abstract on original paper – summary provided by current authors).* This is a very brief article which gives an overview of selected evidence of the benefits of psychosocial interventions early in dementia. It mentions a growing evidence base in support of group cognitive stimulation therapy, cognitive rehabilitation, and interventions to support mood and wellbeing for people with dementia. It suggests that the best effects have been found for psychotherapeutic interventions which involve both dementia sufferers and carers together, both in those interventions designed to reduce existing distress and those which aim to prevent future distress. Finally, it notes the challenges to wide-scale implementation of evidence-based practice. | Other (Commentary); Not specified; LOW |
| Monsch AU and Kressig RW | 2010 | Specific care program for the older adults: Memory clinics | Memory Clinics are interdisciplinary, specialized outpatient units for the early diagnosis of cognitive impairments and rare forms of dementia in the rapidly growing older population. At the Basel Memory Clinic, a team of neuropsychologists, geriatricians, neurologists, and psychiatrists use standardized procedures to comprehensively diagnose patients in the earliest possible stages of dementia or mild cognitive impairment. In collaborative efforts with family physicians and dementia care providing agencies, such as the Alzheimer's Association, patients and families are offered up-to-date pharmacologic and non-pharmacologic treatment options and educational and counseling services. These efforts aim to maintain the quality of life of both patients and caregivers as long as possible and to delay nursing home placement which accounts for the majority of costs associated with dementia. | Other (Service description); Switzerland; MEDIUM |
| Morano CL and King DM | 2010 | Lessons learned from implementing a psycho-educational intervention for African American dementia caregivers | The process of developing and implementing a psycho-educational intervention for African American caregivers in the United States is described. The process which was informed by a review of the care giving and intervention literature, and stress, appraisal, and coping theory. The intervention included 12 modules that lasted approximately 90 minutes each. Twelve participants were recruited from the local Association, local area churches and social clubs, and primary care physicians. The article discusses issues that came up during the pilot test and highlights the importance of participant input, in the design, implementation, and evaluation of intervention programmes. The dilemmas created due to the competing needs for good research with those of the participants are highlighted. | Qualitative study; USA; MEDIUM |
| Morgan DG, Crossley M, Kirk A, D’Arcy C, Stewart N, Biem J, Forbes D, Harder S, Basran J, Bello-Haas VD and McBain L | 2008 | Improving access to dementia care: Development and evaluation of a rural and remote memory clinic | The availability, accessibility and acceptability of services are critical factors in rural health service delivery. In Canada, the aging population and the consequent increase in prevalence of dementia challenge the ability of many rural communities to provide specialized dementia care. This paper describes the development, operation and evaluation of an interdisciplinary memory clinic designed to improve access to diagnosis and management of early stage dementia for older persons living in rural and remote areas in the Canadian province of Saskatchewan. We describe the clinic structure, processes and clinical assessment, as well as the evaluation research design and instruments. Finally, we report the demographic characteristics and geographic distribution of individuals referred during the first three years. Adapted from the source document. | Qualitative study; Canada; MEDIUM |
| Morhardt D | 2011 | Accessing community-based and long-term care services: Challenges facing persons with Frontotemporal dementia and their families | There are several barriers to accessing services for persons with frontotemporal dementia (FTD), and few studies have examined service needs and satisfaction with services for family caregivers of persons with FTD. Persons with FTD and their families have reported consistent difficulties in their attempts to access care and support. These are: (1) difficulty obtaining a diagnosis; (2) financial concerns due to loss of employment, job-related income; (3) problems accessing social security disability insurance; and (4) lack of adequate community-based and long-term care services and resources. Successful care practices such as use of an interdisciplinary team and helpful care models such as person-centred care and the antecedent-behaviour-consequence method are described. Further investigation and research are needed to understand best care strategies for persons with FTD. | Other (Practice-based reflection); USA; HIGH |
| Moriarty J, Sharif N and Robinson J | 2011 | Black and minority ethnic people with dementia and their access to support and services | No abstract. Key messages: • An increase in the number of older black and minority ethnic (BME) people in the UK is likely to lead to an increased need for dementia services. • Lower levels of awareness about dementia and the existence of stigma within BME communities help explain why BME people are currently under-represented in dementia services. • However, staff can adopt several approaches to improving the uptake of services, such as developing different information resources and appointing workers with responsibility for outreach. • Staff working in dementia services would like more training on how to give culturally acceptable care and support to BME people with dementia. • Carers of BME people with dementia may feel reluctant to ask for help, although support in the form of carers’ groups and respite services may be appreciated. Different communities may have differing views about whether they wish these services to be culturally specific or mixed. • The current UK evidence base on supporting BME people with dementia and their carers is very limited and reliant upon a small number of local studies. | Literature review; International; HIGH |
| Mowszowski L, Batchelor J and Naismith SL | 2010 | Early intervention for cognitive decline: can cognitive training be used as a selective prevention technique? | Background: Cognitive training (CT) may be effective as a therapeutic strategy to prevent cognitive decline in older adults. This review evaluates CT as a preventive tool at various stages of a prevention hierarchy with specific reference to healthy older adults, "at risk" and clinical populations. It also considers the underlying mechanism of CT, namely that which suggests that CT acts via promoting neuroplasticity. Methods: Evidence for CT in healthy, "at risk" and clinical populations has been systematically reviewed elsewhere. This review re-examines several studies in each group to clarify the potential of CT as a preventive technique, with a key focus on the secondary level of prevention. Results: Studies in healthy older adults and those with mild cognitive impairment are largely positive and suggest that CT has the potential to improve cognition. However, findings in relation to Alzheimer's disease are mixed. Limitations of existing research include diverse methodologies and CT programs, small samples, insufficient focus on functional outcomes, sustainability and generalization of effects and the need for imaging data to delineate mechanisms of change. Additionally, there is limited data on those with late-life depression, despite this being an independent risk factor for dementia. Conclusions: CT offers promise as a preventive therapeutic technique in healthy older adults and particularly as a secondary prevention method for "at risk" groups. Future investigations need to focus on methodological constraints and delineating possible neuroplastic mechanisms of action. Nonetheless, CT programs may represent a viable, non-pharmacological early intervention strategy, as they are easily-implemented, engaging and promote social interaction in group settings. | Literature review; International; MEDIUM |
| Nejtek VA, Hardy S, Hall JR and Winter AS | 2011 | Characteristics and psychosocial predictors of psychiatric emergency centre transport and length of stay in patients with dementia and Alzheimer’s Disease: A preliminary report | Background. Agitation and aggression are common behaviours that often lead to psychiatric emergency centre (PEC) admission of nursing home patients with dementia or Alzheimer's disease. However, few if any data are available that adequately describe characteristics and psychosocial triggers of agitation and aggression leading to transport and admission to a PEC. Methods. A preliminary investigation to explore all possible characteristics and psychosocial predictors of PEC transport and length of stay in men and women nursing home patients with dementia or Alzheimer's disease was designed. Frequency distributions, chi-square, analyses of variance, and regression analyses were used to analyze the data. Results. One hundred PEC patient charts were reviewed, of which 58 charts were missing information and 42 charts provided evaluable data. Nursing homes located in impoverished areas transported patients to the PEC significantly more often than those in affluent areas. A disconnect between the agitated/aggressive mental state reported by nursing home staff leading to the PEC transport and the calm/cooperative mental status PEC clinicians observed during the admission process was evident. Data from the charts also showed that 74% of patients received off-label antipsychotics rather than FDA-approved medications to treat dementia or Alzheimer's disease. Conclusions. This is one of the few studies to identify characteristics and psychosocial triggers of PEC use and length of stay in nursing home patients. We also highlight potentially dangerous antipsychotic use in dementia and Alzheimer disease. Thus, our data add to the existing knowledge base regarding PEC utilization, length of stay, and pharmacotherapy in nursing home patients with dementia and Alzheimer's disease. Given the preliminary nature of this study, however, the results should be interpreted with caution. | Other (Retrospective case review); USA; MEDIUM |
| Neville CC and Byrne GJA | 2006 | The impact of residential respite care on the behaviour of older people with dementia: literature review | Aim: The aim of this review was to examine the impact of residential respite care on the behaviour displayed by older people with dementia. Background: Relocation of older people with dementia to a different care setting could have a negative impact on their behaviour. If such a response is anticipated, a short-term admission to a residential aged care facility for respite care would appear to be futile for the older person and their home caregiver. Therefore, it is important to know what the outcomes of residential respite care are in relation to behaviour for older people with dementia. Methods: A literature search was undertaken and papers emerged from a range of disciplines. The search terms 'respite'; 'respite care'; 'residential respite care'; 'short-stay', 'short-term', 'overnight stays/admissions', 'behave*' and various combinations of these terms were used to find relevant publications in English from Ageline, CINAHL, Medline, and Psychinfo databases dating from 1966. Other key publications were located when searching through the reference lists of retrieved publications. A limited body of literature on residential respite care for older people with dementia was revealed. Results: Six studies were identified, which met the criteria of residential respite care as the intervention and behaviour as an outcome. Conclusions: The studies had a variety of methodological limitations and produced contradictory findings. The strength of the studies was the reporting of outcomes for respite recipients who are usually overlooked in the outcomes for respite research. Relevance to clinical practice: Residential respite care has great anecdotal support but more empirical and evaluative research is needed on outcomes for the respite recipients. | Literature review; International; MEDIUM |
| Olazarán J, Reisberg B, Clare L, Cruz I, Peña-Casanova J, del Ser T, Woods B, Beck C, Auer S, Lai C, Spector A, Fazio S, Bond J, Kivipelto M, Brodaty H, Rojo JM, Collins H, Teri L, Mittelman M, Orrell M, Feldman HH and Muñiz R | 2010 | Nonpharmacological therapies in Alzheimer’s Disease: A systematic review of efficacy | Introduction: Nonpharmacological therapies (NPTs) can improve the quality of life (QoL) of people with Alzheimer's disease (AD) and their carers. The objective of this study was to evaluate the best evidence on the effects of NPTs in AD and related disorders (ADRD) by performing a systematic review and meta-analysis of the entire field. Methods: Existing reviews and major electronic databases were searched for randomized controlled trials (RCTs). The deadline for study inclusion was September 15, 2008. Intervention categories and outcome domains were predefined by consensus. Two researchers working together detected 1,313 candidate studies of which 179 RCTs belonging to 26 intervention categories were selected. Cognitive deterioration had to be documented in all participants, and degenerative etiology indicating dementia) had to be present or presumed in at least 80% of the subjects. Evidence tables, meta-analysis and summaries of results were elaborated by the first author and reviewed by author subgroups. Methods for rating level of evidence and grading practice recommendations were adapted from the Oxford Centre for Evidence-Based Medicine. Results: Grade A treatment recommendation was achieved for institutionalization delay (multicomponent interventions for the caregiver, CG). Grade B recommendation was reached for the person with dementia (PWD) for: improvement in cognition (cognitive training, cognitive stimulation, multicomponent interventions for the PWD); activities of daily living (ADL) (ADL training, multicomponent interventions for the PWD); behaviour (cognitive stimulation, multicomponent interventions for the PWD, behavioural interventions, professional CG training); mood (multicomponent interventions for the PWD); QoL (multicomponent interventions for PWD and CG) and restraint prevention (professional CG training); for the CG, grade B was also reached for: CG mood (CG education, CG support, multicomponent interventions for the CG); CG psychological well-being (cognitive stimulation, multicomponent interventions for the CG); CG QoL (multicomponent interventions for PWD and CG). Conclusion: NPTs emerge as a useful, versatile and potentially cost-effective approach to improve outcomes and QoL in ADRD for both the PWD and CG. | Literature review; International; HIGH |
| Parker D, Mills S and Abbey J | 2008 | Effectiveness of interventions that assist caregivers to support people with dementia living in the community: a systematic review | Objectives The objective of this review was to assess the effectiveness of interventions that assist caregivers to provide support for people living with dementia in the community. Inclusion criteria Types of participants Adult caregivers who provide support for people with dementia living in the community (non-institutional care). Types of interventions Interventions designed to support caregivers in their role such as skills training, education to assist in caring for a person living with dementia and support groups/programs. Interventions of formal approaches to care designed to support caregivers in their role, care planning, case management and specially designated members of the healthcare team - for example dementia nurse specialist or volunteers trained in caring for someone with dementia. Types of studies This review considered any meta-analyses, systematic reviews, randomised control trials, quasi-experimental studies, cohort studies, case control studies and observational studies without control groups that addressed the effectiveness of interventions that assist caregivers to provide support for people living with dementia in the community. Search strategy The search sought to identify published studies from 2000 to 2005 through the use of electronic databases. Only studies in English were considered for inclusion. The initial search was conducted of the databases, CINAHL, MEDLINE and PsychINFO using search strategies adapted from the Cochrane Dementia and Cognitive Improvement Group. A second more extensive search was then conducted using the appropriate Medical Subject Headings (MeSH) and keywords for other available databases. Finally, hand searching of reference lists of articles retrieved and of core dementia, geriatric and psycho geriatric journals was undertaken. Assessment of quality Methodological quality of each of the articles was assessed by two independent reviewers using appraisal checklist developed by the Joanna Briggs Institute and based on the work of the Cochrane Collaboration and Centre for Reviews and Dissemination. Data collection and analysis Standardised mean differences or weighted mean differences and their 95% confidence intervals were calculated for each included study reported in the meta-analysis. Results from comparable groups of studies were pooled in statistical meta-analysis using Review Manager Software from the Cochrane Collaboration. Heterogeneity between combined studies was tested using standard chi-square test. Where statistical pooling was not appropriate or possible, the findings are summarised in narrative form. Results A comprehensive search of relevant databases, hand searching and cross referencing found 685 articles that were assessed for relevance to the review. Eighty-five papers appeared to meet the inclusion criteria based on title and abstract, and the full paper was retrieved. Of the 85 full papers reviewed, 40 were accepted for inclusion, three were systematic reviews, three were meta-analysis, and the remaining 34 were randomised controlled trials. For the randomised controlled trials that were able to be included in a meta-analysis, standardised mean differences or weighted mean differences and their 95% confidence intervals were calculated for each. Results from comparable groups of studies were pooled in statistical meta-analysis using Review Manager Software and heterogeneity between combined studies was assessed by using the chi-square test. Where statistical pooling was not appropriate or possible, the findings are summarised in narrative form. The results are discussed in two main sections. Firstly it was possible to assess the effectiveness of different types of caregiver interventions on the outcome categories of depression, health, subjective well-being, self-efficacy and burden. Secondly, results are reported by main outcome category. For each of these sections, meta-analysis was conducted where it was possible; otherwise, a narrative summary describes the findings. Effectiveness of intervention type Four categories of intervention were included in the review - psycho-educational, support, multi-component and other. Psycho-educational Thirteen studies used psycho-educational interventions, and all but one showed positive results across a range of outcomes. Eight studies were entered in a meta-analysis. No significant impact of psycho-educational interventions was found for the outcome categories of subjective well-being, self-efficacy or health. However, small but significant results were found for the categories of depression and burden. Support Seven studies discussed support only interventions and two of these showed significant results. These two studies were suitable for meta-analysis and demonstrated a small but significant improvement on caregiver burden. Multi-component Twelve of the studies report multi-component interventions and 10 of these report significant outcomes across a broad range of outcome measures including self-efficacy, depression, subjective well-being and burden. Unfortunately because of the heterogeneity of study designs and outcome measures, no meta-analysis was possible. Other interventions Other interventions included the use of exercise or nutrition which resulted in improvements in psychological distress and health benefits. Case management and a computer aided support intervention provided mixed results. One cognitive behavioural therapy study reported a reduction in anxiety and positive impacts on patient behaviour. Effectiveness of interventions using specific outcome categories In addition to analysis by type of intervention it was possible to analyse results based on some outcome categories that were used across the studies. In particular the impact of interventions on caregiver depression was available for meta-analysis from eight studies. This indicated that multi-component and psycho-educational interventions showed a small but significant positive effect on caregiver depression. Five studies using the outcome category of caregiver burden were entered into a meta-analysis and findings indicated that there were no significant effects of any of interventions. No meta-analysis was possible for the outcome categories of health, self-efficacy or subjective well-being. Implications for practice From this review there is evidence to support the use of well-designed psycho-educational or multi-component interventions for caregivers of people with dementia who live in the community. Factors that appear to positively contribute to effective interventions are those which: Provide opportunities within the intervention for the person with dementia as well as the caregiver to be involved Encourage active participation in educational interventions for caregivers Offer individualised programs rather than group sessions Provide information on an ongoing basis, with specific information about services and coaching regarding their new role Target the care recipient particularly by reduction in behaviours Factors which do not appear to have benefit in interventions are those which: Simply refer caregivers to support groups Only provide self help materials Only offer peer support. | Literature review; International; HIGH |
| Perry M, Drašković I, Lucassen P, Vernooij-Dassen M, van Achterberg T and Rikkert MO | 2010 | Effects of educational interventions on primary dementia care: A systematic review | Background: Early diagnosis of dementia benefits both patient and caregiver. Nevertheless, dementia in primary care is currently under-diagnosed. Some educational interventions developed to improve dementia diagnosis and management were successful in increasing the number of dementia diagnoses and in changing attitudes and knowledge of health care staff. However, none of these interventions focussed on collaboration between GPs and nurses in dementia care. We developed an EASY care-based Dementia Training Program ( DTP) aimed at stimulating collaboration in dementia primary care. We expect this program to increase the number of cognitive assessments and dementia diagnoses and to improve attitudes and knowledge of GPs and nurses. Methods: The DTP is a complex educational intervention that consists of two workshops, a coaching program, access to an internet forum, and a Computerized Clinical Decision Support System on dementia diagnostics. One hundred duos of GPs and nurses will be recruited, from which 2/3 will be allocated to the intervention group and 1/3 to the control group. The effects of implementation of the DTP will be studied in a cluster-randomised controlled trial. Primary outcomes will be the number of cognitive assessments and dementia diagnoses in a period of 9 months following workshop participation. Secondary outcomes are measured on GP and nurse level: adherence to national guidelines for dementia, attitude, confidence and knowledge regarding dementia diagnosis and management; on patient level: number of emergency calls, visits and consultations and patient satisfaction; and on caregiver level: informal caregiver burden and satisfaction. Data will be collected from GPs' electronic medical records, self-registration forms and questionnaires. Statistical analysis will be performed using the MANOVA-method. Also, exploratory analyses will be performed, in order to gain insight into barriers and facilitators for implementation and the possible causal relations between the rate of success of the intervention components and the outcomes. Discussion: We developed multifaceted dementia training programme. Novelties in this programme are the training in fixed collaborative duos and the inclusion of an individual coaching program. The intervention is designed according to international guidelines and educational standards. Exploratory analysis will reveal its successful elements. Selection bias and contamination may be threats to the reliability of future results of this trial. Nevertheless, the results of this trial may provide useful information for policy makers and developers of continuing medical education. Trial registration: ClinicalTrials.gov ID NCT00459784. | Literature review; International; HIGH |
| Pimouguet C, Lavaud T, Dartigues JF and Helmer C | 2010 | Dementia case management effectiveness on health care costs and resource utilization: a systematic review of randomized controlled trials | The growing number of dementia patients leads to both policy, economic and health organization constraints. Many healthcare systems have developed case management programs in order to optimize dementia patients and caregivers care and services delivery. Nevertheless, to what extend case management programs can lead to an improvement of care and expenditures savings is not known. Thus, the objective of this paper was to analyse the efficacy of case management programs on health care cost, institutionalization and hospitalization. A systematic review of randomized controlled trials was therefore conducted of the databases MEDLINE and SCOPUS up to September 2009. Included were English language randomized controlled trials of case management for community dwelling dementia patients and their caregivers evaluating costs, institutionalization and hospitalization. An evaluation of the methodological quality was performed. Thirteen relevant studies concerning 12 trials were identified and included. None of the 7 low quality studies reported positive impact of case management on the outcomes of interest. Among the 6 good quality studies, 4 reported positive impact on institutionalization delay, institutionalization length or nursing home admission rate. In none of the good quality studies was evidence found for savings in health care expenditures or reduction in hospitalization recourse. The weak convincing evidences from randomized trials do not allow any conclusion about the efficacy of case management for dementia patient and caregivers on costs and resource utilization. Further research should focus on determining subgroups of caregivers who could benefit the most from case management. | Economic evaluation; International; MEDIUM |
| Pitfield C, Shahriyarmolki K and Livingston G | 2011 | A systematic review of stress in staff caring for people with dementia living in 24-hour care settings | Background: Family carers of people with dementia are at risk of psychological morbidity, and it is suggested that this may also be the case in paid carers as caring for people with dementia can be emotionally and physically demanding. Care homes have historically had difficulty recruiting and retaining staff, and job stress has previously been linked to high turnover amongst long-term care staff. We performed a systematic review of studies of the prevalence of psychological stress in staff caring for people with dementia in residential long-term care settings. Methods: We conducted a comprehensive literature search of MEDLINE, PsychINFO and Web of Science databases up to May 2009, supplemented by a search of the references of all relevant articles. Search terms encompassed nursing staff, residential care and psychological distress. Validity of studies was graded by two authors independently using a standardized checklist. Results: We identified 601 studies of which five met our inclusion criteria. Two studies reported on prevalence rates of staff distress and found 37% and 5% levels of being "at risk" from burnout, four studies reported mean stress scores and all were low. Conclusions: All studies were either small or used instruments with unsatisfactory psychometric properties and so our conclusions are limited by the lack of good quality evidence. The preliminary evidence suggests that most staff who remain working in homes do not have a high prevalence of psychological stress or level of symptoms. | Literature review; International; MEDIUM |
| Ploeg J, Denton M, Tindale J, Hutchison B, Brazil K, Akhtar-Danesh N, Lillie J and Plenderleith J M | 2009 | Older Adults' Awareness of Community Health and Support Services for Dementia Care | The article examines where older adults seek help in caring for a parent with dementia and the factors associated with their identification of community health and support services as sources of assistance. The authors conducted telephone interviews, using random digit dialing, of 1,152 adults aged 50 and over in the city of Hamilton. Respondents received a vignette that raised issues related to parental dementia. In identifying support sources, over 37 per cent of respondents identified their physician, 33 per cent identified informal support such as family and neighbors, and 31 per cent identified home health services. Only 18 per cent identified community support services. Female participants having higher levels of education were more likely to identify their physician as a source of support. Knowing where to find information about community support services was associated with an increased likelihood of mentioning physicians and home health services as sources of assistance. | Other (Report of survey); Canada; HIGH |
| Preschl B, Wagner B, Forstmeier S and Maercker A | 2011 | E-Health Interventions for Depression, Anxiety Disorder, Dementia, and Other Disorders in Old Age: A Review | E-health interventions targeting older adults seem to be a promising approach in domains including depression, anxiety disorder, and dementia are three of the most prevalent mental disorders in old age. Further, these technical innovations (e.g., ambient-assisted living and smart homes, game-based applications and training programs) may have the potential to compensate for or prevent health-related changes or to foster active aging. As highlighted by this literature review, however, research in this area is still at an early stage. The methodological quality of the studies and projects differs, and there is a lack of randomized controlled trials and robust research designs (much research to date has been limited to pilot and short-term studies). Advantages and challenges of using information and communication technology (ICT) applications in the above-mentioned domains are discussed, as are user characteristics. | Literature review; International; LOW |
| Rothera I, Jones R, Harwood R, Avery AJ, Fisher K, James V, Shaw I and Waite J | 2008 | An evaluation of a specialist multiagency home support service for older people with dementia using qualitative methods | Background Standard home care support for people with dementia has been criticised in statutory inspection reports, and may lead to unnecessary crises, hospital or care home admissions. Objective To establish whether a specialist multi agency hot ne care service for older people with dementia delivered better quality care than standard services, and how any improvements were achieved. Design Qualitative study, using semi-structured interviews, focus groups and small group interviews. Setting Two demographically similar areas in Nottingham, one served by a specialist home care team, the other by standard services. Participants Twenty-seven service users, 18 family carers, 17 home care workers, 20 health/social care professionals, across both services. Results The specialist service demonstrated greater flexibility and responsiveness to the particular needs and circumstances of service users and family carers, who were encouraged to participate in routine decision-making and activities. By sharing responsibilities, the specialist service helped reduce carer stress and prevent crises. These outcomes depended on the configuration of the service, including multidisciplinary health and social services input, careworker autonomy and independence, continuous reassessment of clients' circumstances and preferences and the capacity to develop long-term relationships, through careworker continuity. The standard service, which used a task-orientated approach, lacked these characteristics. Conclusions This study provides evidence of the benefits of a specialist multiagency home support service over standard home care, in the opinion of service users, carers and careworkers, and defines the operational model that achieves this. Findings confirm best practice recommendations, based on models of dementia care which emphasise respect for 'personhood'. | Qualitative study; UK; HIGH |
| Ryan T, Gardiner C, Bellamy G, Gott M and Ingleton C | 2012 | Barriers and facilitators to the receipt of palliative care for people with dementia: the views of medical and nursing staff | Background: The global prevalence of dementia is set to rise to almost 65 million people by 2030, providing policy makers and practitioners with significant challenges, not least within the realms of end-of-life care. The international literature would suggest that people with dementia may benefit from palliative forms of care, but evidence indicates that many fail to access such provision at the end of life. The role of the health care team is pivotal if people with dementia are to benefit from the transition to palliative care. Aim: This paper reports on qualitative research conducted in the UK that sought to explore the experiences of health care practitioners working in palliative care and sought to establish the issues relating to end-of-life care for people with dementia. Design: Eight focus groups and four individual interviews were held. Data were analysed using a thematic approach. Setting/participants: The study included palliative care practitioners (n = 58) including medical, nursing and allied health professionals. Participants were recruited from acute hospitals, general practice, hospices and specialist palliative care units in the UK. Results: Four themes were identified: Making the transition; Competence challenged; 'The long view' and Working together. Whilst there exists good practice in this area, the barriers to timely and appropriate transitions to palliative care for people with dementia and their families continue to exist. The paper concludes with recommendations for policy and practice development. | Qualitative study; UK; HIGH |
| Sampson EL, Jones L, Thuné-Boyle ICV, Kukkastenvehmas R, King M, Leurent B, Tookman A and Blanchard MR | 2010 | Palliative assessment and advance care planning in severe dementia: An exploratory randomized controlled trial of a complex intervention | Patients with advanced dementia often receive poor end-of-life care. We aimed to design and pilot a palliative care and advance care plan (ACP) intervention. Patients had undergone emergency hospital admission and had severe dementia. The intervention consisted of a palliative care patient assessment which informed an ACP discussion with the carer, who was offered the opportunity to write an ACP for the person with dementia. Carer-patient dyads were randomized to 'usual care' or the intervention. Carer-related outcome measures included the Kessler Distress Scale, Decision Satisfaction Inventory, Client Satisfaction Questionnaire and the Euroqol-5D, measured at baseline, six weeks, six months and three months after bereavement. The Satisfaction with End of Life Care in Dementia Scale was completed if the patient died. The 32 patient participants were physically frail and in the advanced stages of dementia: 62% had pressure damage to the skin, all needed feeding assistance and 95% were in pain. Nearly 50% died during the six-month follow-up period. Carers were difficult to recruit during acute admission; 33 patients and carers entered the study (22 intervention arm; 11 control arm). Only seven carers made ACPs. The care planning discussion was well received, but few carers wrote an ACP, despite intensive support from an experienced nurse specialist. Advance care planning is, in theory, a necessary intervention for people with severe dementia; the reluctance of carers to write plans needs to be explored further. | Randomised Controlled Trial (RCT); UK; MEDIUM |
| Sampson L, Harrison-Dening K, Greenish W, Mandal U, Holman A and Jones L | 2009 | End of life care for people with dementia | This project aimed to identify the major barriers for people with dementia and their carers in accessing good quality end of life care and suggest cost effective ways of enabling improvements based on review findings. The methodology included an audit of a small sample of nine case notes, interviews with a small cohort of seven carers of people with dementia who had recently died and a series of focus group meetings and interviews with professionals. Recommendations are identified in the areas of: pathways of care, the impact of hospitalisation, financial implications, advance care planning, impact on carers, and skills and training. | Qualitative study; UK; HIGH |
| Smith ER, Broughton M, Baker R, Pachana NA, Angwin AJ, Humphreys MS, Mitchell L, Bryne GJ, Copland D A, Gallois C, Hegney D and Chenery H J | 2011 | Memory and communication support in dementia: research-based strategies for caregivers. | Background: Difficulties with memory and communication are prominent and distressing features of dementia which impact on the person with dementia and contribute to caregiver stress and burden. There is a need to provide caregivers with strategies to support and maximize memory and communication abilities in people with dementia. In this project, a team of clinicians, researchers and educators in neuropsychology, psychogeriatrics, nursing and speech pathology translated research-based knowledge from these fields into a program of practical strategies for everyday use by family and professional caregivers. Methods: From the available research evidence, the project team identified compensatory or facilitative strategies to assist with common areas of difficulty, and structured these under the mnemonics RECAPS (for memory) and MESSAGE (for communication). This information was adapted for presentation in a DVD-based education program in accordance with known characteristics of effective caregiver education. Results: The resultant DVD comprises (1) information on the nature and importance of memory and communication in everyday life; (2) explanations of common patterns of difficulty and preserved ability in memory and communication across the stages of dementia; (3) acted vignettes demonstrating the strategies, based on authentic samples of speech in dementia; and (4) scenarios to prompt the viewer to consider the benefits of using the strategies. Conclusion: Using a knowledge-translation framework, information and strategies can be provided to family and professional caregivers to help them optimize residual memory and communication in people with dementia. Future development of the materials, incorporating consumer feedback, will focus on methods for enabling wider dissemination. | Other (Service description); Not specified; MEDIUM |
| Smits CHM, de Lange J, Droes R-M, Meiland F, Vernooij-Dassen M and Pot AM | 2007 | Effects of combined intervention programmes for people with dementia living at home and their caregivers: a systematic review | This study reviews the evidence for effects of combined intervention programmes for both the informal caregiver and the person with dementia. Electronic databases and key articles were searched for effect studies of combined programmes, published between January 1992 and February 2005. The resulting 52 reports were scored according to set inclusion criteria. Twenty five reports relating to 22 programmes met the inclusion criteria. Various aspects of caregivers' mental health and burden were studied. Best results were obtained regarding general mental health. Other aspects often showed modest and varying results. Caregivers' competence was less often addressed. The effects on the cognitive and physical functioning, behavioural problems and survival of the persons with dementia were modest and inconsistent, whereas their mental health is positively affected and admittance to long stay care is often delayed. Combined programmes may improve some, not all, aspects of functioning for caregiver and person with dementia. Care professionals must define their programme goals and target groups before advising their clients on a combined programme. Research may focus on the effects of programmes that were introduced fairly recently and on subgroups of caregivers (female caregivers, depressed caregivers and people with dementia, and minorities). | Literature review; International; HIGH |
| Spijker A, Vernooij-Dassen M, Vasse E, Adang E, Wollersheim H, Grol R and Verhey F | 2008 | Effectiveness of nonpharmacological interventions in delaying the institutionalization of patients with dementia: A meta-analysis | Contemporary healthcare policies are designed to shape the conditions that can help delay the institutionalization of patients with dementia. This can be done by developing support programs that minimize healthcare risks for the patients with dementia and their informal caregivers. Many support programs have been developed, and some of them are effective, but there has been no systematic review with a meta-analysis of all types of nonpharmacological support programs with odds of institutionalization or time to institutionalization as an outcome measure. A systematic review with a meta-analysis was therefore conducted to estimate the overall effectiveness of nonpharmacological support programs for caregivers and patients with dementia that are intended to delay institutionalization. Thirteen support programs with a total of 9,043 patients were included in the meta-analyses. The estimated overall effectiveness suggests that these programs significantly decrease the odds of institutionalization (odds ratio (OR)=0.66, 95% confidence interval (CI)=0.43-0.99, P=.05) and significantly increase the time to institutionalization (standardized mean difference (SMD)=1.44, 95% CI=0.07-2.81, P=.04). A meta-analysis of the best-quality studies still showed a positive significant result for the odds of institutionalization (OR=0.60, 95% CI=0.43-0.85, P=.004), although the time to institutionalization was no longer significant (SMD=1.55, 95% CI=-0.35- 3.45, P=.11). The analysis of the intervention characteristics showed that actively involving caregivers in making choices about treatments distinguishes effective from ineffective support programs. Further investigation should be directed toward calculating the potential efficiency of these support programs by applying net-benefit or cost-effectiveness analysis. | Literature review; International; HIGH |
| Stern C and Munn Z | 2010 | Cognitive leisure activities and their role in preventing dementia: a systematic review | Background: Dementia inflicts a tremendous burden on the healthcare system. Identifying protective factors or effective prevention strategies may lead to considerable benefits. One possible strategy mentioned in the literature relates to participation in cognitive leisure activities. Aim: To determine the effectiveness of cognitive leisure activities in preventing Alzheimer's and other dementias among older adults. Inclusion criteria: Types of participants. Adults aged at least 60 years of age with or without a clinical diagnosis of dementia that resided in the community or care setting. Types of interventions. Cognitive leisure activities defined as activities that required a mental response from the individual taking part in the activity (e.g. reading). Types of outcomes. The presence or absence of dementia was the outcome of interest. Types of studies. Any randomised controlled trials, other experimental studies, as well as cohort, case control and cross-sectional studies were considered for inclusion. Search strategy. A search for published and unpublished studies in the English language was undertaken with no publication date restriction. Methodological quality: Each study was appraised independently by two reviewers using the standard Joanna Briggs Institute instruments. Data collection and analysis: Information was extracted from studies meeting quality criteria using the standard Joanna Briggs Institute tools. Because of the heterogeneity of populations and interventions, meta-analyses were not possible and results are presented in narrative form. Results: There were no randomised controlled trials located that met inclusion criteria. Thirteen observational studies were included in the review; the majority were cohort design. Because of the heterogeneity of interventions, the study design, the way in which they were grouped and the different stages of life they were measured at, statistical pooling was not appropriate. Studies were grouped by stage of adult life participation when interventions were undertaken, that is, early adulthood, middle adulthood and late life. Five out of six studies showed a positive association between participating in activities and a reduced risk of developing Alzheimer's disease and other dementias when interventions were undertaken in middle adulthood and six out of seven studies produced a positive association for late life participation. Results indicated that some activities might be more beneficial than others; however, results should be interpreted with caution because of the subjective nature of activity inclusion. Conclusion: 1. Actively participating in cognitive leisure activities during mid- or late life may be beneficial in preventing the risk of Alzheimer's disease and other dementias in the elderly; however, the evidence is currently not strong enough to infer a direct causal relationship. 2. Participating in selected cognitive leisure activities may be more favourable than others but currently there is no strong evidence to recommend one over the other. | Literature review; International; HIGH |
| Teixeira C, Gobbi L, Corazza D, Stella F, Costa J and Gobbi S | 2012 | Non-pharmacological interventions on cognitive functions in older people with mild cognitive impairment (MCI) | Mild cognitive impairment (MCI) can be a stage of pre-dementia. There is no consensus about pharmacological treatment for this population, so it is important to structure non-pharmacological interventions for increasing their cognitive reserve. We intended to analyze the effects of non-pharmacological interventions in the cognitive functions in older people with MC, in form of a systemic review. Data sources were the Web of Science, Biological Abstracts, Medline, Pub Med, EBSCOHost, Scirus and Google Scholar. All studies were longitudinal trials, with MCI sample, aged>60 years, community-dwelling, and having cognitive functions as dependent variable. Seven studies, from 91 previously selected ones, were identified according to the inclusion criteria. Six studies used cognitive intervention, improving memory and one study used physical activity as intervention, improving executive functions. The results show evidence that physical activity and cognitive exercise may improve memory and executive functions in older people with MCI. But yet, more controlled studies are needed to establish a protocol of recommendations regarding the systemization of exercise, necessary to produce benefits in the cognitive functioning in older people with MCI. | Literature review; International; MEDIUM |
| Thomas H | 2010 | Attitudes of primary care team to diagnosing dementia | Healthcare professionals in primary care are gatekeepers to specialist services and are important in terms of ensuring access to community support and appropriate referral for the sizable number of older people with mental health problems. This literature review explores the role of primary care professionals, particularly GPs and practice nurses, in diagnosing and managing patients with dementia. It recommends that education and training are required to raise awareness of the importance of accurate diagnosis and management in primary care. | Literature review; International; MEDIUM |
| Travers C and Martin-Khan M | 2009 | Barriers and enablers of health promotion, prevention and early intervention in primary care: Evidence to inform the Australian national dementia strategy | A comprehensive literature review was undertaken to: (i) identify and summarise the research evidence regarding barriers and enablers of health promotion, prevention and early intervention (PPEI) in primary care to reduce the risk of chronic disease in the older population; and (ii) use this evidence to make recommendations to inform the Australian national dementia prevention strategy around the translation of evidence-based care into practice. PPEI activities in primary care have the potential to not only reduce the prevalence and impact of a number of chronic diseases, but may also prevent or slow the onset of dementia given the apparent overlap in risk factors. While sizeable gaps exist regarding the most effective ways to promote the adoption of these activities, limited evidence suggests that, to be effective, PPEI activities should be quick and easy to administer, have a sound rationale and be readily incorporated into existing work processes. | Literature review; International; MEDIUM |
| Villars H, Oustric S, Andrieu S, Baeyens JP, Bernabei R, Brodaty H, Brummel-Smith K, Celafu C, Chappell N, Fitten J, Frisoni G, Froelich L, Guerin O, Gold G, Holmerova I, Iliffe S, Lukas A, Melis R, Morley JE, Nies H, Nourhashemi F, Petermans J, Ribera Casado J, Rubenstein L, Salva A, Sieber C, Sinclair A, Schindler R, Stephan E, Wong RY and Vellas B | 2010 | The primary care physician and Alzheimer's disease: an international position paper | This paper aims to define the role of the primary care physician (PCP) in the management of Alzheimer's disease (AD) and to propose a model for a work plan. The proposals in this position paper stem from a collaborative work of experts involved in the care of AD patients. It combines evidence from a literature review and expert's opinions who met in Paris, France, on July 2009 during the International Association of Geriatrics and Gerontology (IAGG) World Congress. The PCP's intervention appears essential at many levels: detection of the onset of dementia, diagnostic management, treatment and follow-up. The key role of the PCP in the management of AD, as care providers and care planners, is consolidated by the family caregiver's confidence in their skills. In primary care practice the first step is to identify dementia. The group proposes a "case finding" strategy, in target situations in which dementia should be detected to allow, secondarily, a diagnosis of AD, in certain cases. We propose that the PCP identifies 'typical' cases. In typical cases, among older subjects, the diagnosis of "probable AD" can be done by the PCP and then confirm by the specialist. While under-diagnosis of AD exists, so does under-disclosure. Disclosure to patient and family should be done by both specialist and PCP. Then, the PCP has a central role in management of the disease with the general objectives to detect, prevent and treat, when possible, the complications of the disease (falls, malnutrition, behavioural and psychological symptoms of dementia). The PCP needs to give basic information to the caregiver on respite care and home support services in order to prevent crisis situations such as unplanned institutionalisation and "emergency" hospital admission. Finally, therapeutic research must be integrated in the daily practice of PCP. It is a matter of patients' right to benefit from access to innovation and clinical research whatever his age or diseases, while of course fully respecting the rules and protective measures that are in force. | Literature review; International; MEDIUM |
| Viola LF, Nunes PV, Yassuda MS, Aprahamian I, Santos FS, Santos GD, Brum PS, Borges SM, Oliverira AM, Chaves GFS, Ciasca EC, Ferreira RCR, de Paula VJR, Takeda OH, Mirandez RM, Watari R, Falcao DVA, Cachioni M and Forlenza OV | 2011 | Effects of a multidisciplinar cognitive rehabilitation program for patients with mild Alzheimer's disease | OBJECTIVE: To evaluate the effects of a multidisciplinary rehabilitation program on cognition, quality of life, and neuropsychiatric symptoms in patients with mild Alzheimer's disease. METHOD: The present study was a single-blind, controlled study that was conducted at a university-based day-hospital memory facility. The study included 25 Alzheimer's patients and their caregivers and involved a 12-week stimulation and psychoeducational program. The comparison group consisted of 16 Alzheimer's patients in waiting lists for future intervention. INTERVENTION: Group sessions were provided by a multiprofessional team and included memory training, computer-assisted cognitive stimulation, expressive activities (painting, verbal expression, writing), physiotherapy, and physical training. Treatment was administered twice a week during 6.5-h gatherings. MEASUREMENTS: The assessment battery comprised the following tests: Mini-Mental State Examination, Short Cognitive Test, Quality of Life in Alzheimer's disease, Neuropsychiatric Inventory, and Geriatric Depression Scale. Test scores were evaluated at baseline and the end of the study by raters who were blinded to the group assignments. RESULTS: Measurements of global cognitive function and performance on attention tasks indicated that patients in the experimental group remained stable, whereas controls displayed mild but significant worsening. The intervention was associated with reduced depression symptoms for patients and caregivers and decreased neuropsychiatric symptoms in Alzheimer's subjects. The treatment was also beneficial for the patients' quality of life. CONCLUSION: This multimodal rehabilitation program was associated with cognitive stability and significant improvements in the quality of life for Alzheimer's patients. We also observed a significant decrease in depressive symptoms and caregiver burden. These results support the notion that structured nonpharmacological interventions can yield adjunct and clinically relevant benefits in dementia treatment. | Controlled Clinical Trial (CCT); Brazil; MEDIUM |
| Wilz G and Fink-Heitz M | 2008 | Assisted vacations for men with dementia and their caregiving spouses: Evaluation of Health related effects | Purpose: In this study, we conducted the first evaluation of assisted vacations for persons with dementia and their caregivers in the field of caregiving research. Design and Methods: We used a quasi-experimental, two-group, repeated measures design with two measuring times (preintervention, 3-month follow-up) to examine whether assisted vacations lead to a reduction in physical complaints and symptoms of depression in family caregivers. The sample consisted of 29 caregiving wives who were providing at-home care for husbands suffering from dementia. We selected the participants in the control group from a waiting list. Outcome measurements were made with the Giessen Subjective Complaints List and the Beck Depression Inventory. Results: The overall emotional and physical states of the participants in the intervention group showed significant improvements in comparison with those of the control group 3 months after the first interview. Implications: The results show that assisted vacations can have both immediate and longer lasting positive effects on participants' health. Assisted vacations can therefore be a way of diminishing the risk of stress disorders for caregiving spouses. Making assisted vacations available to people with dementia and their caregivers is a worthwhile goal for community support facilities working to reduce caregiver burden. | Controlled Clinical Trial (CCT); Germany; MEDIUM |
| Zucchero RA, Hooker E and Larkin S | 2010 | An interdisciplinary symposium on dementia care improves student attitudes toward health care teams | Background: Interdisciplinary teams are sometimes used in the provision of health care to populations who present with complicated needs, such as older adults experiencing dementia. Moreover, there is an international consensus that health care students should receive training in interdisciplinary care. Methods: 157 health care students from Xavier University's College of Social Sciences, Health, and Education in Cincinnati, U.S.A. participated in a five-hour symposium on an interdisciplinary approach to treating older adults with dementia. The Attitudes Toward Health Care Teams Scale (ATHCTS; Heinemann et al., 1999) was used to assess student attitudes before and after the symposium. Results: A paired-sample t-test was conducted to compare pre and post-test ATHCTS overall and subscale scores. There was a statistically significant increase in the overall pre-post ATHCTS scores and Quality of Care/Process Subscale scores. There was a significant decrease in the Physician Centrality Subscale scores. Conclusions: The findings suggest that, after the symposium, participants reported more positive overall attitudes about health care teams, and about the quality of care provided by such teams and the teamwork to achieve good patient care. Participants also displayed a decrease in their beliefs about how essential physicians are as leaders of health care teams. These results affirm the use of a brief interdisciplinary educational approach in changing student attitudes about the use of health care teams. Students who develop more positive attitudes about working on an interdisciplinary health care team recognize the team's value and therefore may be more receptive to and effective in working as professional team members in the future. | Other (Non-Controlled Before and After study); USA); MEDIUM |
